# Pentafluorosulfanyl-containing Triclocarban Analogs with Potent Antimicrobial Activity

**DOI:** 10.3390/molecules23112853

**Published:** 2018-11-02

**Authors:** Eugènia Pujol, Núria Blanco-Cabra, Esther Julián, Rosana Leiva, Eduard Torrents, Santiago Vázquez

**Affiliations:** 1 Laboratori de Química Farmacèutica (Unitat Associada al CSIC), Facultat de Farmàcia i Ciències de l′Alimentació, and Institute of Biomedicine (IBUB), Universitat de Barcelona, Av. Joan XXIII, 27-31, 08028 Barcelona, Spain; epujol@ub.edu (E.P.); rosana.leiva58@gmail.com (R.L.); 2 Bacterial Infections and Antimicrobial Therapies, Institute for Bioengineering of Catalonia (IBEC), The Barcelona Institute of Science and Technology, Baldiri Reixac 15-21, 08028 Barcelona, Spain; nblanco@ibecbarcelona.eu; 3 Departament de Genètica i de Microbiologia, Facultat de Biociències, Universitat Autònoma de Barcelona, 08193 Bellaterra, Spain; esther.julian@uab.cat

**Keywords:** antibacterial, Gram-positive, *N*,*N*′-diarylureas, pentafluorosulfanyl, *Staphylococcus aureus*, triclocarban

## Abstract

Concerns have been raised about the long-term accumulating effects of triclocarban, a polychlorinated diarylurea widely used as an antibacterial soap additive, in the environment and in human beings. Indeed, the Food and Drug Administration has recently banned it from personal care products. Herein, we report the synthesis, antibacterial activity and cytotoxicity of novel *N*,*N*′-diarylureas as triclocarban analogs, designed by reducing one or more chlorine atoms of the former and/or replacing them by the novel pentafluorosulfanyl group, a new bioisostere of the trifluoromethyl group, with growing importance in drug discovery. Interestingly, some of these pentafluorosulfanyl-bearing ureas exhibited high potency, broad spectrum of antimicrobial activity against Gram-positive bacterial pathogens, and high selectivity index, while displaying a lower spontaneous mutation frequency than triclocarban. Some lines of evidence suggest a bactericidal mode of action for this family of compounds.

## 1. Introduction

The presence of *N*,*N*′-diarylureas in medicinal chemistry is of great importance due to their broad spectrum of biological activities. They have been widely studied in the field of insecticides [1] and infectious diseases such as malaria [2], schistosomiasis and tuberculosis [3,4], immunology [5,6] and oncology [7], among others. Triclocarban (TCC) is a *N*,*N*′-diarylurea commonly used as an antimicrobial agent in personal care products such as bar soaps, deodorants, detergents, and other disinfectants [8]. In recent years, public concerns have been raised regarding its potential toxicological effects in mammals and its environmental accumulation [9,10,11]. Studies show that, when applied in the skin, this antibacterial is absorbed through it and can even be detected in human plasma, urine, and milk [10,11,12,13]. Furthermore, TCC has been recognized as an endocrine disruptor at high concentrations [9,10], resulting in hormonal effects, and more recently, the mechanisms through which it can alter cardiac function have been elucidated [14]. Moreover, it has been reported that TCC is a potent inhibitor of soluble epoxide hydrolase, which may lead to alterations in human physiology [15]. Due to its three chlorine atoms, the biodegradation of TCC is so slow that it can persist in the environment for years [11,14]. Indeed, studies have demonstrated that it accumulates in aquatic habitats [9,13,16,17,18,19]. On the basis of the above, the safety of this antimicrobial agent in long-term daily use has not yet been demonstrated. Since 2017, the Food and Drug Administration (FDA) has banned the use of TCC and triclosan, a related antimicrobial agent, in consumer products. Therefore, the development of alternative agents to TCC and triclosan for the use in consumer products is an appealing topic to researchers [20].

The trifluoromethyl group is commonly used in medicinal chemistry as a bioisosteric replacement of chlorine atoms. Therefore, it is not surprising that a few *N*,*N*′-diarylureas containing a trifluoromethyl unit also displayed promising antibacterial activities [1]; this is the case for cloflucarban (TFC, 3-trifluoromethyl-4,4′-dichlorocarbanilide), a trifluoromethyl-substituted diarylurea that shares not only the same spectrum of activity with TCC, but also a similar pattern of absorption, distribution, excretion and toxicity [21,22,23].

Very recently, a new bioisoster of the trifluoromethyl unit has been introduced in medicinal chemistry: the pentafluorosulfanyl group (SF_5_), a relatively new polyfluorinated substituent that has been applied in agro and material chemistry [24,25]. When compared to its isostere trifluoromethyl group, the SF_5_-group is considered a “super-trifluoro-methyl group”, since it bears advantageous properties, including tetragonal bipyramidal shape, high electronegativity (3.65 vs. 3.36 for the trifluoromethyl group), high lipophilicity, large steric volume (slightly less than that of *tert*-butyl but greater than trifluoromethyl), and confirmed hydrolytic and chemical stability [24,26,27,28,29,30,31]. Due to its unique properties, the presence of SF_5_ in medicinal chemistry has been increasing in the last decade, to the extent that it is nowadays considered to be an extremely attractive substituent in medicinal applications [24,25,26,27]. Indeed, a new antimalarial SF_5_-containing drug, DSM-265, has recently entered clinical trials [32].

Despite the increasing research around the pentafluorosulfanyl group, little is known about the environmental impact of SF_5_-containing molecules [33]. Among the performed studies, it has been shown that the degradation of SF_5_-substituted aryl compounds results in environmentally-mild products [34].

**Figure 1:**
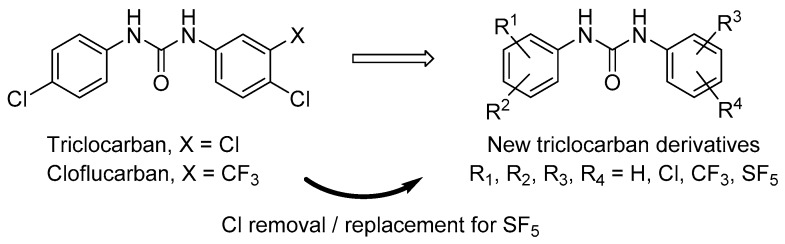
Chemical structures of triclocarban, cloflucarban, and general structure of the new *N*,*N*′-diarylureas presented in this work.

Bearing in mind that the presence of SF_5_ in this field is increasing in the last few years [24], and that it has a more environmentally-benign profile compared to the chlorine atom, the aim of the present work was to introduce this novel group on the *N*,*N*′-diarylurea scaffold in order to obtain new antimicrobial agents (Figure 1). Herein, we report the design, synthesis, and biological evaluation of novel SF_5_-analogs of TCC, which could be a good starting point for a new generation of antibacterial ingredients.

## 2. Results and Discussion

### 2.1. Chemistry

**Scheme 1:**
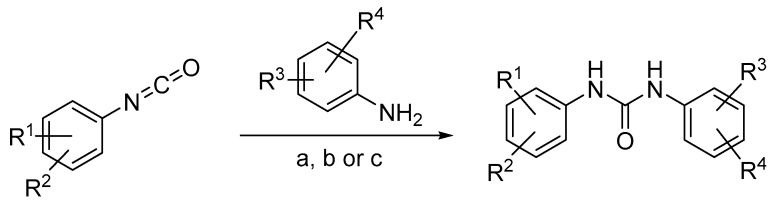
Synthesis of novel diarylureas. (a) CH_2_Cl_2_, rt, overnight; (b) pyridine, rt, 1 h; (c) *n*-BuLi, anhydrous THF, −78 °C to rt, overnight. See Figure 2, material and methods section and supplementary materials for details.

**Figure 2:**
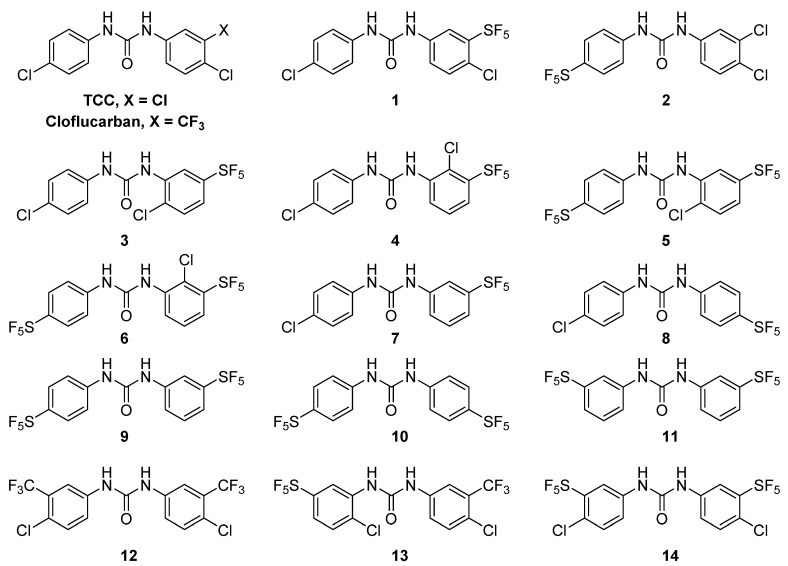
Pentafluorosulfanyl ureas **1**–**14**. Compounds **1** to **6** and **8** are new. Compounds **9**, **11**, **13** and **14** have been recently disclosed by us in a patent application [35]. Compounds **7** [36], **10** [37] and **12** [1] were previously known.

All the *N*,*N*′-diarylureas were prepared following a quite simple and straightforward procedure, which consisted of the coupling of phenyl isocyanates with the corresponding anilines under three slightly different reaction conditions. In turn, aryl isocyanates were commercially available or formed in situ from their corresponding anilines by reaction with triphosgene in the presence of triethylamine as a base, in an organic solvent such as toluene. Compounds were synthesized in low to moderate yields, since it was observed that dimerization products from the starting aromatic amines were often predominant (Scheme 1 and Figure 2). The structure of these diarylureas was confirmed by IR, ^1^H, ^13^C, and ^19^F NMR, elemental analysis, and/or HRMS (see material and methods section and supplementary materials for further details).

### 2.2. Antibacterial Activity, Selectivity Index and Structure-Activity Relationships

The antibacterial activity of the final compounds against several Gram-positive and Gram-negative bacterial pathogens was evaluated by determination of the minimum inhibitory concentration values (MIC_50_) (Table 1). To check if these molecules had a safer profile than TCC, and to demonstrate their possible use as topic antibacterial compounds, we next evaluated their toxicity (CC_50_, cytotoxic concentration 50%) on eukaryotic cells using a macrophages viability measure. In agreement with previous reports, TCC and cloflucarban displayed antibacterial activity in Gram-positive bacteria (Table 1), whereas no activity was detected against Gram-negative pathogens (data not shown) [38]. Similarly, the new analogs synthesized in this work did not show antibacterial activity against the Gram-negative pathogens *P. aeruginosa* and *E. coli* (data not shown). It is worthy of note that while the activity of TCC and cloflucarban in Gram-positive bacteria was restricted to the *Staphylococcus* genera included in this work (*S. aureus*, *S. epidermidis*, and a *S. aureus* methicillin resistant clinical isolate), most of the newly-designed pentafluorosulfanyl derivatives had a broader antimicrobial activity spectrum than TCC and cloflucarban, being active against *Streptococcus mutants* and *Enterococcus faecalis* bacterial strains. Remarkably, although clinical isolates are usually more resistant to antibiotics, the clinical isolate *S. aureus* methicillin resistant (MRSA) showed almost the same antimicrobial sensitivity as the other laboratory strain, indicating no mechanisms of resistance to these compounds in this clinical isolate (Table 1).

Taking into account the very similar activities and cytotoxicities of TCC and cloflucarban, we first synthesized pentafluorosulfanyl analog **1**, that maintained the potency of the parent compounds against the *Staphylococcus* genera, and was additionally active against *S. mutants* and *E. faecalis* bacterial strains. However, **1** was more cytotoxic than TCC and cloflucarban, resulting in lower selectivity indexes. A similar trend was observed with compound **2**. Notwithstanding, the cytotoxicity does not seem to be directly related with the introduction of the SF_5_ group, since two isomers of **1**, ureas **3** and **4**, were less cytotoxic, although they were not active against *S. mutants* and *E. faecalis*. Further replacement of the Cl of the left-hand ring in **3** and **4** for SF_5_, as in **5** and **6**, respectively, restored the activity against *S. mutants* and *E. faecalis* but, again, in line with an increase in cytotoxicity.

While these initial results showed that the replacement of a chlorine atom for the pentafluorosulfanyl group was indeed a promising approach, the higher cytotoxicity of several of these novel derivatives remained as a worrying issue. Hence, we next evaluated the removal of a chlorine atom of the aforementioned compounds. Ureas **7** and **8**, conceptually generated by the removal of a chlorine atom in **1** and **2**, respectively, were endowed with very similar antimicrobial activities and cytotoxicities than TCC. Finally, replacement of the remaining chlorine atom in **7** by a second pentafluorosulfanyl group led to **9**, a compound with similar cytotoxicity than TCC but with broader spectrum of action. Similarly, moving from **8** to **10** gave rise to a very promising compound, active against the five Gram-positive bacterial strains studied and with selectivity indexes of up to 412. Isomer **11**, although being also an interesting compound, was less potent than **10**.

Finally, starting from cloflucarban, we briefly evaluated the introduction of a fourth electron withdrawing group. The three evaluated compounds, **12**–**14**, were active against the five Gram-positive bacterial strains with cytotoxicities similar or slightly better than those of TCC and cloflucarban. Overall, it seems that the introduction of a fourth electron withdrawing group is not worthwhile.

### 2.3. Diarylureas Show a Bacteriolytic Mode of Action

To better understand how the different compounds affect the viability of *S. aureus*, bacterial cells were stained using the Live/Dead viability test and visualized under the fluorescent microscope. This experiment allows us to observe the membrane integrity, since Syto9 green only labels bacterial DNA if the cells are viable and propidium iodide can only enter bacteria cells with a damaged membrane, staining the whole cell red.

**Figure 3:**
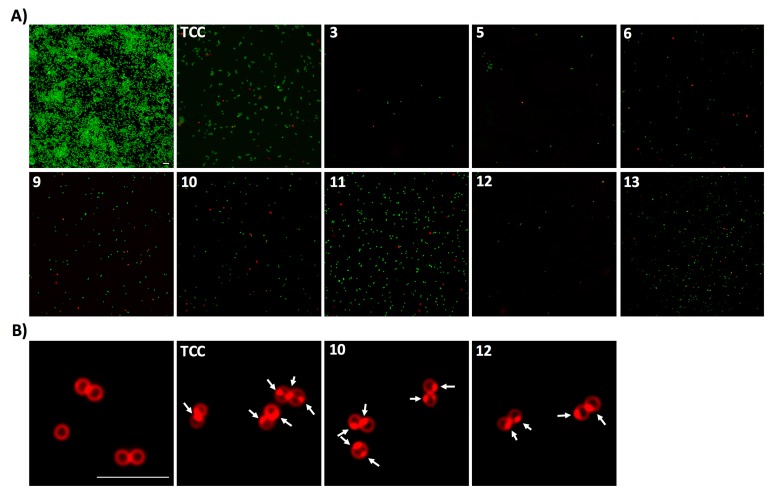
(**A**) Live/Dead analysis of *S. aureus*. Green fluorescence indicates live cells, and red fluorescence suggests dead cells. (**B**) Membrane damage analysis by FM 4-46 staining. Scale bar correspond to 5 µm.

Treatment of bacterial cells during 4 h with TCC and novel compounds **3**, **5**, **6** and **9**–**13**, increased the proportion of non-viable cells and diminished the total cells, particularly with compounds **3**, **5**, **6** and **12**, suggesting a bacteriolytic mode of action for this chemical family of compounds (Figure 3A).

Additionally, we microscopically visualized the changes in bacterial cell integrity by label plasma membrane of living cells using the vital stain FM 4-64 (Figure 3B). Staining the untreated cells resulted in uniform membrane accumulations. On the other hand, cells treated with TCC and compounds **10** and **12** resulted in stained membrane blebs, possibly originating from severe membrane deformations, which is an indication that the primary antibacterial mode of action of these compounds may involve membrane damage.

### 2.4. Antimicrobial Activity of the New Diarylureas on Removal of Biofilms in Catheters and on Disinfection on Contaminated Surfaces

**Figure 4:**
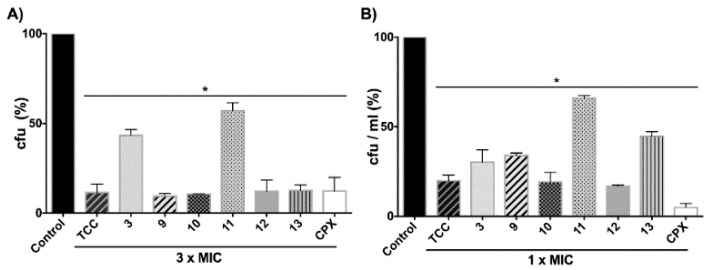
(**A**) Antibacterial effect of compounds on disinfection of contaminated surfaces by *S. aureus* and (**B**) on biofilms growing on catheters. * indicates statistically significant difference to the control (untreated) experiment (*p*-value < 0.05 in pairwise *t*-test calculated with GraphPad 6.0). CPX was used as a control in all antimicrobial experiments, with a MIC_50_ of 0.5 µg/mL against all the bacteria pathogens tested. cfu, colony-forming unit.

Given that TCC is widely used as an antimicrobial agent in personal care products (soaps, deodorants, detergents, and others), we tested the capacity of TCC derivatives in disinfecting a contaminated surface area. As shown in Figure 4A, compounds **9**, **10**, **12** and **13** have substantial capacity to remove a contaminated glass area at the same level as TCC, indicating the same capacity of these new compounds to be used as a disinfectant.

Furthermore, *S. aureus* is one of the leading causes of catheter-related bacteremia due to the colonization of surgical devices in hospitals by a biofilm form of growth. For that reason, it is highly important to develop protocols and new methodologies to treat and remove pre-existing biofilms in medical devices, especially in catheters. Hence, we studied whether the selected compounds were active in terms of removing biofilms formed in a catheter mode of infection by this bacteria, and we compared the results with TCC and the antibiotic ciprofloxacin (CPX), which is endowed with potent anti-biofilm activity. It is worth noting that compounds **10** and **12** showed the same percentage of biofilm removing capacity in catheters than TCC, whereas the reduction produced by these compounds is similar to the reduction of biofilm due to the treatment with ciprofloxacin (Figure 4B).

### 2.5. New Compounds Show Less Spontaneous Mutation Rates Compared to TCC

Increasing resistance to antimicrobials is an enormous problem for our society. For this reason, during the development of new antimicrobials, it is common to assess the frequency of spontaneous, resistant mutants within a bacterial population to warrant that this is not a serious issue that compromises further development [39]. We therefore studied the spontaneous mutation rates of *S. aureus* induced by TCC and selected diarylureas as described in the materials and methods section. As indicated in Table 2, TCC had a mutation rate of 4 × 10^−9^, similar to that of **12** (5 × 10^−10^). Interestingly, compounds **3**, **9**, **10** and **13**, at the same concentration as TCC, showed no induced mutation rates, which indicated the difficulty that such compounds have in inducing any spontaneous resistance in *S. aureus*.

## 3. Materials and Methods

### 3.1. Chemical Synthesis

#### 3.1.1. General Methods

Commercially-available reagents and solvents were used without further purification unless stated otherwise. 2-chloro-3-(pentafluoro-λ^6^-sulfanyl)aniline, 2-chloro-5-(pentafluoro-λ^6^-sulfanyl)aniline and 4-chloro-3-(pentafluoro-λ^6^-sulfanyl)aniline were synthesized according to a reported procedure [40]. Preparative normal phase chromatography was performed on a CombiFlash Rf 150 (Teledyne Isco, Lincoln, NE, USA) with pre-packed RediSep Rf silica gel cartridges. Thin-layer chromatography was performed with aluminum-backed sheets with silica gel 60 F_254_ (Merck, Darmstadt, Germany, ref 1.05554), and spots were visualized with UV light and 1% aqueous solution of KMnO_4_. Melting points were determined in open capillary tubes with a MFB 595010M Gallenkamp. 400 MHz ^1^H, 100.6 MHz ^13^C and 376.5 MHz ^19^F NMR spectra were recorded on a Varian Mercury 400 or on a Bruker 400 Avance III spectrometers. Then, 500 MHz ^1^H NMR spectra were recorded on a Varian Inova 500 spectrometer. The chemical shifts are reported in ppm (δ scale) relative to internal tetramethylsilane, and coupling constants are reported in Hertz (Hz). Assignments given for the NMR spectra of the compounds have been carried out on the basis of DEPT, COSY ^1^H/^1^H (standard procedures), and COSY ^1^H/^13^C (gHSQC and gHMBC sequences) experiments. IR spectra were run on Perkin-Elmer Spectrum RX I (Waltham, MA, USA) or Nicolet Avatar 320 FT-IR spectrophotometers. Absorption values are expressed as wave-numbers (cm^−1^); only significant absorption bands are given. High-resolution mass spectrometry (HRMS) analyses were performed with an LC/MSD TOF Agilent Technologies spectrometer. The elemental analyses were carried out in a Flash 1112 series Thermofinnigan elemental microanalyzator (A5) to determine C, H, N and S. HPLC/MS were determined with a HPLC Thermo Ultimate 3000SD (Thermo Scientific Dionex, Waltham, MA, USA) coupled to a photodiode array detector DAD-3000 (Thermo Scientific Dionex, Waltham, MA, USA) and mass spectrometer LTQ XL ESI-ion trap (Thermo Scientific, Waltham, MA, USA) with Xcalibur v2.2 acquisition software (Thermo Scientific, Waltham, MA, USA) (HPLC-PDA-MS). 5 µL of sample 0.5 mg/mL in methanol were injected, using a ZORBAX Extend-C18 3.5 µm 2.1 × 50 mm column (Agilent, Santa Clara, CA, USA) at 30 °C. The mobile phase was a mixture of A = formic acid 0.05% in water and B = formic acid 0.05% in acetonitrile with the method described as follows: flow 0.6 mL/min, 5% B-95% A 3 min, 100% B 4 min, 95% B-5% A 8 min. Purity is given as % of absorbance at 254 nm; UV-Vis spectra were collected every 0.2 s between 650 and 275 nm; data from mass spectra were analyzed by electrospray ionization in positive mode every 0.3 s between 50 and 1000 Da. The analytical samples of all of the new compounds, which were subjected to pharmacological evaluation, possessed a purity of ≥95%, as evidenced by either their elemental analyses or their HPLC-MS.

#### 3.1.2. General Procedures for the Synthesis of Aryl Isocyanates

A solution of aniline (1 Eq) in toluene (5 or 10 mL) was treated with triphosgene (0.5 Eq). Triethylamine (1 Eq) was immediately added, and the reaction mixture was stirred at 70 °C for 2 h. Afterwards pentane (1 mL) was added, and a white precipitate was formed. The mixture was filtered and pentane was evaporated under vacuum at room temperature to give the corresponding isocyanate in toluene solution that was used in the next step without further purification.

#### 3.1.3. General Procedure A for the Synthesis of Ureas **2**–**6**

To a solution of the previously-obtained aryl isocyanate was added the substituted aniline in dichloromethane (5 mL). The suspension was stirred at room temperature overnight. Crude ureas were purified by column chromatography or were crystallized in the appropriate solvent.

#### 3.1.4. General Procedure B for the Synthesis of Ureas **1**, **7** and **8**

The commercially-available 4-chlorophenylisocyanate (1 Eq) was added with stirring to a solution of the required pentafluorosulfanylaniline (1 Eq) in pyridine (1.5 or 2 mL). The mixture was allowed to stand at room temperature for 1 h. Afterwards it was poured into water and the precipitate formed was removed by filtration. The final compounds were purified by crystallization from methanol or by column chromatography (hexane/ethyl acetate mixtures).

#### 3.1.5. General Procedure C for the Synthesis of Ureas **9**–**14**

The aniline was dissolved in anhydrous THF (5 or 12 mL) under argon and cooled to −78 °C on a dry ice in acetone bath. Afterwards 2.5 M *n*-butyllithium in hexanes (1.1, 1.2 or 1.3 Eq) was added dropwise. The reaction mixture was then removed from the dry ice in acetone bath and tempered to 0 °C with an ice bath. The appropriate isocyanate, prepared in the previous step or commercially available, was then stirred under argon and continuously added to the reaction mixture. The mixture was stirred at room temperature overnight. Methanol (4 or 5 mL) was added to quench any unreacted *n*-butyllithium. Purification by column chromatography provided the desired diarylureas.

*1-(4-Chloro-3-(pentafluoro-λ^6^-sulfanyl)phenyl)-3-(4-chlorophenyl)* urea **1**. From 4-chlorophenylisocyanate (151 mg, 0.98 mmol) and 4-chloro-3-(pentafluoro-λ^6^-sulfanyl)aniline (250 mg, 0.98 mmol) in pyridine (1.5 mL) and following general procedure B, a white solid (280 mg) was obtained. Column chromatography (hexane/ ethyl acetate) furnished urea **1** (66 mg, 17%) as a white solid, mp 222–223 °C. IR (KBr) ν: 412, 503, 569, 599, 654, 678, 752, 789, 811, 824, 854, 864, 891, 926, 1012, 1039, 1093, 1127, 1241, 1284, 1300, 1385, 1401, 1479, 1493, 1546, 1576, 1595, 1650, 3084, 3129, 3182, 3298 cm^−1^. ^1^H NMR (400 MHz, DMSO-*d*_6_) δ: 7.34 [m, 2 H, 3′(5′)-H], 7.49 [m, 2 H, 2′(6′)-H], 7.60 (dd, *J* = 8.8 Hz, *J*′ = 2.0 Hz, 1 H, 6-H), 7.64 (d, *J* = 8.8 Hz, 1 H, 5-H), 8.38 (d, *J* = 2.0 Hz, 1 H, 2-H), 9.04 (broad s, 1 H) and 9.30 (broad s, 1 H) (2 NH). ^13^C NMR (100.6 MHz, CD_3_OD) δ: 120.7 (m, CH, C2), 121.9 [CH, C2′(6′)], 122.9 (C, C4), 124.1 (CH, C6), 129.0 (C, C4′), 129.8 [CH, C3′(5′)], 133.9 (CH, C5), 139.0 (C, C1 or C1′), 140.1 (C, C1′ or C1), 154.5 (C, CO). The signal for C3 was not observed. ^19^F NMR (376.5 MHz, DMSO-*d*_6_) δ: 66.8 (d, *J* = 152.3 Hz, 4 F, SF_4_F), 85.9 (quint, *J* = 152.3 Hz, 1 F, SF_4_F). Anal. calcd for C_13_H_9_Cl_2_F_5_N_2_OS·0.1C_5_H_12_: C 39.13, H 2.48, N 6.76, S 7.74. Found: C 38.92, H 2.65, N 6.67, S 7.47.

*1-(3,4-Dichlorophenyl)-3-(4-pentafluoro-λ^6^-sulfanyl)phenyl)* urea **2**. From 3,4-dichlorophenylisocyanate (222 mg, 1.18 mmol) in dichloromethane (3 mL) and 4-(pentafluoro-λ^6^-sulfanyl)aniline (259 mg, 1.18 mmol) in toluene (3 mL) and following general procedure A, urea **2** (130 mg, 30% yield) was obtained as a white solid. The analytical sample was obtained by crystallization from dichloromethane (119 mg), mp (dichloromethane) 226–227 °C. IR (KBr) ν: 614, 667, 694, 825, 849, 859, 1030, 1104, 1133, 1194, 1234, 1265, 1302, 1325, 1378, 1390, 1412, 1477, 1504, 1545, 1595, 1665, 3116, 3205, 3356 cm^−1^. ^1^H NMR (400 MHz, DMSO-*d*_6_) δ: 7.36 (dd, *J* = 12.0 Hz, *J*′ = 4.0 Hz, 1 H, 6-H), 7.53 (d, *J* = 12.0 Hz, 1 H, 5-H), 7.65 [m, 2 H, 2′(6′)-H], 7.81 [m, 2 H, 3′(5′)-H], 7.87 (d, *J* = 4.0 Hz, 1 H, 2-H), 9.13 (broad s, 1 H) and 9.33 (broad s, 1 H) (2 NH). ^13^C NMR (100.6 MHz, DMSO-*d*_6_) δ: 117.8 [CH, C2′(6′)], 118.7 (CH, C6), 119.6 (CH, C2), 123.6 (C, C4), 126.8 [m, CH, C3′(5′)], 130.6 (CH, C5), 131.1 (C, C3), 139.5 (C, C1), 142.8 (C, C1′), 146.2 (m, C, C4′), 152.0 (C, CO). ^19^F NMR (376.5 MHz, DMSO-*d*_6_) δ: 65.1 (d, *J* = 150.8 Hz, 4 F, SF_4_F), 89.2 (quint, *J* = 150.8 Hz, 1 F, SF_4_F). Anal. calcd for C_13_H_9_Cl_2_F_5_N_2_OS: C 38.35, H 2.23, N 6.88, S 7.87. Found: C 38.63, H 2.30, N 6.61, S 7.54.

*1-(2-Chloro-5-(pentafluoro-λ^6^-sulfanyl)phenyl)-3-(4-chlorophenyl)* urea **3**. By following general procedure for the synthesis of aryl isocyanates, 2-chloro-5-(pentafluoro-λ^6^-sulfanyl)aniline (300 mg, 1.18 mmol) in toluene (5 mL) was reacted with triphosgene (175 mg, 0.59 mmol) in the presence of triethylamine (0.16 mL, 1.18 mmol) to afford 2-chloro-5-(pentafluoro-λ^6^-sulfanyl)phenylisocyanate in toluene solution. From this previously-obtained isocyanate and 4-chloroaniline (151 mg, 1.18 mmol) in dichloromethane (5 mL) and following general procedure A, a white gum (443 mg) was obtained. Column chromatography (hexane/ ethyl acetate) furnished urea **3** (226 mg, 47% overall yield) as a white solid, mp 195–196 °C. IR (KBr) ν: 503, 578, 602, 621, 664, 731, 807, 831, 844, 863, 951, 1015, 1065, 1090, 1234, 1285, 1420, 1461, 1492, 1547, 1591, 1645, 1702, 2848, 2925, 3285, 3325 cm^−1^. ^1^H NMR (400 MHz, DMSO-*d*_6_) δ: 7.35 [m, 2 H, 3′(5′)-H], 7.51 [m, 2 H, 2′(6′)-H], 7.54 (dd, *J* = 8.8 Hz, *J*′ = 2.8 Hz, 1 H, 4-H), 7.72 (d, *J* = 8.8 Hz, 1 H, 3-H), 8.84 (d, *J* = 2.8 Hz, 1 H, 6-H), 8.66 (broad s, 1H) and 9.69 (broad s, 1 H) (2 NH). ^13^C NMR (100.6 MHz, DMSO-*d*_6_) δ: 117.5 (m, CH, C6), 120.0 [CH, C2′(6′)], 120.0 (m, CH, C4), 125.2 (C, C2), 126.1 (C, C4′), 128.8 [CH, C3′(5′)], 129.9 (CH, C3), 136.6 (C, C1), 137.9 (C, C1′), 151.4 (quint, ^2^*J*_CF_ = 17.2 Hz, C, C5), 151.9 (C, CO). ^19^F NMR (376.5 MHz, DMSO-*d*_6_) δ: 63.8 (d, *J* = 151.5 Hz, 4 F, SF_4_F), 86.7 (quint, *J* = 151.5 Hz, 1 F, SF_4_F). HRMS-ESI^+^ *m/z* [M + H]^+^ calcd for [C_13_H_9_Cl_2_F_5_N_2_OS + H^+^]: 406.9806, found: 406.9803. HPLC (254 nm): t_R_ = 4.45 min (100%).

*1-(2-Chloro-3-(pentafluoro-λ^6^-sulfanyl)phenyl)-3-(4-chlorophenyl)* urea **4**. By following general procedure for the synthesis of aryl isocyanates, 2-chloro-3-(pentafluoro-λ^6^-sulfanyl)aniline (300 mg, 1.18 mmol) in toluene (5 mL) was reacted with triphosgene (175 mg, 0.59 mmol) in the presence of triethylamine (0.16 mL, 1.18 mmol) to afford 2-chloro-3-(pentafluoro-λ^6^-sulfanyl)phenylisocyanate in toluene solution. From this previously-obtained isocyanate and 4-chloroaniline (151 mg, 1.18 mmol) in dichloromethane (5 mL) and following general procedure A, a white solid (194 mg) was obtained. Column chromatography (hexane/ ethyl acetate) gave urea **4** (40 mg, 8% overall yield) as a white solid, mp 218–219 °C. IR (KBr) ν: 605, 652, 711, 729, 779, 799, 810, 840, 849, 875, 931, 1014, 1054, 1089, 1155, 1224, 1250, 1284, 1302, 1398, 1417, 1463, 1493, 1546, 1594, 1663, 1713, 3217, 3305, 3340 cm^−1^. ^1^H NMR (400 MHz, DMSO-*d*_6_) δ: 7.36 [m, 2 H, 3′(5′)-H], 7.50 [m, 2 H, 2′(6′)-H], 7.56 (t, *J* = 8.4 Hz, 1 H, 5-H), 7.78 (dd, *J* = 8.4 Hz, *J*′ = 1.2 Hz, 1 H, 4-H), 8.35 (dd, *J* = 8.4 Hz, *J*′ = 1.2 Hz, 1 H, 6-H), 8.65 (broad s, 1 H) and 9.63 (broad s, 1 H) (2 NH). ^13^C NMR (100.6 MHz, CD_3_OD) δ: 121.5 [CH, C2′(6′)], 125.1 (m, CH, C4), 126.5 (CH, C6), 128.0 (CH, C5), 129.0 (C, C2), 129.9 [CH, C3′(5′)], 139.1 (C, C1), 139.5 (C, C1′), 152.7 (m, C, C3), 154.3 (C, CO). ^19^F NMR (376.5 MHz, DMSO-*d*_6_) δ: 67.7 (d, *J* = 152.3 Hz, 4 F, SF_4_F), 86.2 (quint, *J* = 152.3 Hz, 1 F, SF_4_F). Anal. calcd for C_13_H_9_Cl_2_F_5_N_2_OS: C 38.35, H 2.23, N 6.88, S 7.87. Found: C 38.71, H 2.36, N 6.63, S 7.60.

*1-(2-Chloro-5-(pentafluoro-λ^6^-sulfanyl)phenyl)-3-(4-(pentafluoro-λ^6^-sulfanyl)phenyl)* urea **5**. By following general procedure for the synthesis of aryl isocyanates, 2-chloro-5-(pentafluoro-λ^6^-sulfanyl)aniline (500 mg, 1.96 mmol) in toluene (10 mL) was reacted with triphosgene (290 mg, 0.98 mmol) in the presence of triethylamine (0.27 mL, 1.96 mmol) to afford 2-chloro-5-(pentafluoro-λ^6^-sulfanyl)phenylisocyanate in toluene solution. From this previously-obtained isocyanate and 4-(pentafluoro-λ^6^-sulfanyl)aniline (272 mg, 1.07 mmol) in dichloromethane (5 mL) and following general procedure A, a yellowish gum (443 mg) was obtained. Column chromatography (hexane/ ethyl acetate) gave urea **5** (55 mg, 10% overall yield) as a white solid, mp 224–225 °C. IR (KBr) ν: 582, 598, 647, 665, 723, 805, 828, 848, 860, 949, 1043, 1063, 1105, 1196, 1239, 1272, 1288, 1327, 1419, 1466, 1506, 1522, 1560, 1597, 1618, 1670, 1685, 3094, 3141, 3209, 3383 cm^−1^. ^1^H NMR (400 MHz, DMSO-*d*_6_) δ: 7.59 (dd, *J* = 8.8 Hz, *J*′ = 2.8 Hz, 1 H, 4-H), 7.67 [m, 2 H, 2′(6′)-H], 7.75 (d, *J* = 8.8 Hz, 1 H, 3-H), 7.84 [m, 2 H, 3′(5′)-H], 8.82 (d, *J* = 2.8 Hz, 1 H, 6-H), 8.78 (broad s, 1 H) and 10.06 (broad s, 1 H) (2 NH). ^13^C NMR (100.6 MHz, CD_3_OD) δ: 119.0 [CH, C2′(6′)], 119.6 (quint, ^3^*J*_CF_ = 5.2 Hz, CH, C6), 121.8 (quint, ^3^*J*_CF_ = 4.4 Hz, CH, C4), 126.9 (C, C2), 128.1 [quint, ^3^*J*_CF_ = 4.7 Hz, CH, C3′(5′)], 130.6 (CH, C3), 137.6 (C, C1), 143.6 (C, C1′), 149.1 (m, C, C4′ or C5), 153.65 (m, C, C5 or C4′), 153.70 (C, CO). ^19^F NMR (376.5 MHz, CD_3_OD) δ: 63.8 (d, *J* = 150.6 Hz, 4 F, SF_4_F), 65.1 (d, *J* = 150.6 Hz, 4 F, SF_4_F), 86.6 (quint, *J* = 150.6 Hz, 1 F, SF_4_F), 89.0 (quint, *J* = 150.6 Hz, 1 F, SF_4_F). HRMS-ESI^−^ *m/z* [M − H]^−^ calcd for [C_13_H_8_ClF_10_N_2_OS_2_-H]^−^: 496.9612, found: 496.9624. HPLC (254 nm): t_R_ = 4.22 min (100%).

*1-(2-Chloro-3-(pentafluoro-λ^6^-sulfanyl)phenyl)-3-(4-(pentafluoro-λ^6^-sulfanyl)phenyl)* urea **6**. By following general procedure for the synthesis of aryl isocyanates, 4-(pentafluoro-λ^6^-sulfanyl)aniline (453 mg, 2.06 mmol) in toluene (10 mL) was reacted with triphosgene (306 mg, 1.03 mmol) in the presence of triethylamine (0.29 mL, 2.06 mmol) to afford 4-(pentafluoro-λ^6^-sulfanyl)phenylisocyanate in toluene solution. From this previously-obtained isocyanate and 2-chloro-3-(pentafluoro-λ^6^-sulfanyl)aniline (285 mg, 1.12 mmol) in dichloromethane (5 mL) and following general procedure A, a white solid (920 mg) was obtained. Column chromatography (hexane/ethyl acetate) gave urea **6** (89.6 mg, 16% overall yield) as a white solid, mp 245–246 °C. IR (KBr) ν: 541, 580, 598, 655, 708, 728, 779, 826, 854, 1054, 1102, 1158, 1195, 1225, 1262, 1301, 1412, 1464, 1506, 1546, 1596, 1668, 3134, 3209, 3341 cm^−1^. ^1^H NMR (400 MHz, DMSO-*d*_6_) δ: 7.58 (t, *J* = 8.4 Hz, 1 H, 5-H), 7.66 [m, 2 H, 2′(6′)-H], 7.78–7.87 [complex signal, 3 H, 4-H, 3′(5′)-H], 8.34 (dd, *J* = 8.4 Hz, *J*′ = 1.2 Hz, 1 H, 6-H), 8.80 (broad s, 1 H) and 9.99 (broad s, 1 H) (2 NH). ^13^C NMR (100.6 MHz, CD_3_OD) δ: 119.0 [CH, C2′(6′)], 125.3 (quint, ^3^*J*_CF_ = 5.2 Hz, CH, C4), 126.7 (CH, C6), 128.0–128.1 [complex signal, 1 C and 3 CH, C2, C5 and C3′(5′)], 139.2 (C, C1), 143.7 (C, C1′), 149.1 (m, C, C4′ or C3), 153.0 (m, C, C3 or C4′), 153.9 (C, CO). ^19^F NMR (376.5 MHz, DMSO-*d*_6_) δ: 65.1 (d, *J* = 150.6 Hz, 4 F, SF_4_F), 67.7 (d, *J* = 152.7 Hz, 4 F, SF_4_F), 86.1 (quint, *J* = 152.7 Hz, 1 F, SF_4_F), 89.1 (quint, *J* = 150.6 Hz, 1 F, SF_4_F). Anal. calcd for C_13_H_9_ClF_10_N_2_OS: C 31.30, H 1.82, N 5.62, S 12.86. Found: C 31.30, H 1.82, N 5.31, S 12.61.

*1-(4-Chlorophenyl)-3-(3-(pentafluoro-λ^6^-sulfanyl)phenyl)* urea **7**. From 4-chlorophenylisocyanate (300 mg, 1.95 mmol) and 3-(pentafluoro-λ^6^-sulfanyl)aniline (427 mg, 1.95 mmol) in pyridine (2 mL) and following general procedure B, urea **7** (448 mg, 62% yield) was obtained as a pale white solid by crystallization from methanol, mp (methanol) 204–205 °C (reported 203.5-205 °C [36]). IR (KBr) ν: 644, 686, 698, 752, 781, 794, 804, 842, 872, 926, 940, 1013, 1063, 1091, 1115, 1177, 1237, 1288, 1306, 1401, 1422, 1443, 1485, 1558, 1597, 1660, 3069, 3097, 3195, 3318 cm^−1^. ^1^H NMR (400 MHz, DMSO-*d*_6_) δ: 7.33 [m, 2 H, 3(5)-H], 7.45–7.59 [complex signal, 5 H, 2(6)-H, 4′-H, 5′-H, 6′-H], 8.23 (t, *J* = 4.0 Hz, 1 H, 2′-H), 8.95 (broad s, 1 H) and 9.16 (broad s, 1 H) (2 NH). ^13^C NMR (100.6 MHz, DMSO-*d*_6_) δ: 114.9 (m, CH, C2′), 118.8 (m, CH, C4′), 120.1 [CH, C2(6)], 121.8 (m, CH, C6′), 125.7 (C, C4), 128.5 [CH, C3(5)], 129.6 (CH, C5′), 138.2 (C, C1 or C1′), 140.3 (C, C1′ or C1), 152.3 (C, CO), 153.2 (dquint, ^2^*J*_CF_ = 16.1 Hz, C, C3′). ^19^F NMR (376.5 MHz, DMSO-*d*_6_) δ: 63.6 (d, *J* = 151.0 Hz, 4 F, SF_4_F), 87.7 (quint, *J* = 151.0 Hz, 1 F, SF_4_F). Anal. calcd for C_13_H_10_ClF_5_N_2_OS: C 41.89, H 2.70, N 7.52, S 8.60. Found: C 42.00, H 2.74, N 7.39, S 8.47.

*1-(4-Chlorophenyl)-3-(4-(pentafluoro-λ^6^-sulfanyl)phenyl)* urea **8**. From 4-chlorophenylisocyanate (300 mg, 1.95 mmol) and 4-(pentafluoro-λ^6^-sulfanyl)aniline (427 mg, 1.95 mmol) in pyridine (2 mL) and following general procedure B, urea **8** (580 mg, 80% yield) was obtained as a pale white solid by crystallization from methanol, mp (methanol) 231–232 °C. IR (KBr) ν: 666, 699, 754, 782, 802, 829, 868, 1016, 1090, 1102, 1193, 1216, 1239, 1269, 1300, 1411, 1492, 1504, 1547, 1594, 1610, 1664, 1711, 3086, 3140, 3202, 3324 cm^−1^. ^1^H NMR (400 MHz, DMSO-*d*_6_) δ: 7.34 [m, 2 H, 3(5)-H], 7.50 [m, 2 H, 2(6)-H], 7.64 [d, *J* = 8.0 Hz, 2 H, 2′(6′)-H], 7.80 [m, 2 H, 3′(5′)-H], 8.96 (broad s, 1 H) and 9.22 (broad s, 1 H) (2 NH). ^13^C NMR (100.6 MHz, DMSO-*d*_6_) δ: 117.6 [CH, C2′(6′)], 120.1 [CH, C2(6)], 125.9 (C, C4), 126.8 [quint, ^3^*J*_CF_ = 4.02 Hz, CH, C3′(5′)], 128.7 [CH, C3(5)], 138.2 (C, C1), 143.0 (C, C1′), 146.1 (dquint, ^2^*J*_CF_ = 16.1 Hz, C, C4′), 152.1 (C, CO). ^19^F NMR (376.5 MHz, DMSO-*d*_6_) δ: 65.2 (d, *J* = 150.8 Hz, 4 F, SF_4_F), 89.3 (quint, *J* = 150.8 Hz, 1 F, SF_4_F). Anal. calcd for C_13_H_10_ClF_5_N_2_OS: C 41.89, H 2.70, N 7.52, S 8.60. Found: C 42.03, H 2.89, N 7.41, S 8.55.

*1-(3-(Pentafluoro-λ^6^-sulfanyl)phenyl)-3-(4-(pentafluoro-λ^6^-sulfanyl)phenyl)* urea **9**. By following general procedure for the synthesis of aryl isocyanates, 3-(pentafluoro-λ^6^-sulfanyl)aniline (350 mg, 1.60 mmol) in toluene (5 mL) was reacted with triphosgene (237 mg, 0.80 mmol) in the presence of triethylamine (0.22 mL, 1.60 mmol) to afford 3-(pentafluoro-λ^6^-sulfanyl)phenylisocyanate in toluene solution. From this previously-obtained isocyanate, 4-(pentafluoro-λ^6^-sulfanyl)aniline (246 mg, 1.12 mmol) in anhydrous THF (5 mL) and 2.5 M *n*-butyllithium in hexanes (0.6 mL, 1.46 mmol) and following general procedure C, a brown gum (742 mg) was obtained after quenching any unreacted *n*-butyllithium with methanol (5 mL). Column chromatography (hexane/ ethyl acetate) gave urea **9** (102 mg, 52% overall yield) as a pale white solid, mp 216–217 °C [35]. IR (KBr) ν: 1103, 1196, 1229, 1304, 1410, 1487, 1549, 1597, 1665, 3088, 3134, 3204, 3321 cm^−1^. ^1^H NMR (400 MHz, DMSO-d_6_) δ: 7.48–7.57 (complex signal, 2 H, 4-H, 5-H), 7.58 (dt, *J* = 8.0 Hz, *J*′ = 2.0 Hz, 1 H, 6-H), 7.67 [m, 2 H, 2′(6′)-H], 7.81 [m, 2 H, 3′(5′)-H], 8.24 (m, 1 H, 2-H), 9.27 (broad s, 1 H) and 9.34 (broad s, 1 H) (2 NH). ^13^C NMR (100.6 MHz, DMSO-*d*_6_) δ: 115.3 (m, CH, C2), 117.9 [CH, C2′(6′)], 119.2 (m, CH, C4), 122.1 (CH, C6), 126.8 [m, CH, C3′(5′)], 129.7 (CH, C5), 140.1 (C, C1), 142.8 (C, C1′), 146.3 (dquint, ^2^*J*_CF_ = 16.1 Hz, C, C4′), 152.2 (C, CO), 153.2 (dquint, ^2^*J_CF_* = 16.1 Hz, C, C3). ^19^F NMR (376.5 MHz, DMSO-*d*_6_) δ: 63.6 (d, *J* = 150.4 Hz, 4 F, SF_4_F), 65.1 (d, *J* = 151.0 Hz, 4 F, SF_4_F), 87.6 (quint, *J* = 150.4 Hz, 1 F, SF_4_F), 89.2 (quint, *J* = 151.0, 1 F, SF_4_F). HRMS-ESI^−^ *m/z* [M − H]^−^ calcd for [C_13_H_10_F_10_N_2_OS_2_-H]^−^: 463.0002, found: 463.0017. HPLC (254 nm): t_R_ = 4.04 min (100%).

*1,3-bis(4-(Pentafluoro-λ^6^-sulfanyl)phenyl)* urea **10**. By following general procedure for the synthesis of aryl isocyanates, 4-(pentafluoro-λ^6^-sulfanyl)aniline (259 mg, 1.18 mmol) in toluene (5 mL) was reacted with triphosgene (175 mg, 0.59 mmol) in the presence of triethylamine (0.16 mL, 1.18 mmol) to afford 4-(pentafluoro-λ^6^-sulfanyl)phenylisocyanate in toluene solution. From this previously-obtained isocyanate, 4-(pentafluoro-λ^6^-sulfanyl)aniline (235 mg, 1.07 mmol) in anhydrous THF (5 mL) and 2.5 M *n*-butyllithium in hexanes (0.53 mL, 1.28 mmol) and following general procedure C, an orange gum (618 mg) was obtained after quenching any unreacted *n*-butyllithium with methanol (4 mL). Column chromatography (hexane/ethyl acetate) gave urea **10** (120 mg, 24% overall yield) as a white solid, mp 235 °C (dec) (reported 285.9–287.6 °C, followed by immediate decomposition [37]). IR (ATR) ν: 668, 685, 752, 780, 798, 818, 1013, 1100, 1192, 1212, 1245, 1307, 1317, 1358, 1401, 1411, 1504, 1544, 1593, 1659, 1713, 1974, 2010, 2035, 2066, 2846, 2017, 2958, 3205, 3297, 3323 cm^−1^. ^1^H NMR (400 MHz, DMSO-*d*_6_) δ: 7.66 [d, *J* = 8.8 Hz, 4 H, 2(6)-H], 7.82 [m, 4 H, 3(5)-H], 9.37 (broad s, 2 H, NH). ^13^C NMR (100.6 MHz, DMSO-*d*_6_) δ: 117.8 [CH, C2(6)], 126.8 [m, CH, C3(5)], 142.7 (C, C1), 146.4 (quint, ^2^*J*_CF_ = 16.1 Hz, C, C4), 152.0 (C, CO). ^19^F NMR (376.5 MHz, DMSO-*d*_6_) δ: 65.1 (d, *J* = 150.4 Hz, 4 F, SF_4_F), 89.1 (quint, *J* = 150.4 Hz, 1 F, SF_4_F). Anal. calcd for C_13_H_10_F_10_N_2_OS_2_·0.65C_5_H_12_: C 38.18, H 3.51, N 5.48, S 12.54. Found: C 38.56, H 3.19, N 5.48, S 12.17.

*1,3-bis(3-(Pentafluoro-λ^6^-sulfanyl)phenyl)* urea **11**. By following general procedure for the synthesis of aryl isocyanates, 3-(pentafluoro-λ^6^-sulfanyl)aniline (350 mg, 1.60 mmol) in toluene (5 mL) was reacted with triphosgene (237 mg, 0.80 mmol) in the presence of triethylamine (0.22 mL, 1.60 mmol) to afford 3-(pentafluoro-λ^6^-sulfanyl)phenylisocyanate in toluene solution. From this previously-obtained isocyanate, 3-(pentafluoro-λ^6^-sulfanyl)aniline (351 mg, 1.60 mmol) in anhydrous THF (5 mL) and 2.5 M *n*-butyllithium in hexanes (0.86 mL, 2.08 mmol) and following general procedure C, a beige solid (710 mg) was obtained after quenching any unreacted *n*-butyllithium with methanol (4 mL). Column chromatography (hexane/ ethyl acetate) gave urea **11** (183 mg, 49% overall yield) as a pale white solid. The analytical sample was obtained by crystallization from ethyl acetate, mp (ethyl acetate) 267–268 °C [35]. IR (KBr) ν: 1117, 1242, 1314, 1418, 1485, 1599, 1663, 3102, 3202, 3310 cm^−1^. ^1^H NMR (400 MHz, DMSO-*d*_6_) δ: 7.48–7.57 (complex signal, 4 H, 4-H, 5-H), 7.61 (m, 2 H, 6-H), 8.21 (m, 2 H, 2-H), 9.27 (broad s, 2 H, NH). ^13^C NMR (100.6 MHz, DMSO-*d*_6_) δ: 115.3 (m, CH, C2), 119.2 (m, CH, C4), 122.2 (CH, C6), 129.7 (CH, C5), 140.2 (C, C1), 152.5 (C, CO), 153.2 (quint, ^2^*J*_CF_ = 16.1 Hz, C, C3). ^19^F NMR (376.5 MHz, DMSO-*d*_6_) δ: 63.6 (d, *J* = 151.0 Hz, 4 F, SF_4_F), 87.6 (quint, *J* = 151.0 Hz, 1 F, SF_4_F). HRMS-ESI^−^ *m/z* [M − H]^−^ calcd for [C_13_H_10_F_10_N_2_OS_2_-H]^−^: 463.0002, found: 463.0022. HPLC (254 nm): t_R_ = 4.01 min (100%).

*1,3-bis(4-Chloro-3-(trifluoromethyl)phenyl)* urea **12**. From the commercially available 4-chloro-3-(trifluoromethyl)phenylisocyanate (261 mg, 1.18 mmol) in anhydrous THF (12 mL), 4-chloro-3-(trifluoromethyl)aniline (209 mg, 1.07 mmol) in anhydrous THF (12 mL) and 2.5 M *n*-butyllithium in hexanes (0.49 mL, 1.18 mmol) and following general procedure C, an orange gum (468 mg) was obtained after quenching any unreacted *n*-butyllithium with methanol (4 mL). Column chromatography (hexane/ethyl acetate) gave urea **12** (156 mg, 35% overall yield) as a beige solid, mp 231–232 °C (reported 231–232 °C [1]). IR (ATR) ν: 654, 664, 677, 742, 749, 773, 804, 829, 886, 897, 940, 964, 1031, 1057, 1111, 1129, 1168, 1214, 1227, 1263, 1294, 1318, 1405, 1421, 1478, 1529, 1545, 1594, 1659, 1695, 1713, 1842, 1943, 2144, 2211, 2351, 1852, 2919, 3312, 3328, 3607, 3643, 3669, 3695 cm^−1^. ^1^H NMR (400 MHz, DMSO-*d*_6_) δ: 7.61 (d, *J* = 8.4 Hz, 2 H, 5-H), 7.67 (dd, *J* = 8.4 Hz, *J*′ = 2.0 Hz, 2 H, 6-H), 8.08 (d, *J* = 2.0 Hz, 2 H, 2-H), 9.33 (broad s, 2 H, NH). ^13^C NMR (100.6 MHz, DMSO-*d*_6_) δ: 117.2 (q, ^3^*J*_CF_ = 5.9 Hz, CH, C2), 122.79 (q, ^1^*J*_CF_ = 273.0 Hz, C, CF_3_), 122.80 (q, ^3^*J*_CF_ = 1.9 Hz, C, C4), 123.5 (CH, C6), 126.7 (q, ^2^*J*_CF_ = 30.6 Hz, C, C3), 132.0 (CH, C5), 138.9 (C, C1), 152.3 (C, CO). ^19^F NMR (376.5 MHz, DMSO-*d*_6_) δ: −61.5 (S, 3 F, CF_3_). HRMS-ESI^−^ *m/z* [M − H]^−^ calcd for [C_15_H_8_Cl_2_F_6_N_2_O-H]^−^: 414.9845, found: 414.9839. HPLC (254 nm): t_R_ = 3.52 min (100%).

*1-(4-Chloro-3-(trifluoromethyl)phenyl)-3-(2-chloro-5-(pentafluoro-λ^6^-sulfanyl)phenyl)* urea **13**. By following general procedure for the synthesis of aryl isocyanates, 2-chloro-5-(pentafluoro-λ^6^-sulfanyl)aniline (350 mg, 1.38 mmol) in toluene (5 mL) was reacted with triphosgene (204 mg, 0.69 mmol) in the presence of triethylamine (0.19 mL, 1.38 mmol) to afford 2-chloro-5-(pentafluoro-λ^6^-sulfanyl)phenylisocyanate in toluene solution. From this previously-obtained isocyanate, 4-chloro-3-(trifluoromethyl)aniline (296 mg, 1.51 mmol) in anhydrous THF (12 mL) and 2.5 M *n*-butyllithium in hexanes (0.73 mL, 1.78 mmol) and following general procedure C, a brown oil (767 mg) was obtained after quenching any unreacted *n*-butyllithium with methanol (5 mL). Column chromatography (hexane/ethyl acetate) gave urea **13** (138 mg, 22% overall yield) as a white solid, mp 156–157 °C [35]. IR (ATR) ν: 632, 666, 684, 701, 727, 742, 760, 801, 812, 840, 855, 863, 906, 950, 963, 1034, 1065, 1111, 1126, 1175, 1216, 1229, 1260, 1283, 1301, 1329, 1372, 1408, 1459, 1485, 1513, 1546, 1582, 1592, 1608, 1654, 1695, 1715, 1769, 1905, 1925, 2025, 2179, 2323, 2369, 2851, 2917, 2953, 3276, 3328, 3671, 3733, 3795, 3815 cm^−1^. ^1^H NMR (400 MHz, DMSO-*d*_6_) δ: 7.58 (dd, *J* = 8.8 Hz, *J*′ = 2.4 Hz, 1 H, 4′-H), 7.62–7.71 (complex signal, 2 H, 5-H, 6-H), 7.73 (d, *J* = 8.8 Hz, 1 H, 3′-H), 8.05 (d, *J* = 1.2 Hz, 1 H, 2-H), 8.79 (d, *J* = 2.4 Hz, 1 H, 6′-H), 8.73 (broad s, 1 H) and 10.04 (broad s, 1 H) (2 NH). ^13^C NMR (100.6 MHz, DMSO-*d*_6_) δ: 116.9 (q, ^3^*J*_CF_ = 5.7 Hz, CH, C2), 117.8 (m, CH, C6′), 120.5 (m, CH, C4′), 122.7 (q, ^1^*J*_CF_ = 273.0 Hz, C, CF_3_), 123.1 (m, C, C4), 123.3 (CH, C6), 125.7 (C, C2′), 126.8 (q, ^2^*J*_CF_ = 30.8 Hz, C, C3), 130.0 (CH, C3′), 132.2 (CH, C5), 136.3 (C, C1′), 138.5 (C, C1), 151.4 (m, C, C5′), 152.0 (C, CO). ^19^F NMR (376.5 MHz, DMSO-*d*_6_) δ: –61.5 (S, 3 F, CF_3_), 63.9 (d, *J* = 151.4 Hz, 4 F, SF_4_F), 86.5 (quint, *J* = 151.4 Hz, 1 F, SF_4_F). HRMS-ESI^−^ *m/z* [M − H]^−^ calcd for [C_14_H_8_Cl_2_F_8_N_2_OS-H]^−^: 472.9534, found: 472.9534. HPLC (254 nm): t_R_ = 3.63 min (100%).

*1,3-bis(4-Chloro-3-(pentafluoro-λ^6^-sulfanyl)phenyl)* urea **14**. By following general procedure for the synthesis of aryl isocyanates, 4-chloro-3-(pentafluoro-λ^6^-sulfanyl)aniline (350 mg, 1.37 mmol) in toluene (5 mL) was reacted with triphosgene (204 mg, 0.69 mmol) in the presence of triethylamine (0.20 mL, 1.37 mmol) to afford 4-chloro-3-(pentafluoro-λ^6^-sulfanyl)phenylisocyanate in toluene solution. From this previously-obtained isocyanate, 4-chloro-3-(pentafluoro-λ^6^-sulfanyl)aniline (278 mg, 1.09 mmol) in anhydrous THF (5 mL) and 2.5 M *n*-butyllithium in hexanes (0.60 mL, 1.42 mmol) and following general procedure C, an orange gum (675 mg) was obtained after quenching any unreacted *n*-butyllithium with methanol (4 mL). Column chromatography (hexane/ethyl acetate) gave urea **14** (136 mg, 23% overall yield) as a white solid, mp 237–238 °C [35]. IR (ATR) ν: 1042, 1130, 1227, 1290, 1396, 1477, 1545, 1587, 1645, 1699, 3030, 3138, 3306 cm^−1^. ^1^H NMR (400 MHz, DMSO-*d*_6_) δ: 7.64 (d, *J* = 8.8 Hz, 2 H, 5-H), 7.68 (dd, *J* = 8.8 Hz, *J*′ = 2.0 Hz, 2 H, 6-H), 8.34 (d, *J* = 2.0 Hz, 2 H, 2-H), 9.47 (broad s, 2 H, NH). ^13^C NMR (100.6 MHz, DMSO-*d*_6_) δ: 119.0 (m, CH, C2), 120.4 (C, C4), 123.7 (CH, C6), 133.0 (CH, C5), 138.9 (C, C1), 150.0 (m, C, C3), 152.4 (C, CO). ^19^F NMR (376.5 MHz, DMSO-*d*_6_) δ: 66.9 (d, *J* = 152.5 Hz, 4 F, SF_4_F), 85.8 (quint, *J* = 152.5 Hz, 1 F, SF_4_F). HRMS-ESI^−^ *m/z* [M − H]^−^ calcd for [C_13_H_8_Cl_2_F_10_N_2_OS_2_-H]^−^: 530.9223, found: 530.9236. HPLC (254 nm): t_R_ = 4.35 min (98%).

### 3.2. Bacterial Strains and Growth Conditions

Wild-type *Staphylococcus aureus* CECT 86 (ATCC 12600), *Staphylococcus epidermidis* CECT 231 (ATCC 1798), *Streptococcus mutans* CECT 479 (ATCC 25175), *Enterococcus faecalis* CECT 481 (ATCC 19433), *Escherichia coli* K12 MG1655 CECT 433 (ATCC 700926) and *Pseudomonas aeruginosa* PAO1 CECT 4122 (ATCC 15692) were obtained from the Spanish Type Culture Collection (CECT). *Staphylococcus aureus* MRSA was kindly obtained from Dr Joan Gavaldà laboratory. All strains were routinely cultivated in TSB (tryptic soy broth) or LB (Luria-Bertani) medium (Scharlab) at 37 °C.

### 3.3. Antibacterial Susceptibility Testing

Bacterial strains were tested in the presence of different compounds; each strain was grown in TSB medium to OD_550_ ≈ 0.1 and plated in a microtiter plate (Corning 3596 Polystyrene Flat Bottom 96 Well, Corning, NY, USA) with different compound concentrations according to the Clinical Laboratory Standards Institute (CLSI) guidelines, as previously described [41]. The plate was incubated at 37 °C and 150 rpm and growth curves were monitored for 8 h taking the absorbance (OD_550_ nm) every 15 min in an SPARK Multimode microplate reader (Tecan, Männedorf, Switzerland). The minimal inhibitory concentration 50% (MIC_50_) was defined as the compound concentration that reduces bacterial growth, determined as the OD_550_, by 50%.

### 3.4. Antibacterial Effect of Compounds on Cleaning a Surface

Sterile cover glasses (2.4 cm × 5 cm) (Duran) were placed into a petri dish with a solution of peptone water (Sigma-Aldrich, St. Louis, MO, USA) inoculated with *S. aureus* at an OD_550_ ≈ 0.1 and incubated at 20 °C without shaking. After 16 h, the cover glasses were washed with phosphate buffered saline (PBS), and the different compounds were added at a concentration of 3 × MIC. After 1 h of incubation, the covers were placed directly on agar plates to quantify viable cells (cfu/mL). The viable counts at control experiment were 550 ± 114 cfu.

### 3.5. Antibacterial Effect of Compounds on Biofilms Growing on Catheters

Sterile pieces of catheter (1 cm width and 2 mm diameter) were incubated in 10 ml tubes with 1 mL TSB with 0.2% glucose inoculated with *S. aureus* at OD_550_ ≈ 0.1. After three days without shaking at 37 °C, all tubes were washed three times with PBS to remove non-adhered bacteria (planktonic) and, after, the different compounds were added at a 1 × MIC concentration. After overnight incubation, tubes were washed three times with PBS and 1 mL PBS + 0.05% TWEEN solution was added to each tube. All tubes were placed in an ultrasonic bath (VWR) for 5 min and then vortexed for 30 s to remove adhered bacteria (growing in biofilm). The control group contained media only. Biofilm viable cells (cfu) were determined by plating serial dilutions on agar plates. The viable counts at control experiment were 3.8 × 10^6^ ± 1.2 × 10^6^ cfu/mL.

### 3.6. Fluorescent Microscopy Viability Test Analysis

*S. aureus* was grown in TSB medium at 37 °C and 150 rpm to reach an OD_550_ of 0.2, where different compounds were added at 1 × MIC. After 4 h in shaking conditions, cells (1 mL) were centrifuged and stained using the LIVE/DEAD BacLight Bacterial Viability kit (Thermo Fisher Scientific). After 30 min at room temperature under dark conditions, cells were washed with PBS to remove nonspecific stain. Fluorescent bacteria were visualized by a Nikon inverted fluorescent microscope ECLISPSE Ti-S/L100 (Nikon) coupled with a DS-Qi2 Nikon camera (Nikon). To access membrane integrity, cells were also stained with 10 µg/mL of *N*-(3-triethylammoniumpropyl)-4-(6-(4-(diethylamino)phenyl)hexatrienyl)pyridinium dibromide (FM^®^ 4-64, Thermo Fisher Scientific). The dye was added after a 10 min treatment with the compounds at 1 × MIC on *S. aureus* grown in TSB medium at 37 °C and 150 rpm until an OD_550_ ≈ 1.

### 3.7. Spontaneous Mutation Frequency to Resistance

A culture of *S. aureus* (10^10^ cfu/mL) was plated in TSA agar plates containing different compounds at 10 µg/mL. The inoculum viable cells were determined by cfu counting. The spontaneous compound-resistant mutation frequency was calculated by dividing the number of resistant colonies by the total viable cells.

### 3.8. Mammalian Cytotoxicity Determination

J-774A.1 murine macrophages cells (DSMZ ACC 170) were seeded in a microtiter plate (2 × 10^4^ cells per well) (Corning 3596 Polystyrene Flat Bottom 96 Well, Corning), infected with the different compounds at different concentrations, and diluted in complete medium (Gibco) without antibiotics. After 24 h at 37 °C, the supernatants were removed and a 10% of MTT solution (3-[4,5-dimethylthiazol-2-yl]-2,5-diphenyltetrazolium bromide, Sigma-Aldrich) was added to determine cell viability. The formazan produced after 3 h was dissolved with acidic isopropanol, and absorbance was measured at 550 nm with a SPARK Multimode microplate reader (Tecan). CC_50_ was calculated with GraphPad Prism 6.00 (GraphPad Software) as the concentration of compound that reduces the cell viability by 50%.

## 4. Patents

A PCT patent application has been filed. See PCT WO2018/010856A1 (priority data 13 July 2016).

## 5. Conclusions

Thirteen new diarylureas featuring the scarcely-explored pentafluorosulfanyl group have been synthesized as analogs of TCC, a widely-used antimicrobial agent that has recently been banned by the FDA. Overall, the novel derivatives showed similar potency and comparable or broader spectrum of activity than TCC. Compound **10**, with higher potency, a broader spectrum of activity, and higher selectivity index emerged as the most promising compound. A bactericidal mode of action for this family of ureas was suggested by preliminary experiments. It is worthy of note that some of these new molecules removed preexisting *S. aureus* biofilms, which is important in food industry as well as in hospital settings, and displayed a lower spontaneous mutation frequency in *S. aureus* than TCC.

## Figures and Tables

**Table 1:** MIC_50_, cytotoxicity and selectivity index (SI) of compounds **1**–**14**.*^a^*

Compound	MIC_50_ (µg/mL) *^b^*^,^*^c^*					CC_50_ (µg/mL) *^d^*
	*S. aureus*	*S. aureus MRSA*	*S. epidermis*	*S. mutants*	*E. faecalis*	
**TCC**	0.5 (29)	0.5 (29)	0.5 (29)	NA *^e^*	NA	14.5
**TFC**	0.5 (27.4)	0.5 (27.4)	0.5 (27.4)	NA	>20	13.7
**1**	1 (4.8)	1 (4.8)	1 (4.8)	5	1 (4.8)	4.8
**2**	1 (4.8)	1 (4.8)	1 (4.8)	5	1 (4.8)	4.8
**3**	0.5 (110)	0.5 (110)	1 (55)	NA	NA	55
**4**	0.5 (56)	0.5 (56)	0.5 (56)	NA	5	28
**5**	0.5 (10.2)	0.5 (10.2)	0.5 (10.2)	0.5 (10.2)	0.5 (10.2)	5.6
**6**	0.5 (8.4)	0.5 (8.4)	0.5 (8.4)	0.5 (8.4)	0.5 (8.4)	4.2
**7**	0.5 (26.2)	0.5 (26.2)	0.5 (26.2)	>20	5	13.1
**8**	0.5 (26)	0.5 (26)	0.5 (26)	NA	0.5 (26)	13
**9**	0.5 (25)	0.5 (25)	0.5 (25)	0.5 (25)	0.5 (25)	12.5
**10**	0.05 (412)	0.05 (412)	0.5 (41.2)	0.5 (41.2)	0.5 (41.2)	20.6
**11**	0.5 (67)	0.5 (67)	0.5 (67)	0.5 (67)	>10	33.5
**12**	0.5 (67.6)	0.3 (112.7)	0.5 (67.6)	2 (16.9)	2 (16.9)	33.8
**13**	0.5 (49.2)	0.5 (49.2)	0.5 (49.2)	0.5 (49.2)	0.5 (49.2)	24.6
**14**	0.5 (20)	0.5 (20)	0.5 (20)	0.5 (20)	0.5 (20)	10

*^a^* For each tested compound, MIC_50_ values are represented in the first row, whereas SI values are indicated in brackets. *^b^* MIC_50_ values were determined against the following Gram-positive bacterial strains: *Staphylococcus aureus* (ATCC 12600); *Staphylococcus aureus methyciline resistant*; *Staphylococcus epidermis* (ATCC 1798); *Streptococcus mutants* (ATCC 25175); *Enterococcus faecalis* (ATCC 19433). *^c^* All the compounds were inactive against two Gram-negative bacterial strains: *Escherichia coli* (ATCC 700926) and *Pseudomonas aeruginosa* (ATCC 15692). *^d^* CC_50_ values were determined against murine macrophages cells (J-774A.1). *^e^* NA, no antibacterial activity detected (MIC_50_ > 100 µg/mL).

**Table 2:** Spontaneous mutation rates in *S. aureus* to different diarylureas.

Compound (10 µg/ mL)	Frequency of Mutation
**TCC**	4 × 10^−9^
**3**	0
**9**	0
**10**	0
**11**	5 × 10^−1^
**12**	5 × 10^−10^
**13**	0

## Supplementary Materials

The following are available online. copies of the ^1^H, ^13^C and ^19^F NMR spectra of the new compounds.
