# Supplementary material for: Pentafluorosulfanyl-containing Triclocarban Analogs with Potent Antimicrobial Activity
Source: Molecules. 2018 Nov 2;23(11):2853. doi: 10.3390/molecules23112853 (PMC6278391; doi:10.3390/molecules23112853)
Supplement: Supplementary file 1 [file molecules-23-02853-s001.pdf]

SUPPLEMENTARY MATERIAL FOR

Pentafluorosulfanyl-containing triclocarban analogs with potent antimicrobial activity

Eugènia Pujol[a], Núria Blanco-Cabra[b], Esther Julián[c], Rosana Leiva[a], Eduard Torrents[b] and Santiago Vázquez[a]

[a]Laboratori de Química Farmacèutica (Unitat Associada al CSIC), Facultat de Farmàcia i Ciències de l'Alimentació, and Institute of Biomedicine (IBUB), Universitat de Barcelona, Av. Joan XXIII 27-31, 08028 Barcelona, Spain

[b]Bacterial Infections and Antimicrobial Therapies, Institute for Bioengineering of Catalonia (IBEC), The Barcelona Institute of Science and Technology, Baldiri Reixac 15-21, 08028 Barcelona, Spain

[c]Departament de Genètica i de Microbiologia, Facultat de Biociències, Universitat Autònoma de Barcelona, 08193 Bellaterra, Spain

Table of contents

Figure S1	Page S3
Scheme S1	Page S4
Scheme S2	Page S4
Scheme S3	Page S5
Scheme S4	Page S5
^{1}H, ^{13}C and ^{19}F NMR spectra of new compounds	Page S6

Figure S1. Pentafluorosulfanyl ureas 1-14

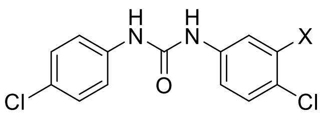

TCC, X = Cl
Cloflucarban, X = CF_3

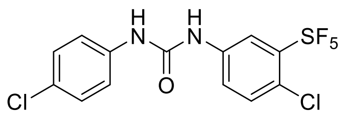

1

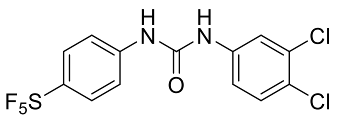

2

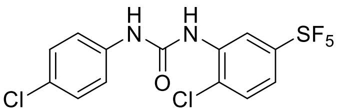

3

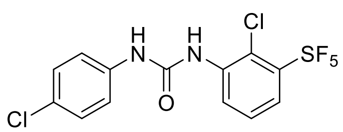

4

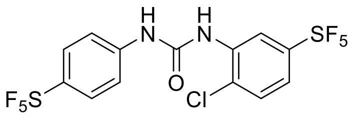

5

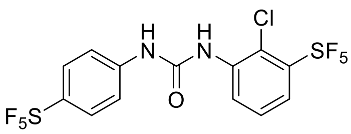

6

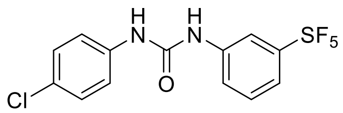

7

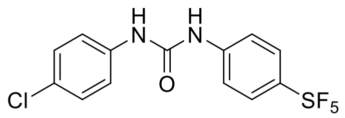

8

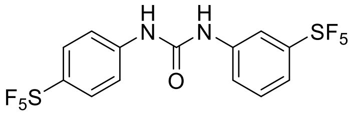

9

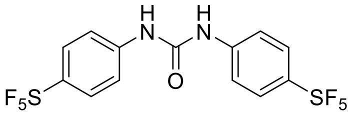

10

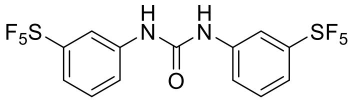

11

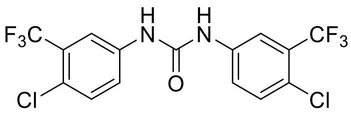

12

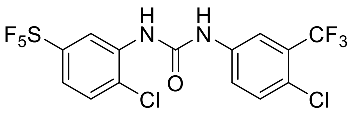

13

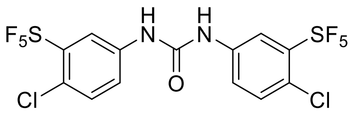

14

Scheme S1. General procedure for the synthesis of intermediate isocyanates

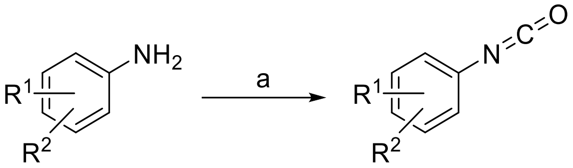

Reagents and conditions: (a) triphosgene, toluene, Et_3N, 70 ºC, 2 h.

Schemes S2-S4. General synthetic schemes of the final compounds 1-14

Scheme S2. Synthesis of compounds 2-6

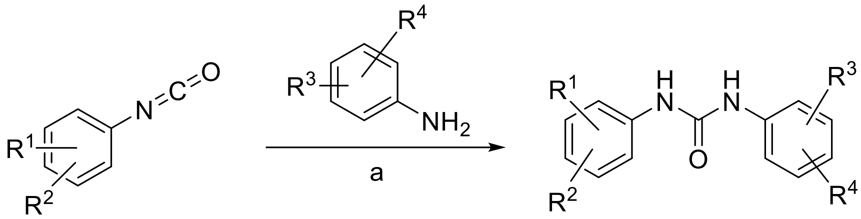

Comp. 2-6

Reagents and conditions: (a) DCM, overnight.

Compound	R^1	R^2	R^3	R^4	Overall yield (%)
**2**	3-Cl	4-Cl	4-SF_5	H	30
**3**	5-SF_5	2-Cl	4-Cl	H	47
**4**	3-SF_5	2-Cl	4-Cl	H	8
**5**	5-SF_5	2-Cl	4-SF_5	H	6
**6**	4-SF_5	H	3-SF_5	2-Cl	9

Scheme S3. Synthesis of compounds 1, 7-8

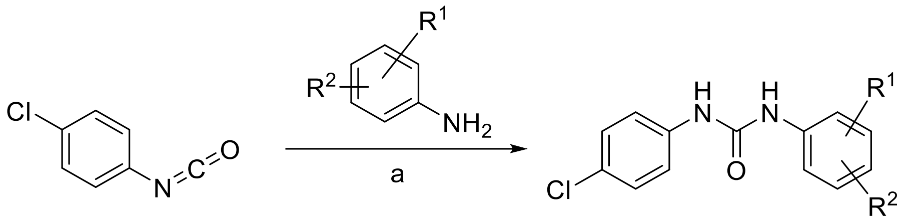

Comp. 1, 7-8

Reagents and conditions: (a) pyridine, 1 h.

Compound	R^1	R^2	Overall yield (%)
**1**	3-SF_5	4-Cl	17
**7**	3-SF_5	H	62
**8**	4-SF_5	H	80

Scheme S4. Synthesis of compounds 9-14

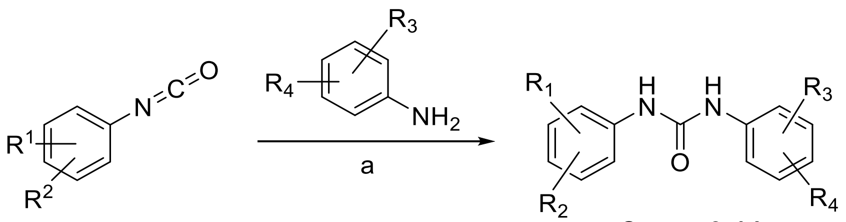

Comp. 9-14

Reagents and conditions: (a) *n*-BuLi, anh. TFH, overnight.

Compound	R^1	R^2	R^3	R^4	Overall yield (%)
**9**	3-SF_5	H	4-SF_5	H	20
**10**	4-SF_5	H	4-SF_5	H	22
**11**	3-SF_5	H	3-SF_5	H	49
**12**	3-CF_3	4-Cl	3-CF_3	4-Cl	35
**13**	5-SF_5	2-Cl	3-CF_3	4-Cl	22
**14**	3-SF_5	4-Cl	3-SF_5	4-Cl	23

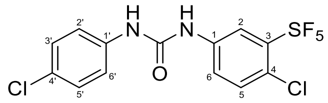

H
N
H
N
SF5
Cl
Cl
O
2'
3'
1'
4'
5'
6'
1
2
3
4
5
6

1

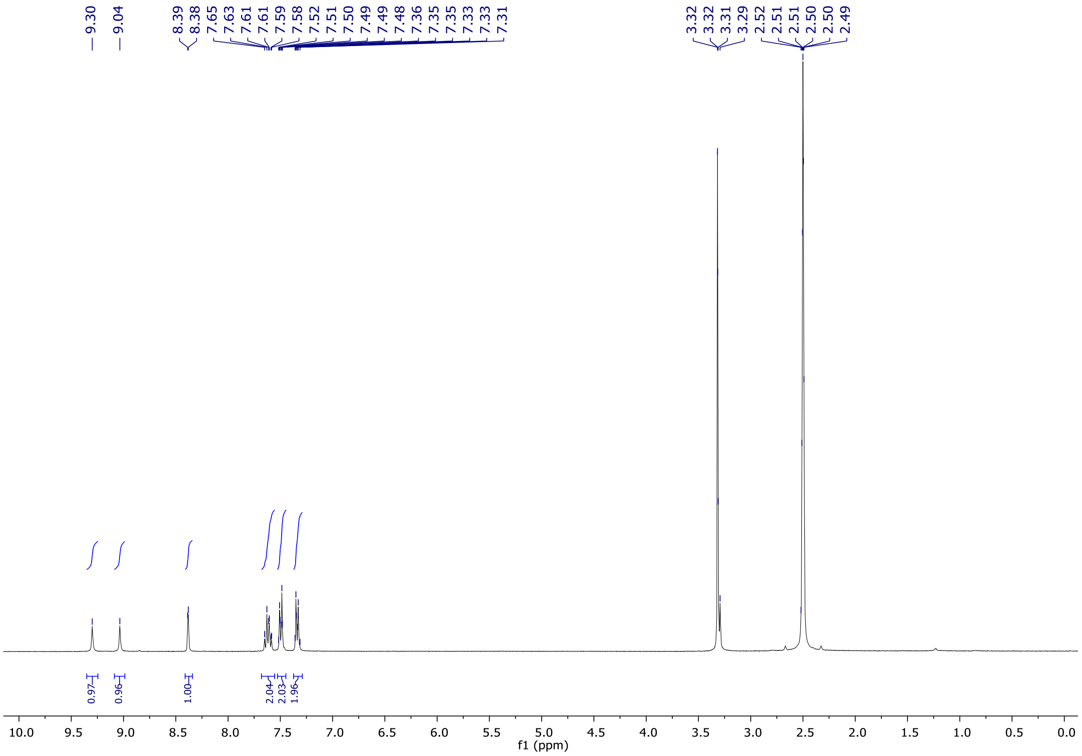

9.30
9.04
8.39
8.38
7.65
7.63
7.61
7.61
7.59
7.58
7.52
7.51
7.50
7.49
7.49
7.48
7.36
7.35
7.35
7.33
7.33
7.31
3.32
3.32
3.31
3.29
2.52
2.51
2.51
2.50
2.50
2.49
0.97
0.96
1.00
2.04
2.03
1.96
f1 (ppm)

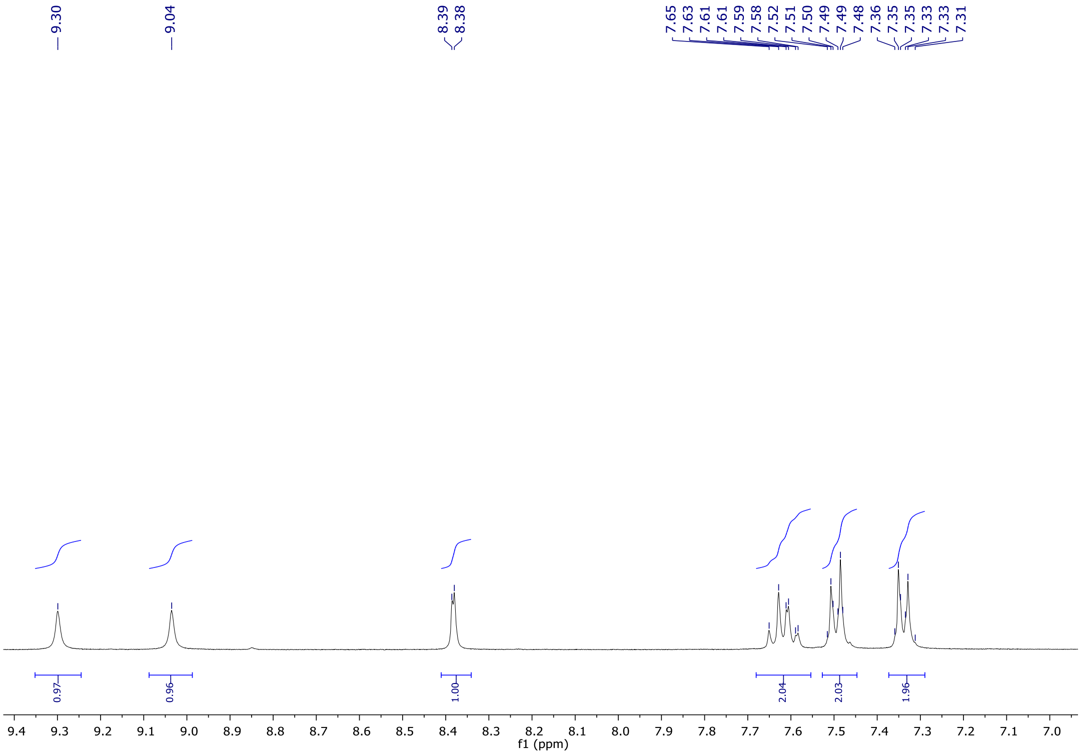

9.30
9.04
8.39
8.38
7.65
7.63
7.61
7.61
7.59
7.58
7.52
7.51
7.50
7.49
7.49
7.48
7.36
7.35
7.35
7.33
7.33
7.31
0.97
0.96
1.00
2.04
2.03
1.96
f1 (ppm)

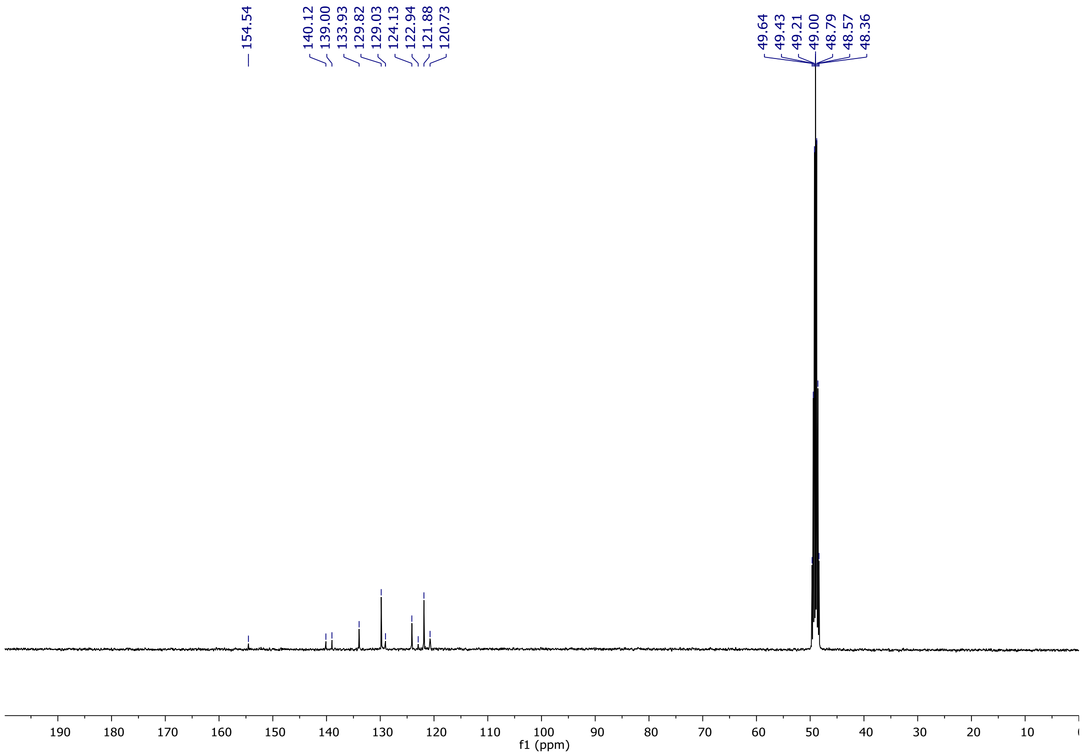
154.54
140.12
139.00
133.93
129.82
129.03
124.13
122.94
121.88
120.73
49.64
49.43
49.21
49.00
48.79
48.57
48.36
190 180 170 160 150 140 130 120 110 100 90 80 70 60 50 40 30 20 10
f1 (ppm)

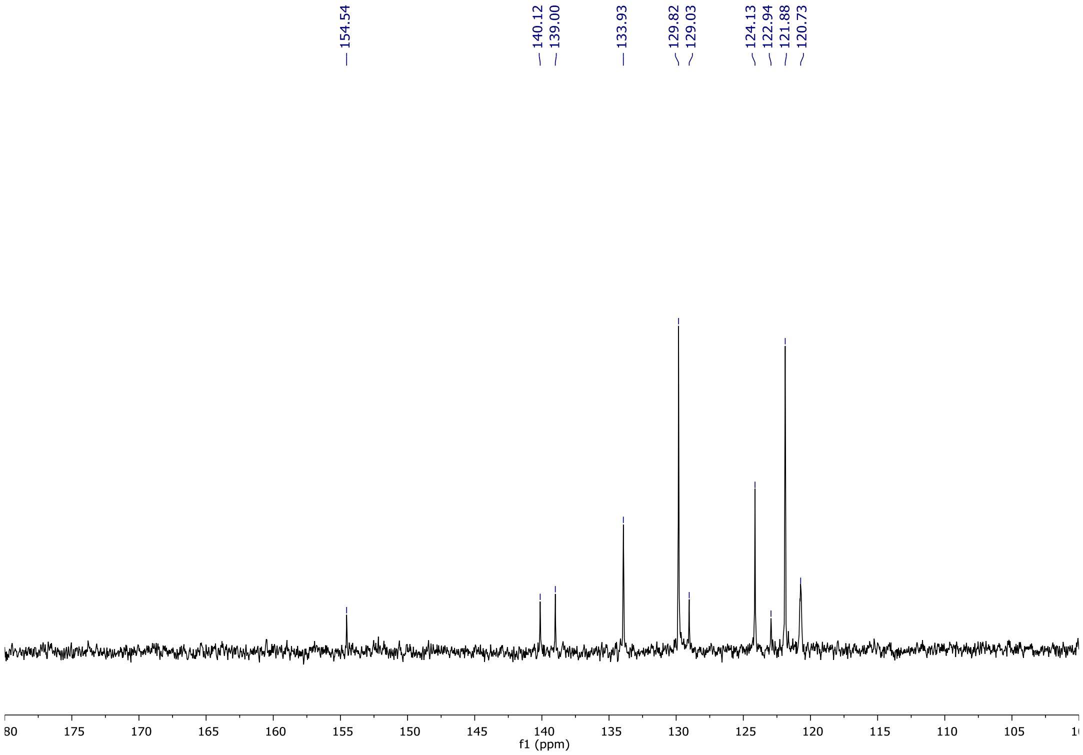
154.54
140.12
139.00
133.93
129.82
129.03
124.13
122.94
121.88
120.73
175 170 165 160 155 150 145 140 135 130 125 120 115 110 105
f1 (ppm)

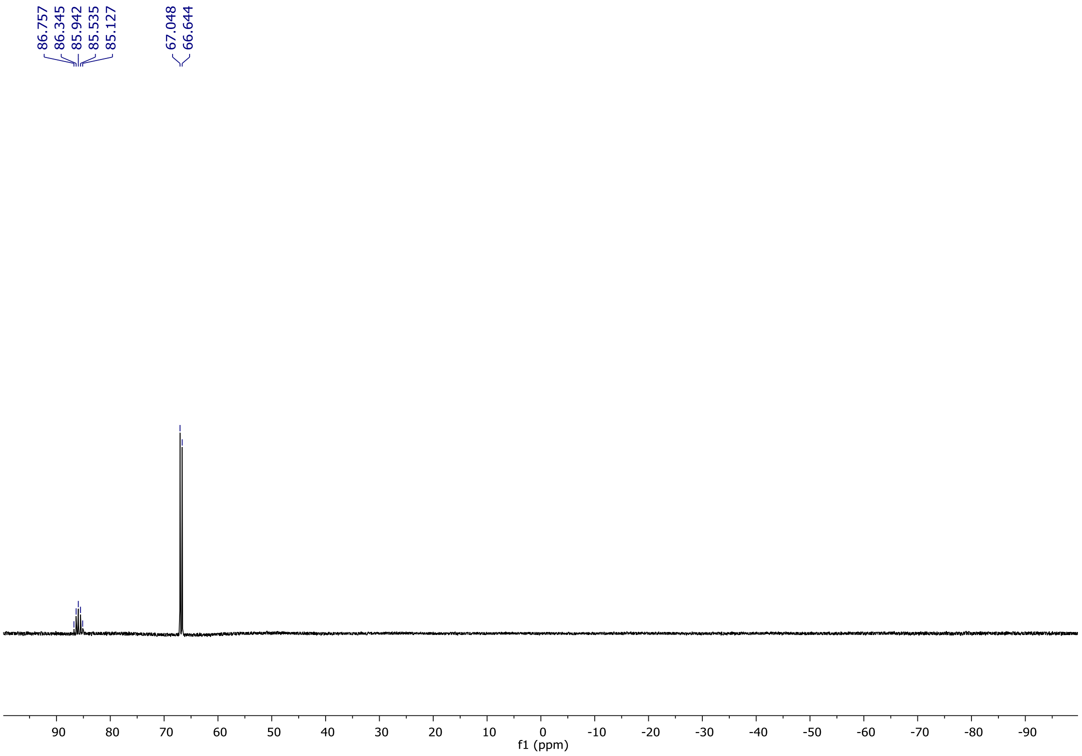
86.757
86.345
85.942
85.535
85.127
67.048
66.644
90 80 70 60 50 40 30 20 10 0 -10 -20 -30 -40 -50 -60 -70 -80 -90
f1 (ppm)

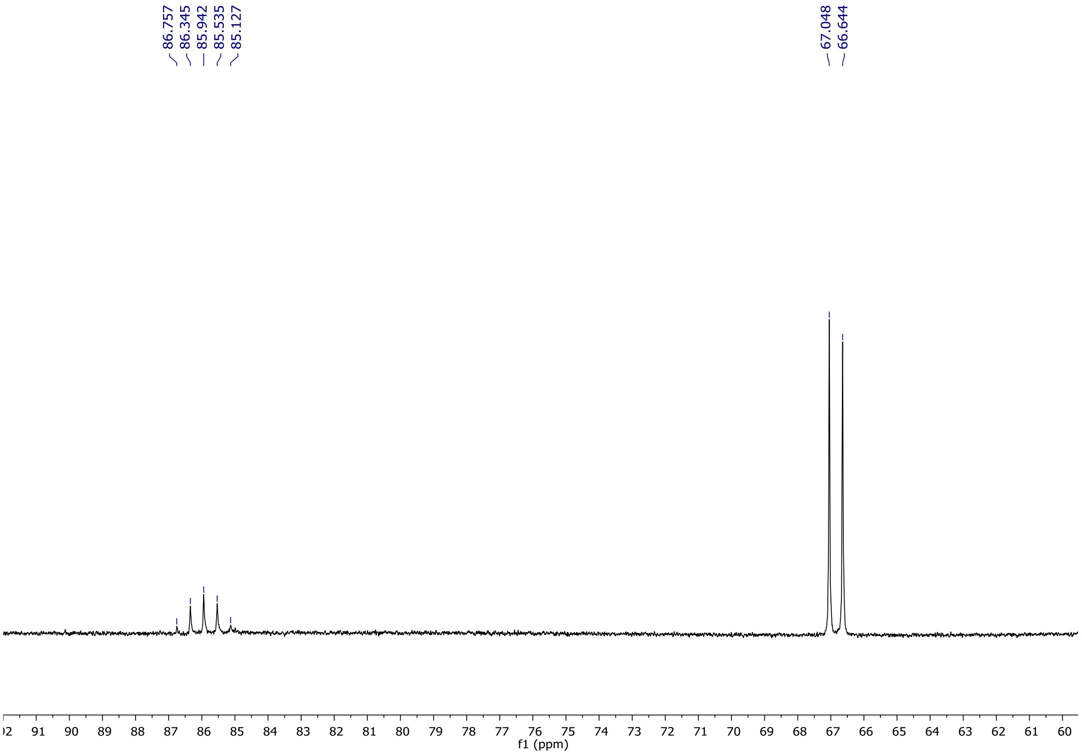
86.757
86.345
85.942
85.535
85.127
67.048
66.644
91 90 89 88 87 86 85 84 83 82 81 80 79 78 77 76 75 74 73 72 71 70 69 68 67 66 65 64 63 62 61 60
f1 (ppm)

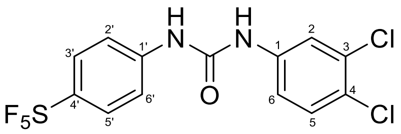

H
H
N
N
O
Cl
Cl
F5S
1'
2'
3'
4'
5'
6'
1
2
3
4
5
6

2

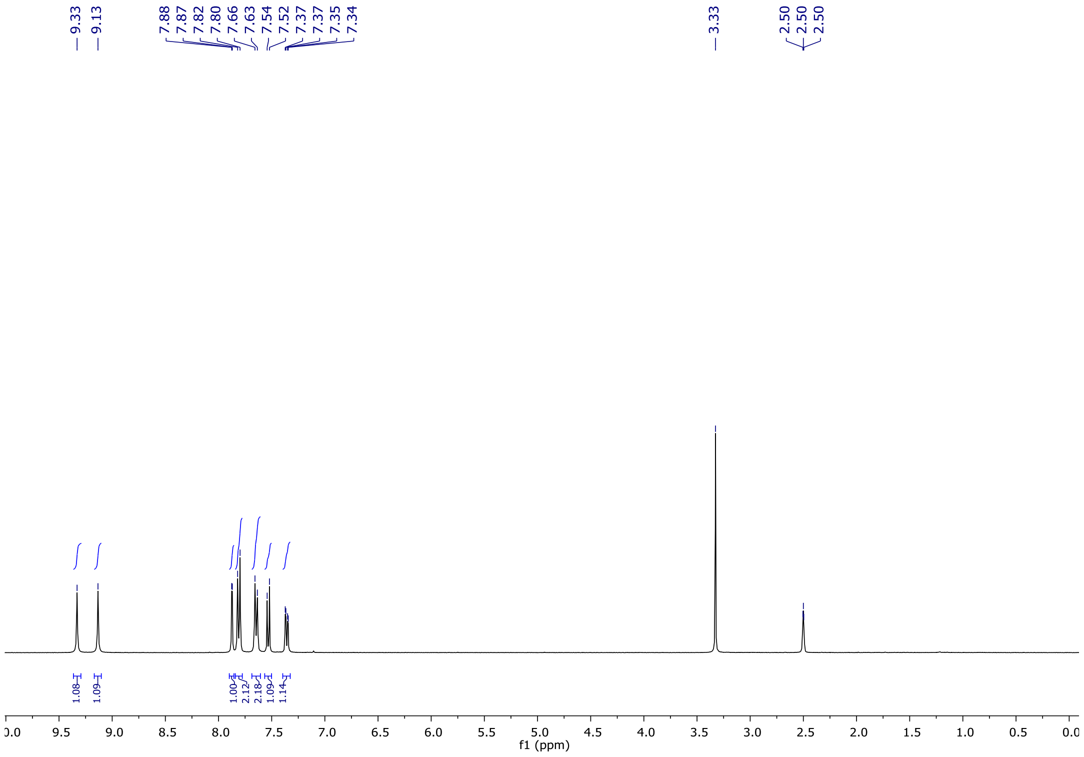

9.33
9.13
7.88
7.87
7.82
7.80
7.66
7.63
7.54
7.52
7.37
7.37
7.35
7.34
3.33
2.50
2.50
2.50
1.08
1.09
1.00
2.12
2.18
1.09
1.14
10.0 9.5 9.0 8.5 8.0 7.5 7.0 6.5 6.0 5.5 5.0 4.5 4.0 3.5 3.0 2.5 2.0 1.5 1.0 0.5 0.0
f1 (ppm)

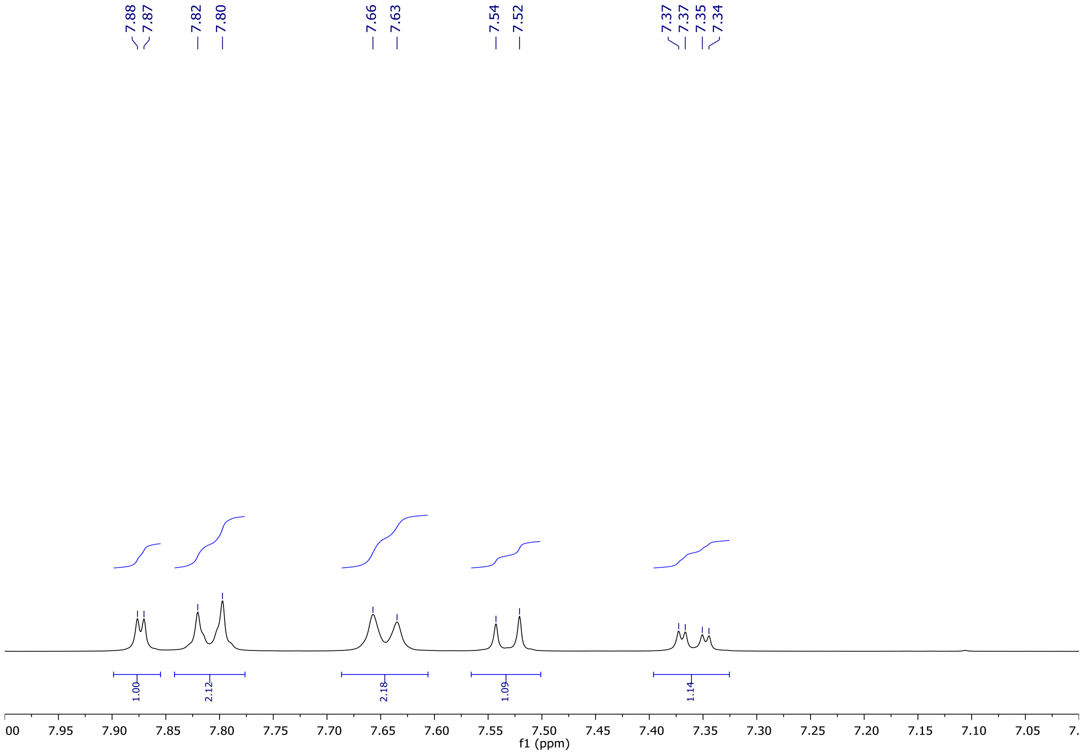

7.88
7.87
7.82
7.80
7.66
7.63
7.54
7.52
7.37
7.37
7.35
7.34
1.00
2.12
2.18
1.09
1.14
8.00 7.95 7.90 7.85 7.80 7.75 7.70 7.65 7.60 7.55 7.50 7.45 7.40 7.35 7.30 7.25 7.20 7.15 7.10 7.05 7.00
f1 (ppm)

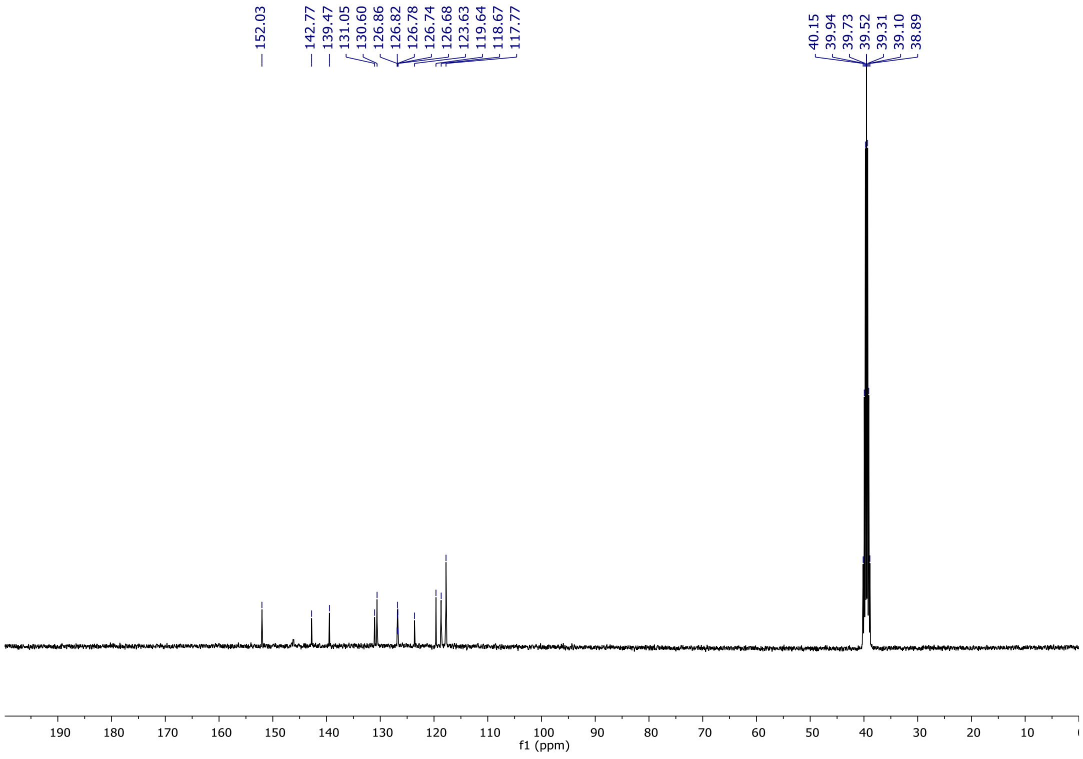
152.03
142.77
139.47
131.05
130.60
126.86
126.82
126.78
126.74
126.68
123.63
119.64
118.67
117.77
40.15
39.94
39.73
39.52
39.31
39.10
38.89
f1 (ppm)

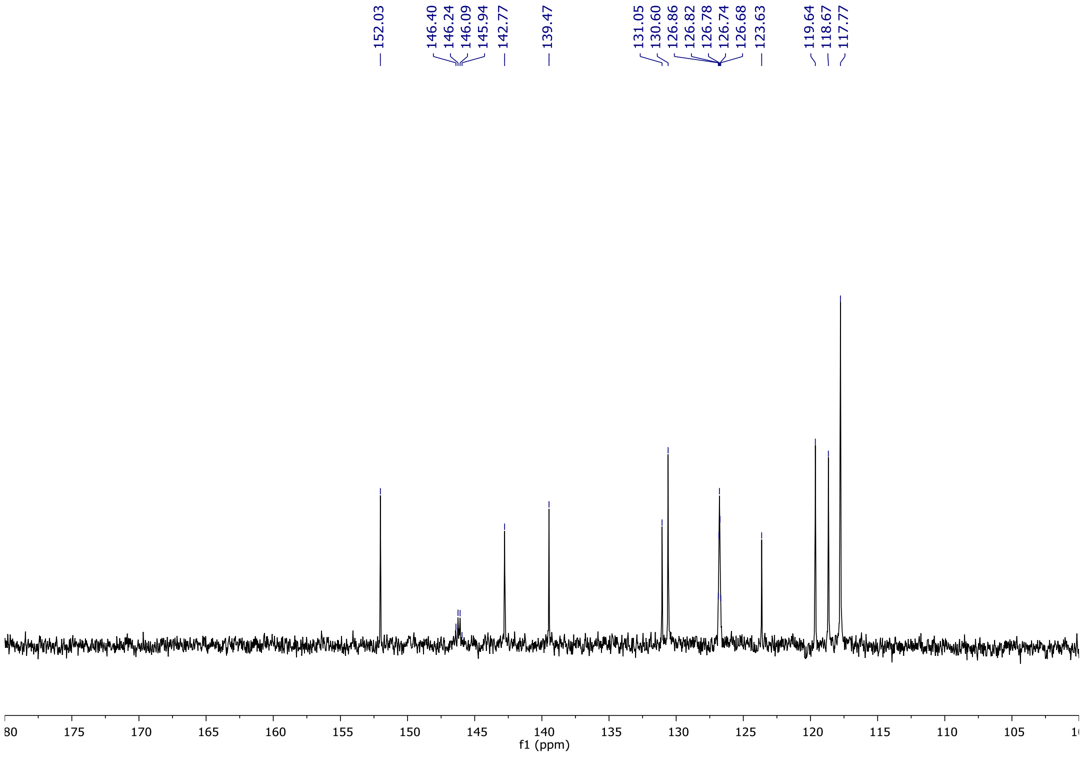
152.03
146.40
146.24
146.09
145.94
142.77
139.47
131.05
130.60
126.86
126.82
126.78
126.74
126.68
123.63
119.64
118.67
117.77
f1 (ppm)

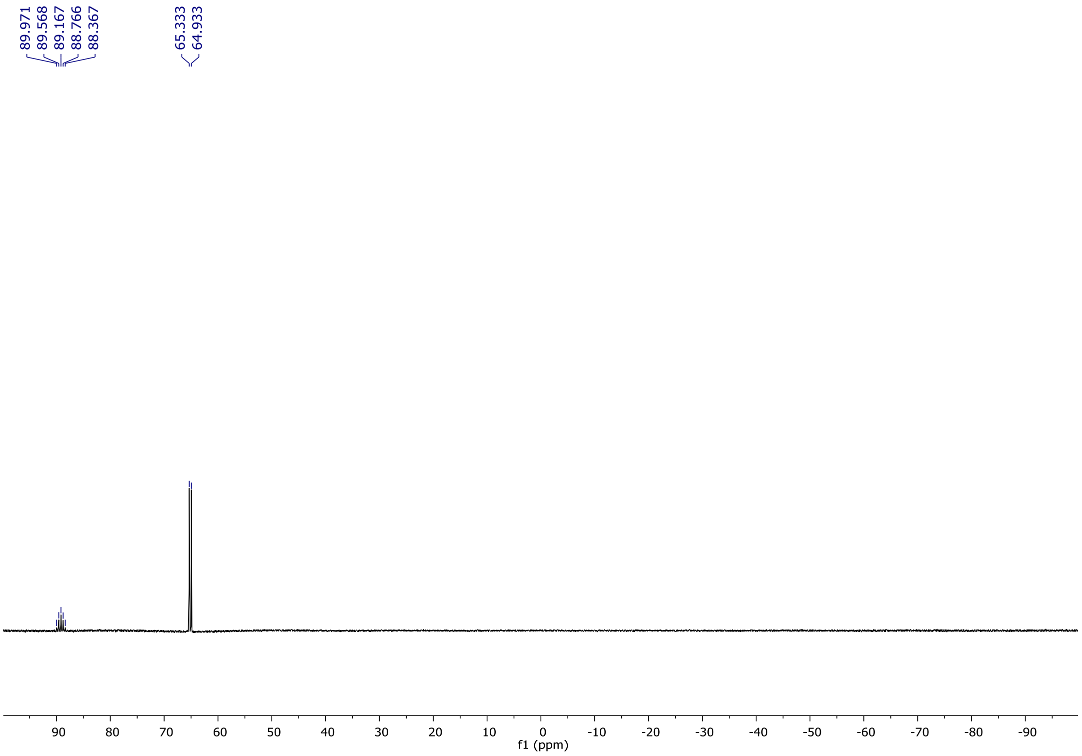

89.971
89.568
89.167
88.766
88.367
65.333
64.933
90 80 70 60 50 40 30 20 10 0 -10 -20 -30 -40 -50 -60 -70 -80 -90
f1 (ppm)

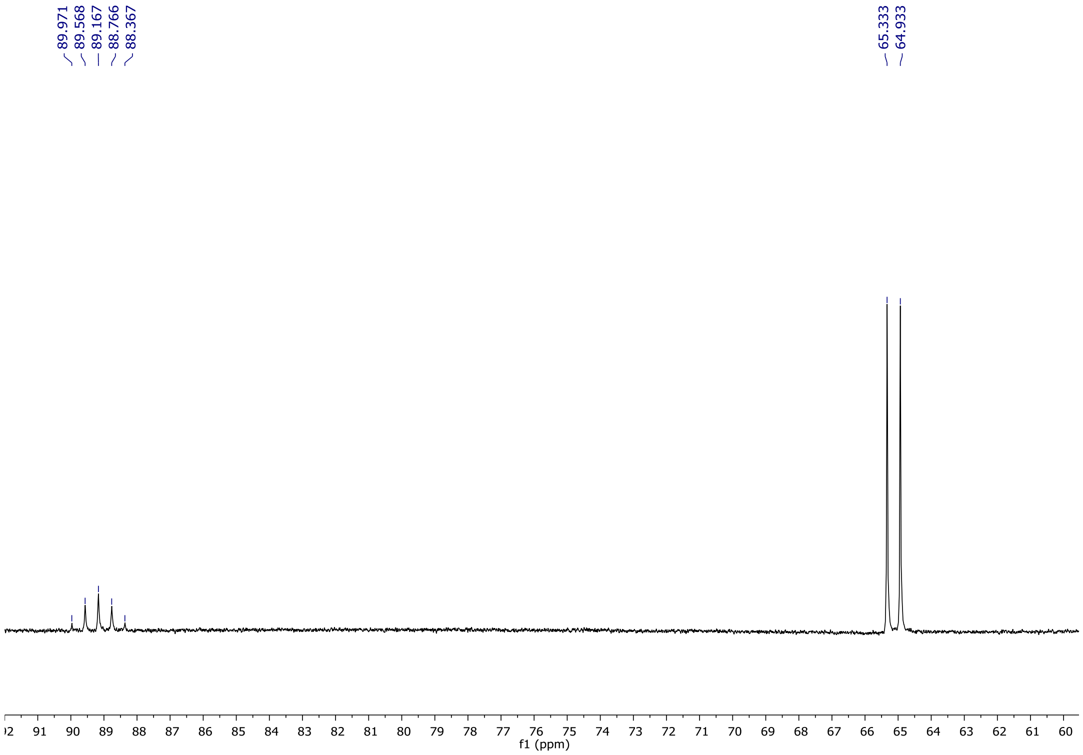

89.971
89.568
89.167
88.766
88.367
65.333
64.933
91 90 89 88 87 86 85 84 83 82 81 80 79 78 77 76 75 74 73 72 71 70 69 68 67 66 65 64 63 62 61 60
f1 (ppm)

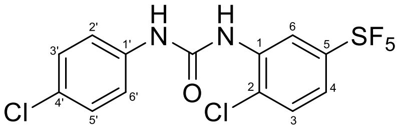

3

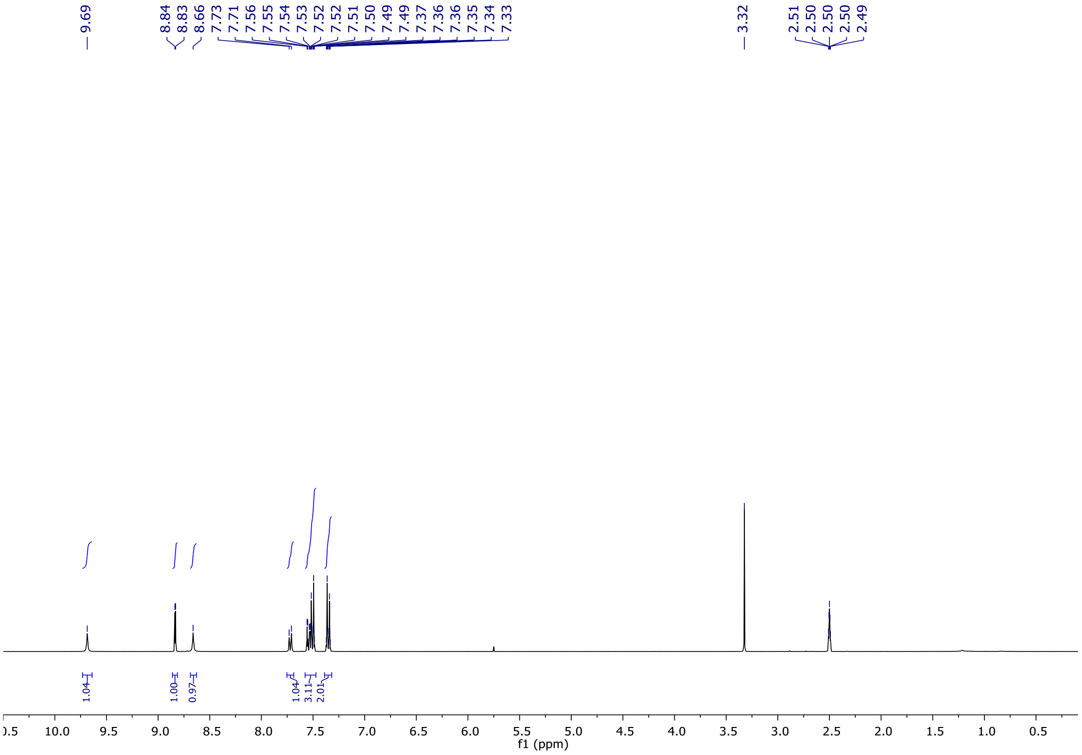

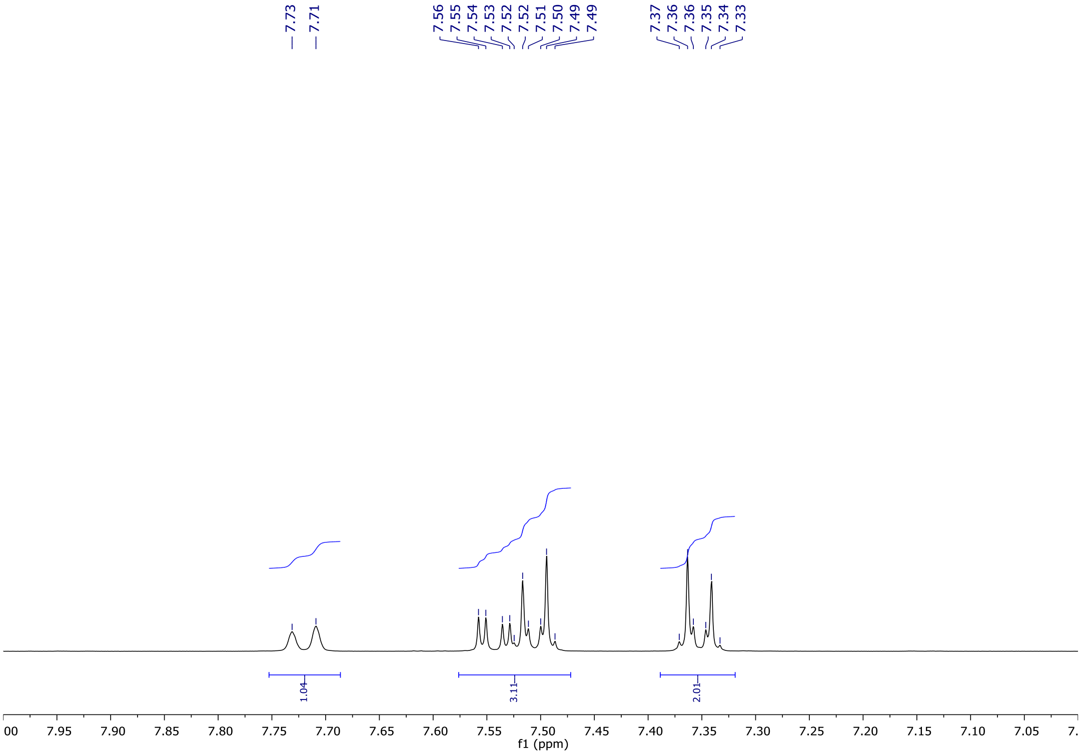

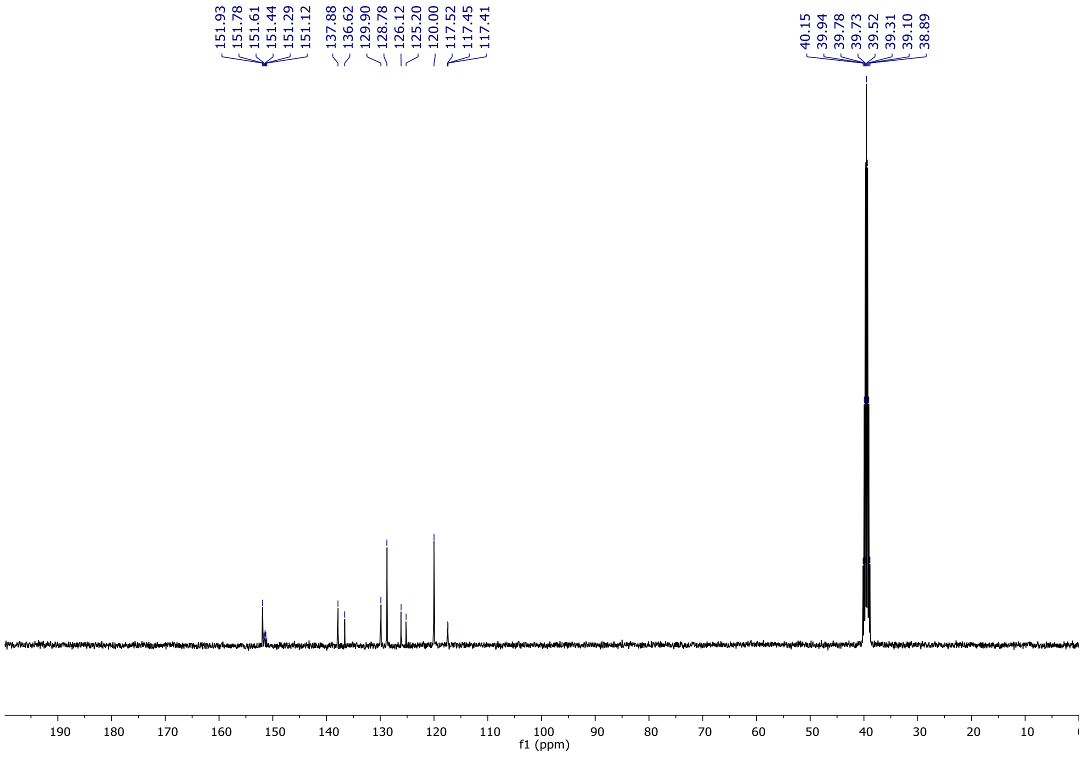
151.93
151.78
151.61
151.44
151.29
151.12
137.88
136.62
129.90
128.78
126.12
125.20
120.00
117.52
117.45
117.41
40.15
39.94
39.78
39.73
39.52
39.31
39.10
38.89
190 180 170 160 150 140 130 120 110 100 90 80 70 60 50 40 30 20 10
f1 (ppm)

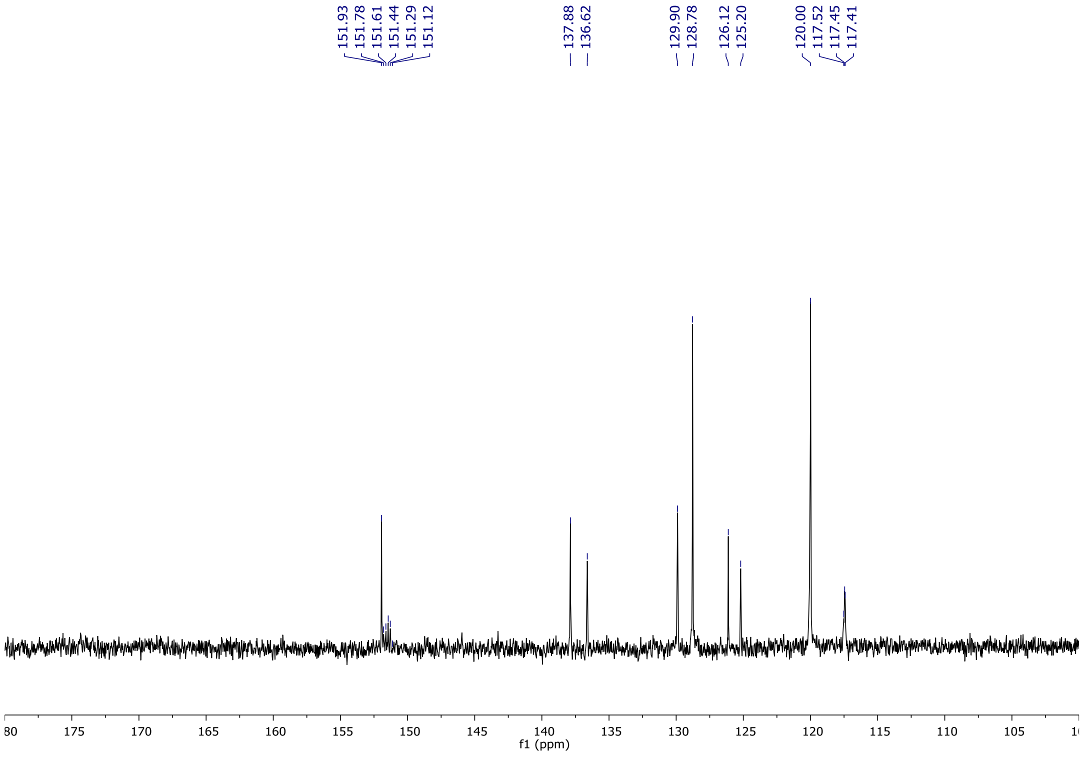
151.93
151.78
151.61
151.44
151.29
151.12
137.88
136.62
129.90
128.78
126.12
125.20
120.00
117.52
117.45
117.41
175 170 165 160 155 150 145 140 135 130 125 120 115 110 105
f1 (ppm)

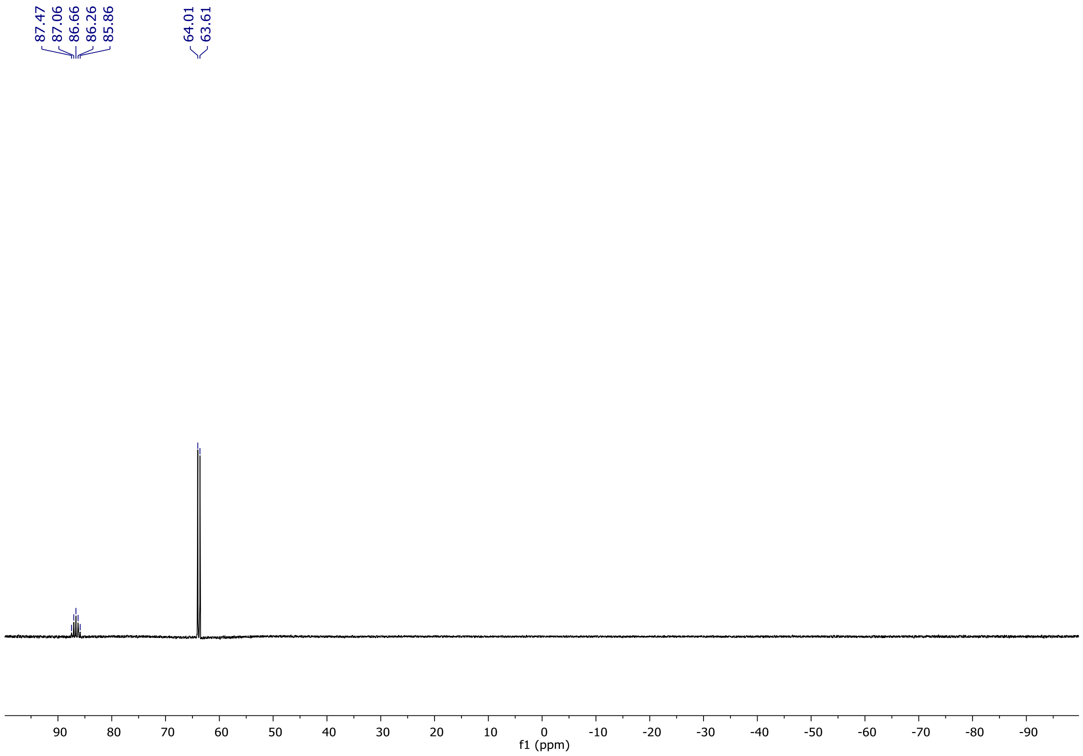

87.47
87.06
86.66
86.26
85.86
64.01
63.61
90 80 70 60 50 40 30 20 10 0 -10 -20 -30 -40 -50 -60 -70 -80 -90
f1 (ppm)

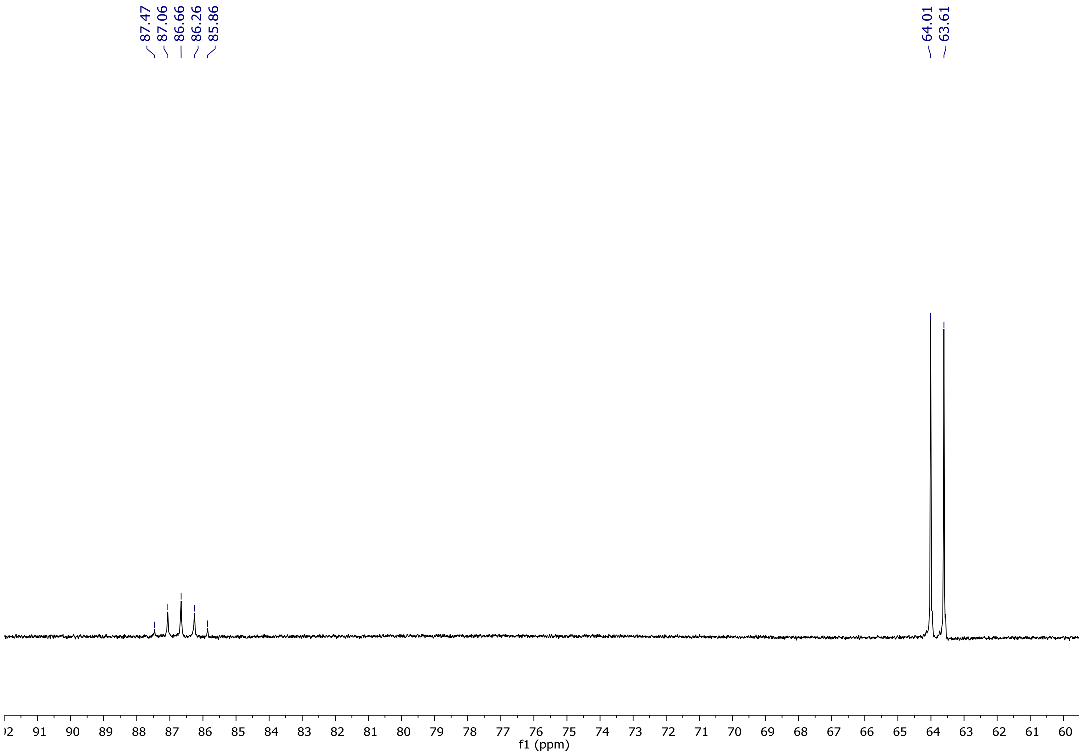

87.47
87.06
86.66
86.26
85.86
64.01
63.61
91 90 89 88 87 86 85 84 83 82 81 80 79 78 77 76 75 74 73 72 71 70 69 68 67 66 65 64 63 62 61 60
f1 (ppm)

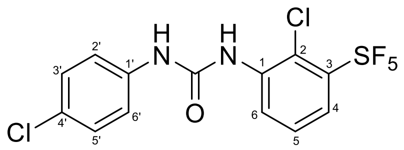

Cl
H
N
H
N
Cl
SF5
O
2'
3'
1'
4'
6'
5'
1
2
3
4
5
6

4

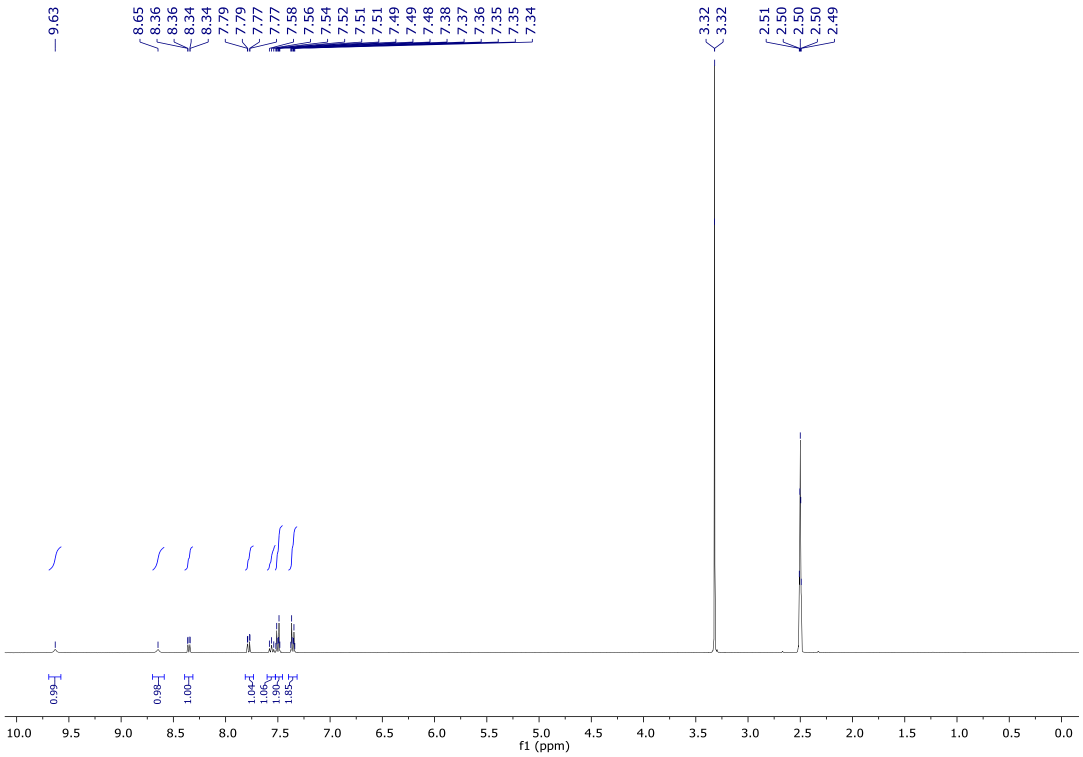

9.63
8.65 8.36 8.36 8.34 8.34 7.79 7.79 7.77 7.77 7.58 7.56 7.54 7.52 7.51 7.51 7.49 7.49 7.48 7.38 7.37 7.36 7.35 7.35 7.34
3.32 3.32
2.51 2.50 2.50 2.50 2.49
0.99
0.98
1.00
1.04
1.06
1.90
1.85
10.0 9.5 9.0 8.5 8.0 7.5 7.0 6.5 6.0 5.5 5.0 4.5 4.0 3.5 3.0 2.5 2.0 1.5 1.0 0.5 0.0
f1 (ppm)

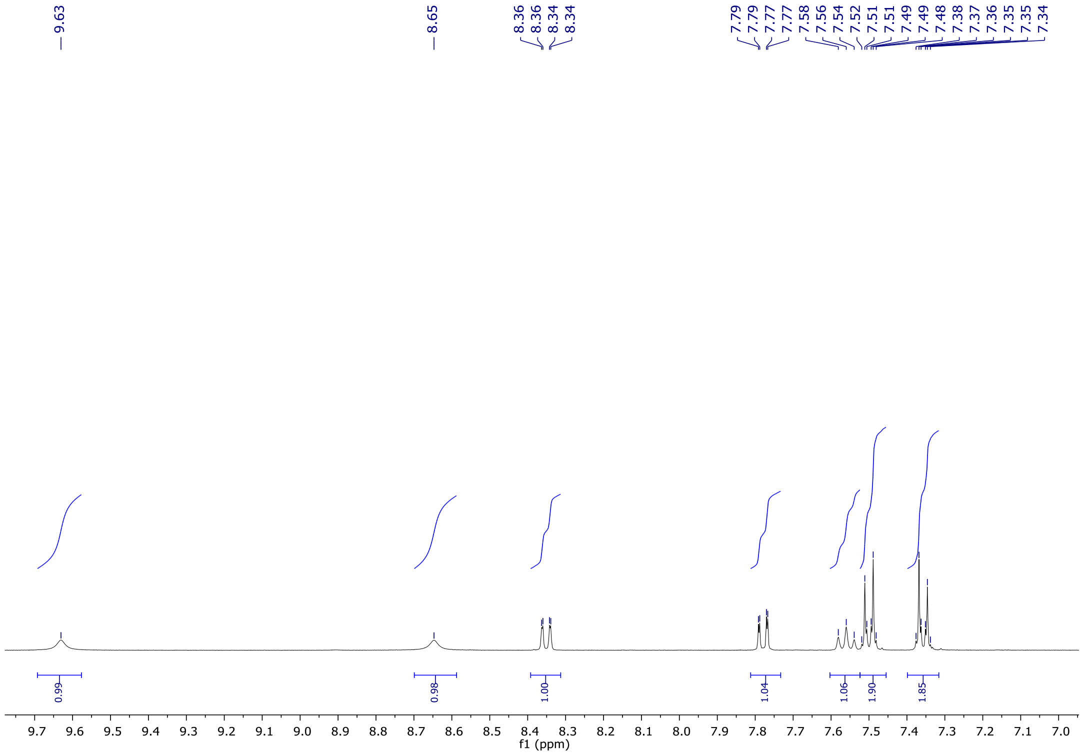

9.63
8.65
8.36 8.36 8.34 8.34
7.79 7.79 7.77 7.77 7.58 7.56 7.54 7.52 7.51 7.51 7.49 7.49 7.48 7.38 7.37 7.36 7.35 7.35 7.34
0.99
0.98
1.00
1.04
1.06
1.90
1.85
9.7 9.6 9.5 9.4 9.3 9.2 9.1 9.0 8.9 8.8 8.7 8.6 8.5 8.4 8.3 8.2 8.1 8.0 7.9 7.8 7.7 7.6 7.5 7.4 7.3 7.2 7.1 7.0
f1 (ppm)

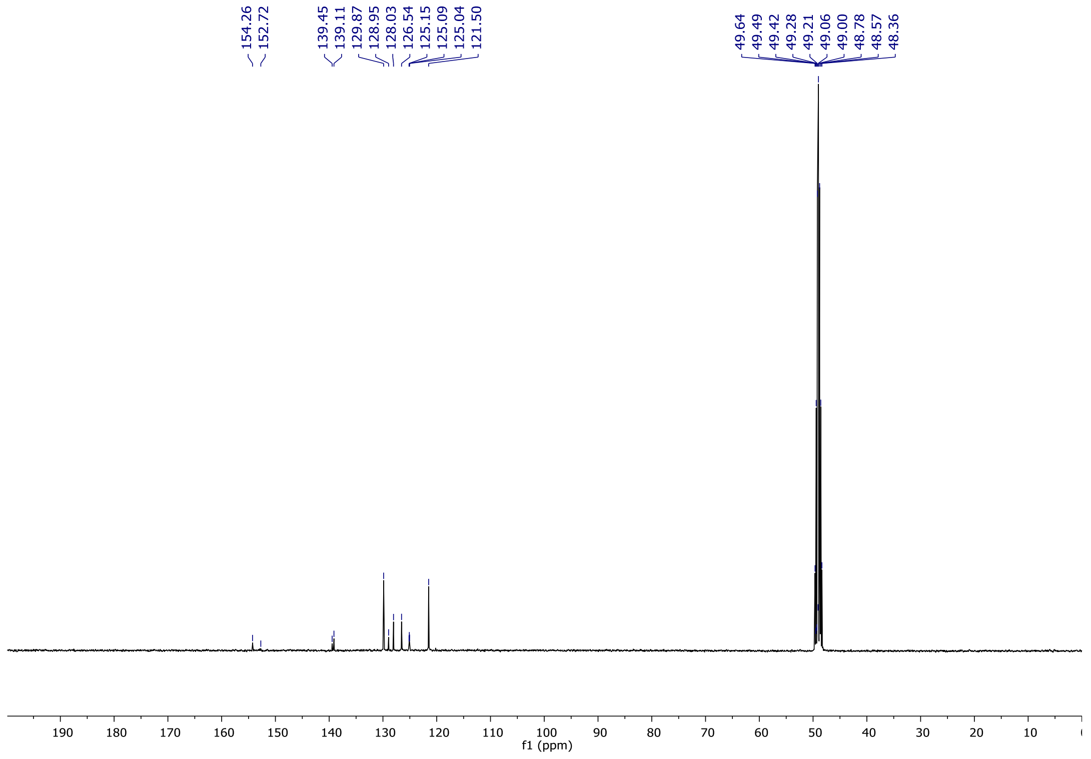

154.26
152.72
139.45
139.11
129.87
128.95
128.03
126.54
125.15
125.09
125.04
121.50
49.64
49.49
49.42
49.28
49.21
49.06
49.00
48.78
48.57
48.36
f1 (ppm)

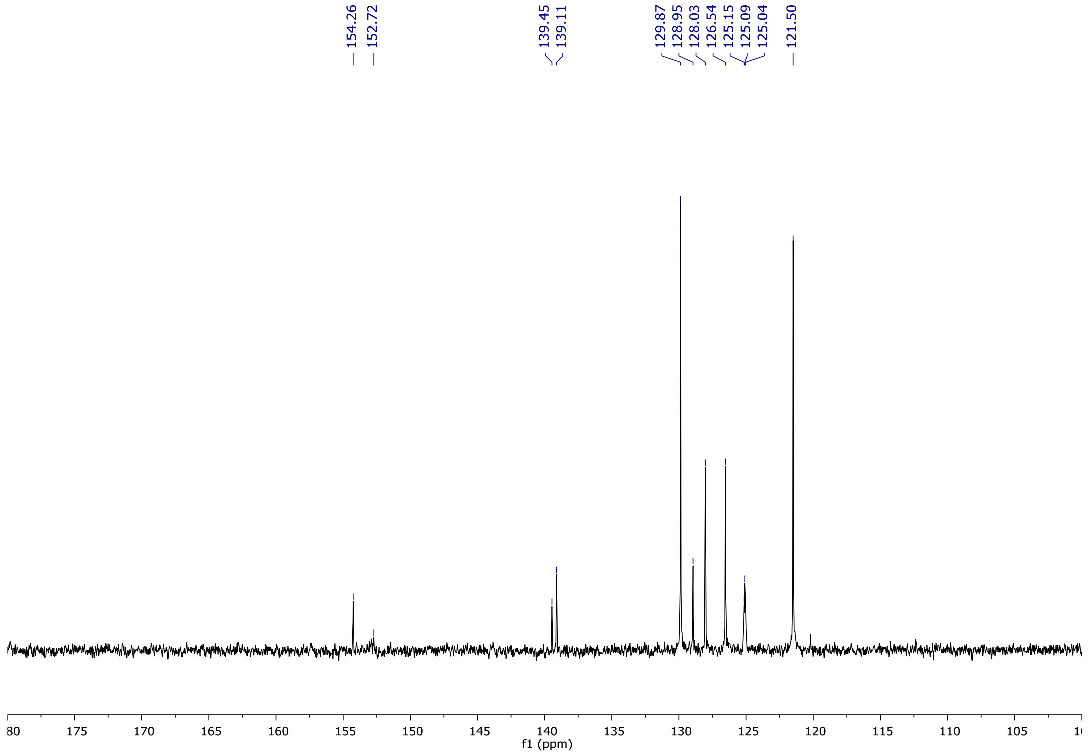

154.26
152.72
139.45
139.11
129.87
128.95
128.03
126.54
125.15
125.09
125.04
121.50
f1 (ppm)

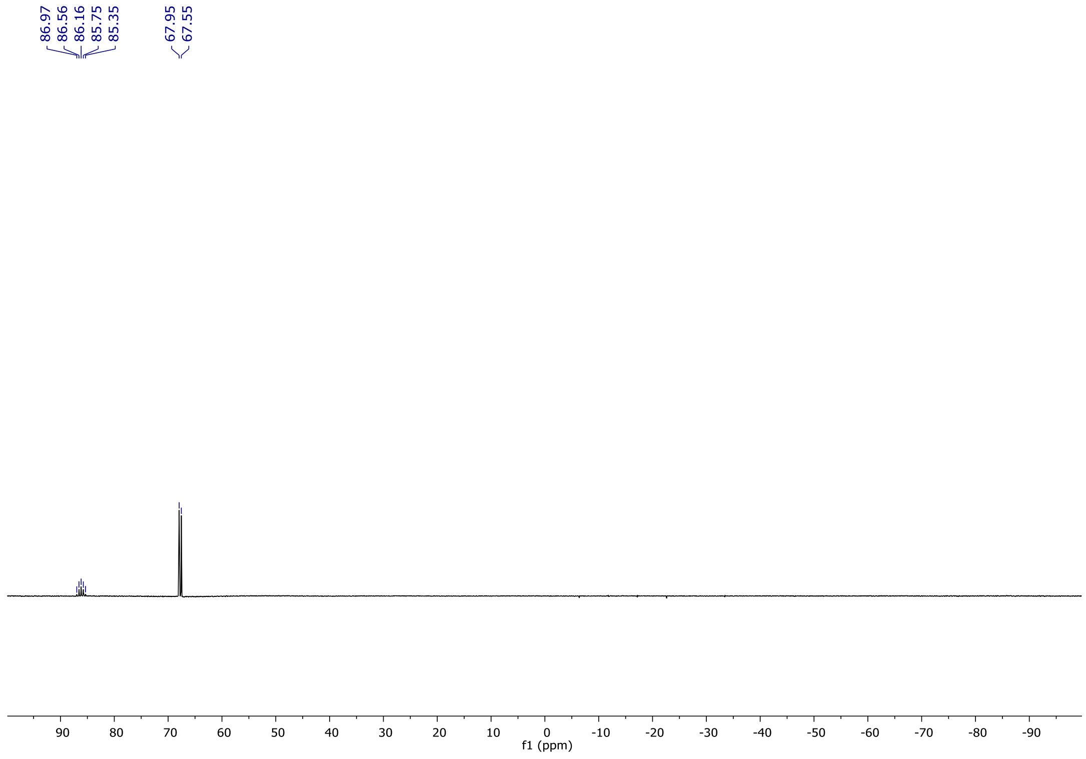
86.97
86.56
86.16
85.75
85.35
67.95
67.55
90 80 70 60 50 40 30 20 10 0 -10 -20 -30 -40 -50 -60 -70 -80 -90
f1 (ppm)

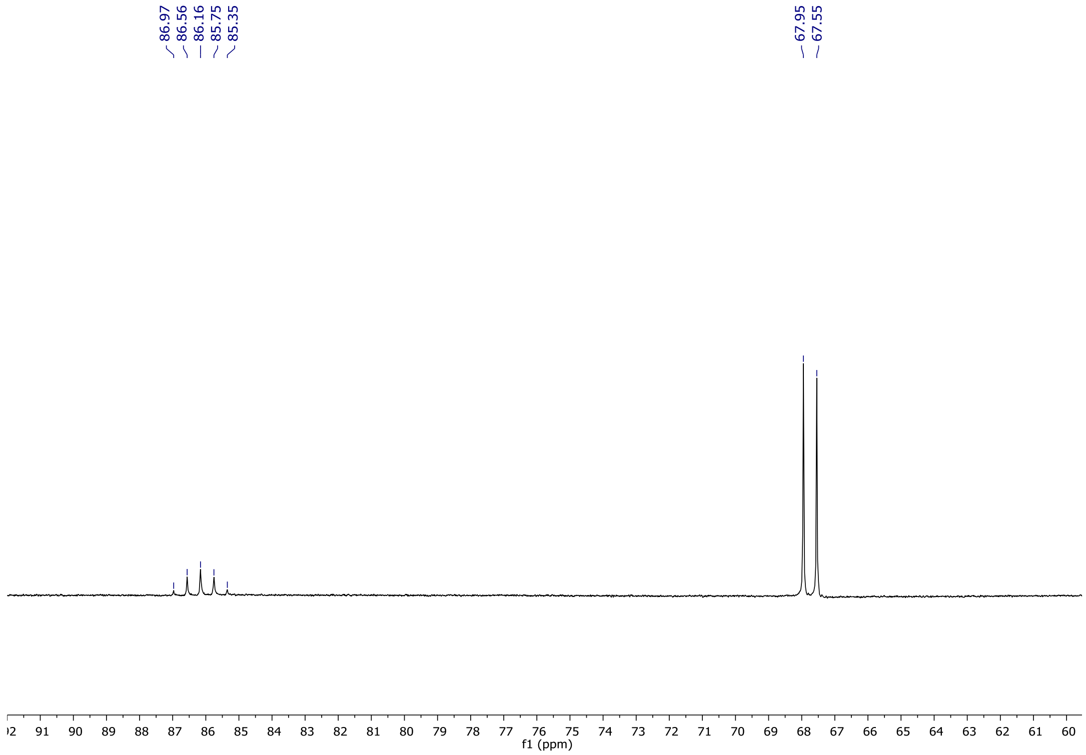
86.97
86.56
86.16
85.75
85.35
67.95
67.55
91 90 89 88 87 86 85 84 83 82 81 80 79 78 77 76 75 74 73 72 71 70 69 68 67 66 65 64 63 62 61 60
f1 (ppm)

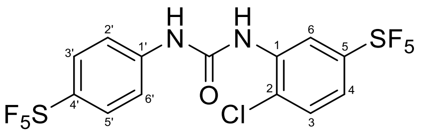

H
N
H
N
SF5
F5S
O
Cl
1'
2'
3'
4'
5'
6'
1
2
3
4
5
6

5

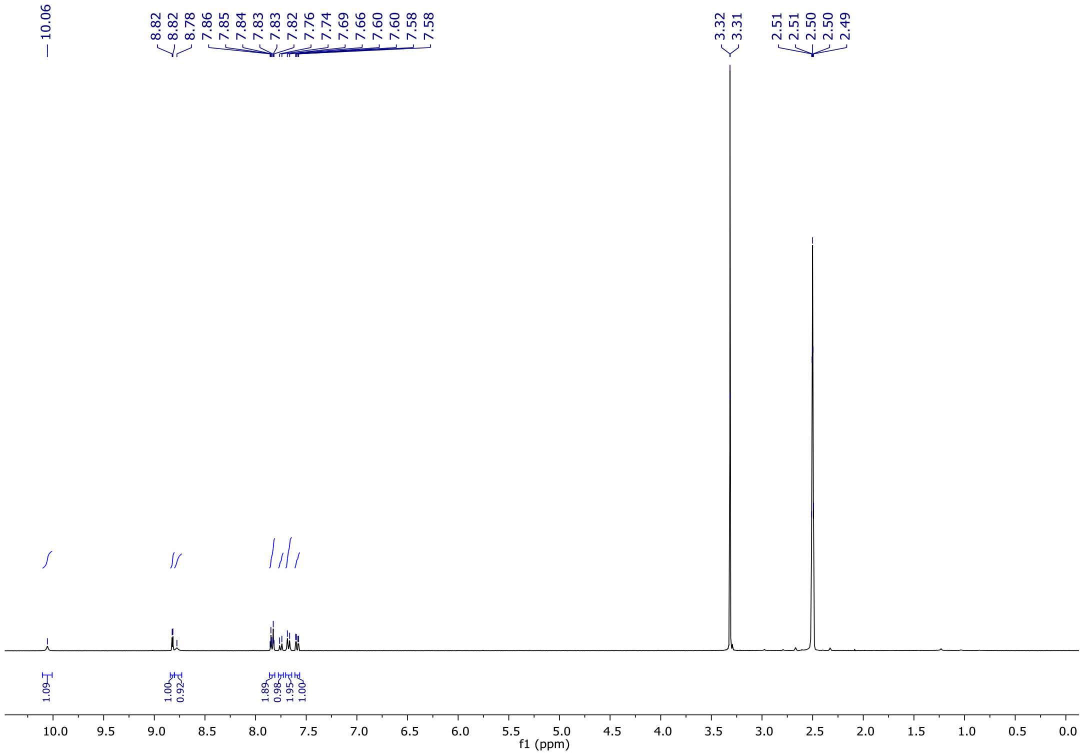

10.06
8.82
8.82
8.78
7.86
7.85
7.84
7.83
7.83
7.82
7.76
7.74
7.69
7.66
7.60
7.60
7.58
7.58
3.32
3.31
2.51
2.51
2.50
2.50
2.49
1.09
1.00
0.92
1.89
0.98
1.95
1.00
10.0
9.5
9.0
8.5
8.0
7.5
7.0
6.5
6.0
5.5
5.0
4.5
4.0
3.5
3.0
2.5
2.0
1.5
1.0
0.5
0.0
f1 (ppm)

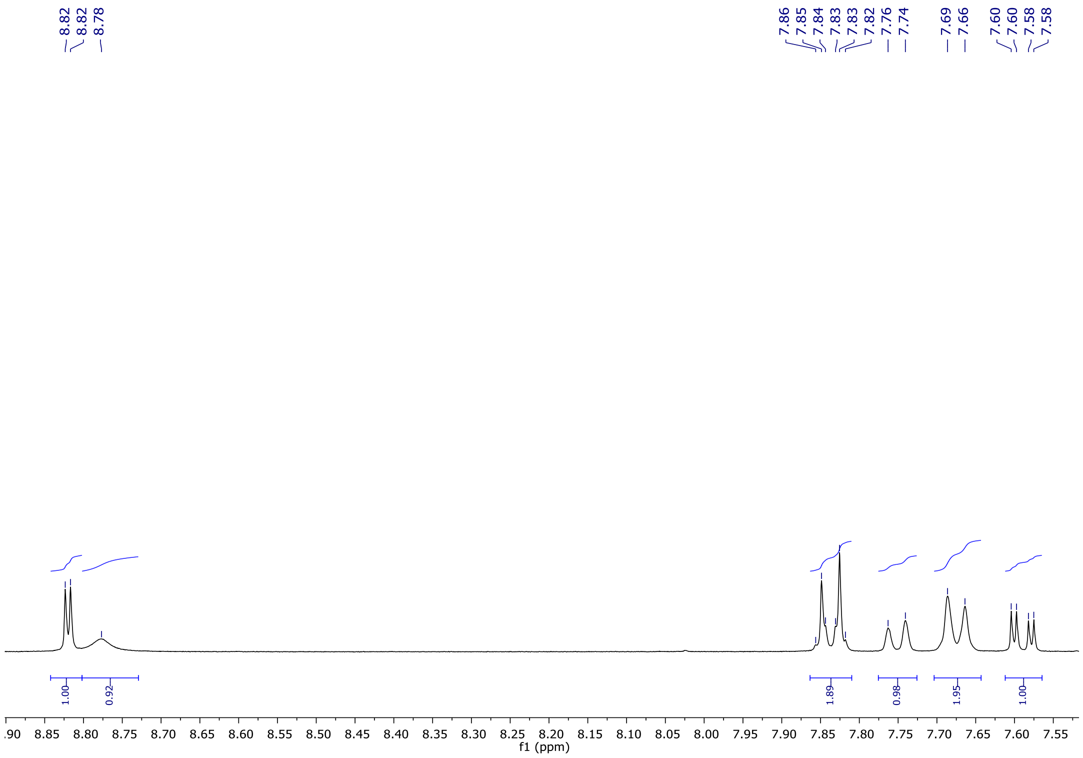

8.82
8.82
8.78
7.86
7.85
7.84
7.83
7.83
7.82
7.76
7.74
7.69
7.66
7.60
7.60
7.58
7.58
1.00
0.92
1.89
0.98
1.95
1.00
.90
8.85
8.80
8.75
8.70
8.65
8.60
8.55
8.50
8.45
8.40
8.35
8.30
8.25
8.20
8.15
8.10
8.05
8.00
7.95
7.90
7.85
7.80
7.75
7.70
7.65
7.60
7.55
f1 (ppm)

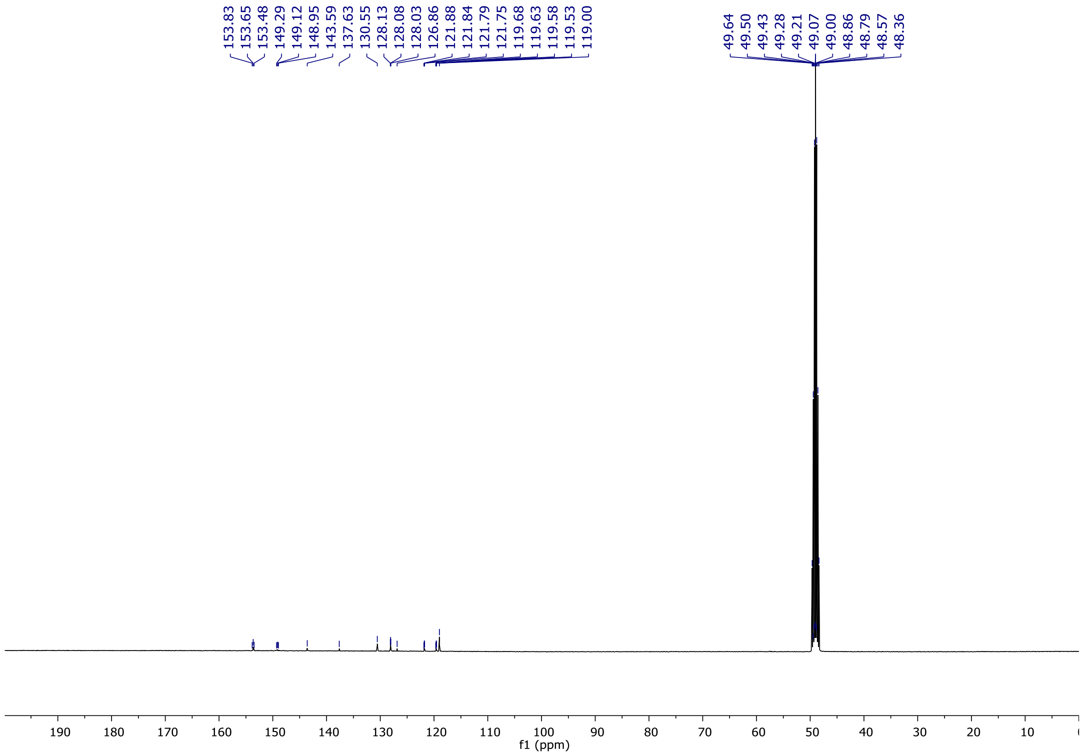
153.83
153.65
153.48
149.29
149.12
148.95
143.59
137.63
130.55
128.13
128.08
128.03
126.86
121.88
121.84
121.79
121.75
119.68
119.63
119.58
119.53
119.00
49.64
49.50
49.43
49.28
49.21
49.07
49.00
48.86
48.79
48.57
48.36
190 180 170 160 150 140 130 120 110 100 90 80 70 60 50 40 30 20 10
f1 (ppm)

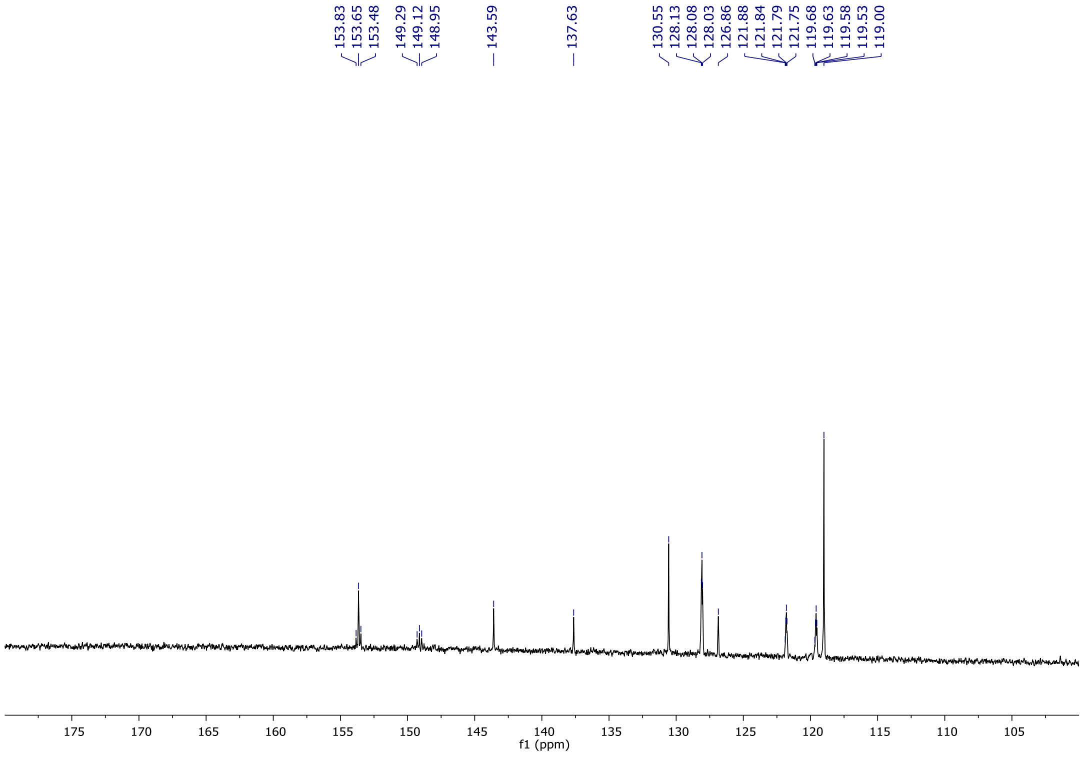
153.83
153.65
153.48
149.29
149.12
148.95
143.59
137.63
130.55
128.13
128.08
128.03
126.86
121.88
121.84
121.79
121.75
119.68
119.63
119.58
119.53
119.00
175 170 165 160 155 150 145 140 135 130 125 120 115 110 105
f1 (ppm)

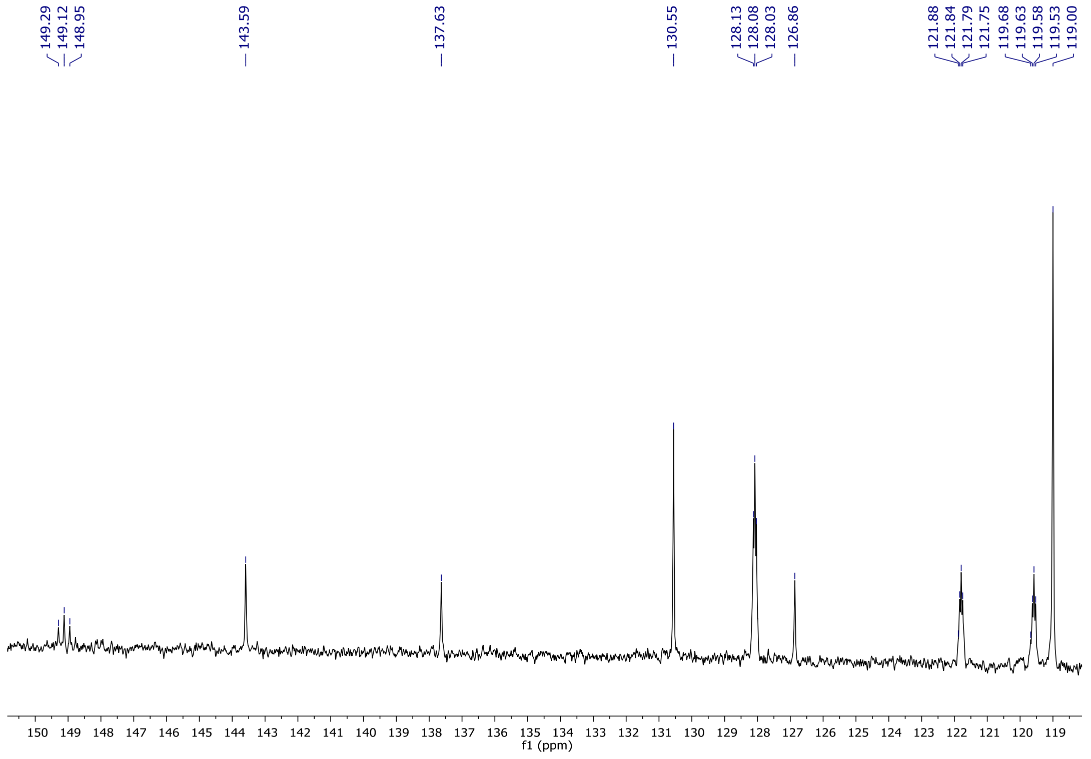
149.29
149.12
148.95
143.59
137.63
130.55
128.13
128.08
128.03
126.86
121.88
121.84
121.79
121.75
119.68
119.63
119.58
119.53
119.00
f1 (ppm)

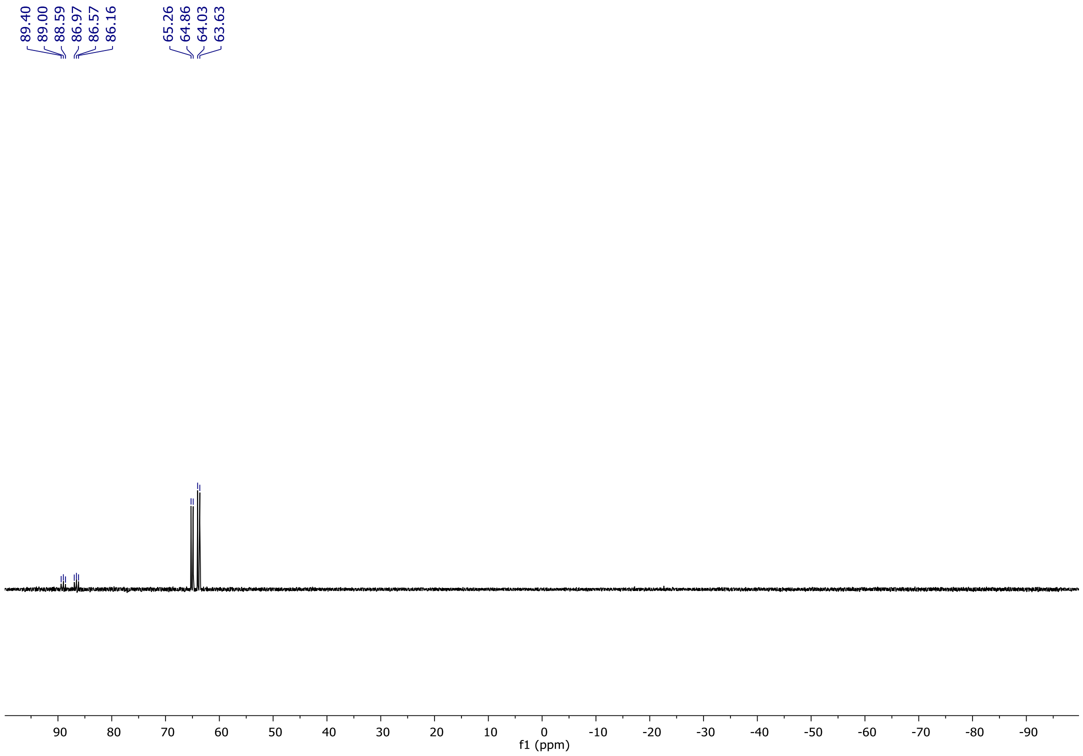
89.40
89.00
88.59
86.97
86.57
86.16
65.26
64.86
64.03
63.63
f1 (ppm)

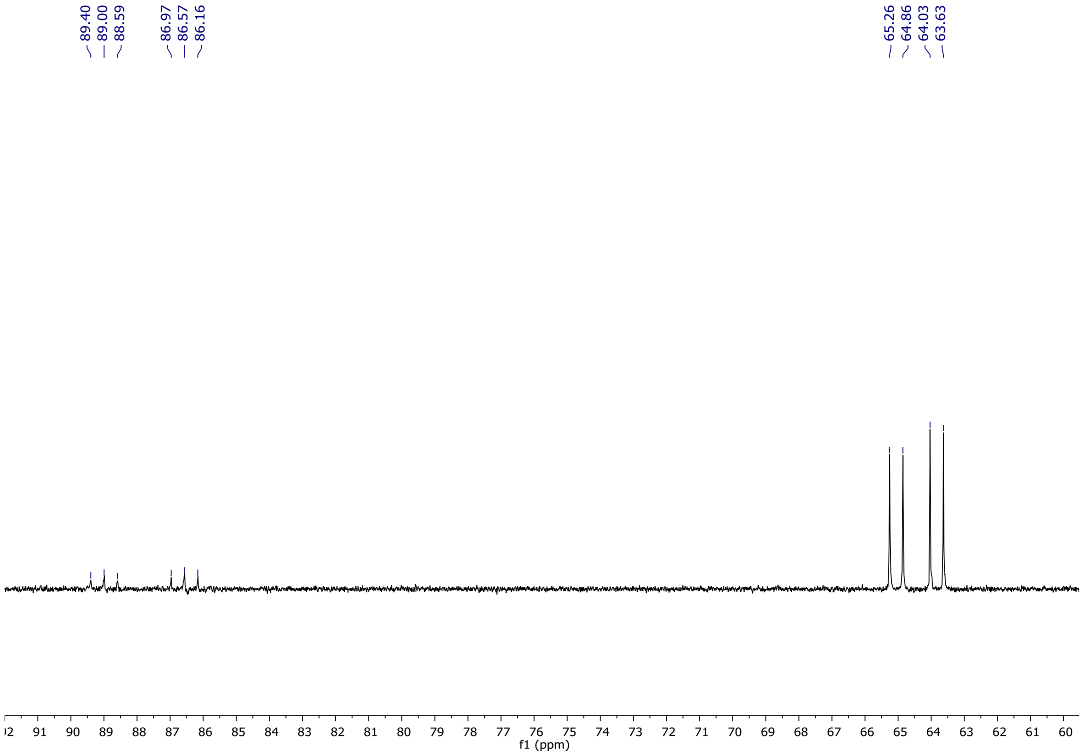
89.40
89.00
88.59
86.97
86.57
86.16
65.26
64.86
64.03
63.63
f1 (ppm)

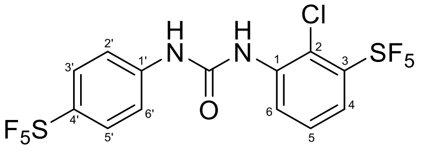
Cl
SF5
F5S
H
N
O
1
2
3
4
5
6
1'
2'
3'
4'
5'
6'

6

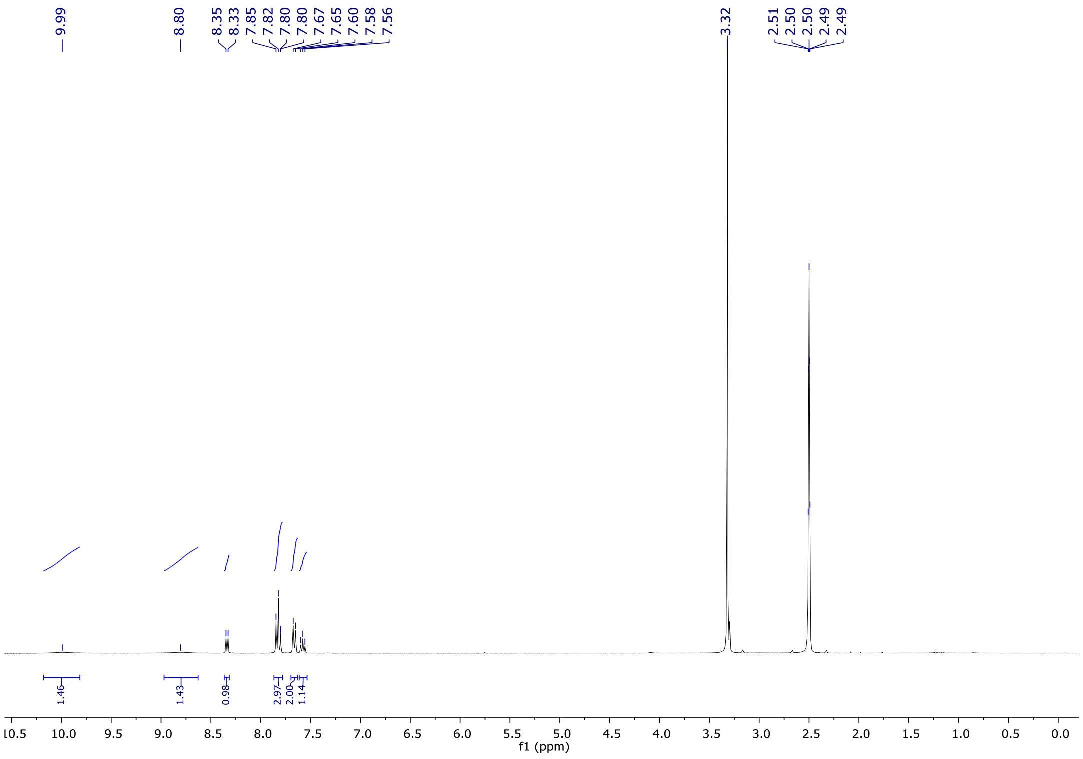
9.99
8.80
8.35
8.33
7.85
7.82
7.80
7.80
7.67
7.65
7.60
7.58
7.56
3.32
2.51
2.50
2.50
2.49
2.49
1.46
1.43
0.98
2.97
2.00
1.14
f1 (ppm)

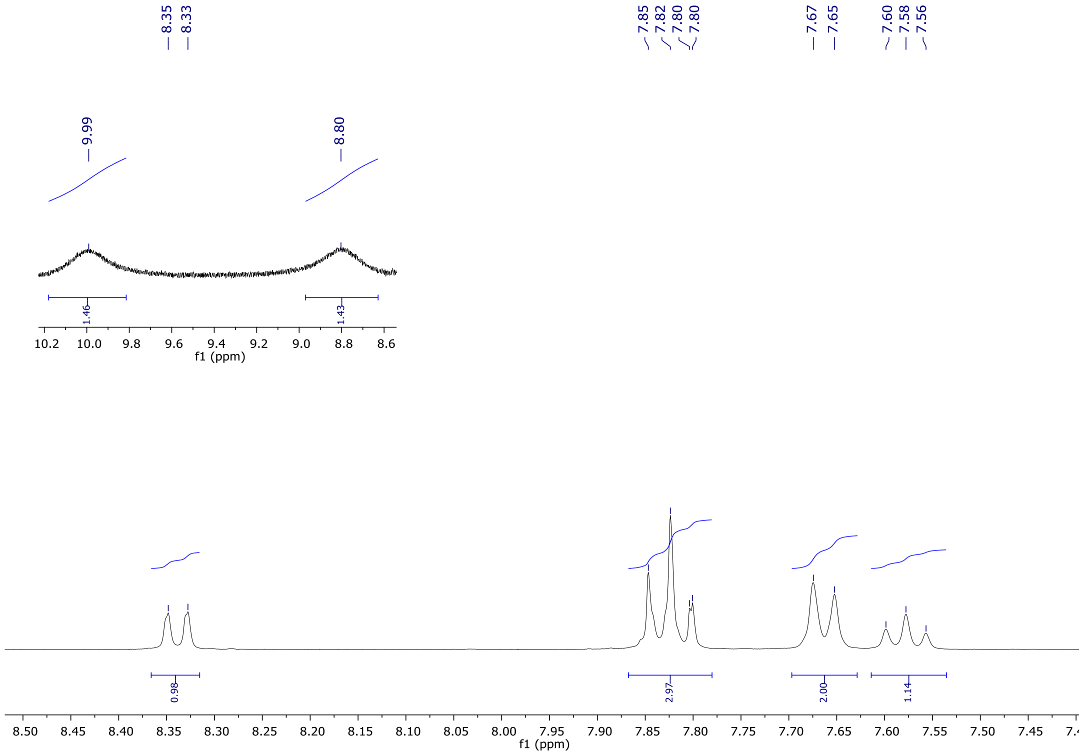
8.35
8.33
7.85
7.82
7.80
7.80
7.67
7.65
7.60
7.58
7.56
9.99
8.80
1.46
1.43
f1 (ppm)
0.98
2.97
2.00
1.14
f1 (ppm)

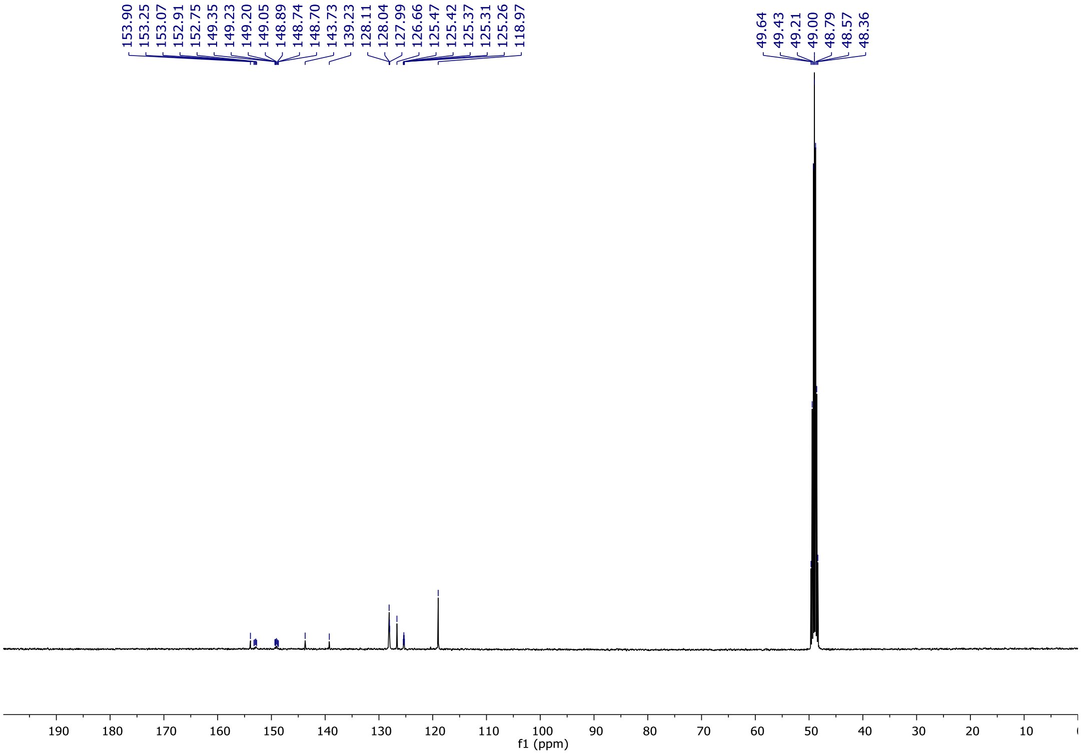
153.90
153.25
153.07
152.91
152.75
149.35
149.23
149.20
149.05
148.89
148.74
148.70
143.73
139.23
128.11
128.04
127.99
126.66
125.47
125.42
125.37
125.31
125.26
118.97
49.64
49.43
49.21
49.00
48.79
48.57
48.36
f1 (ppm)

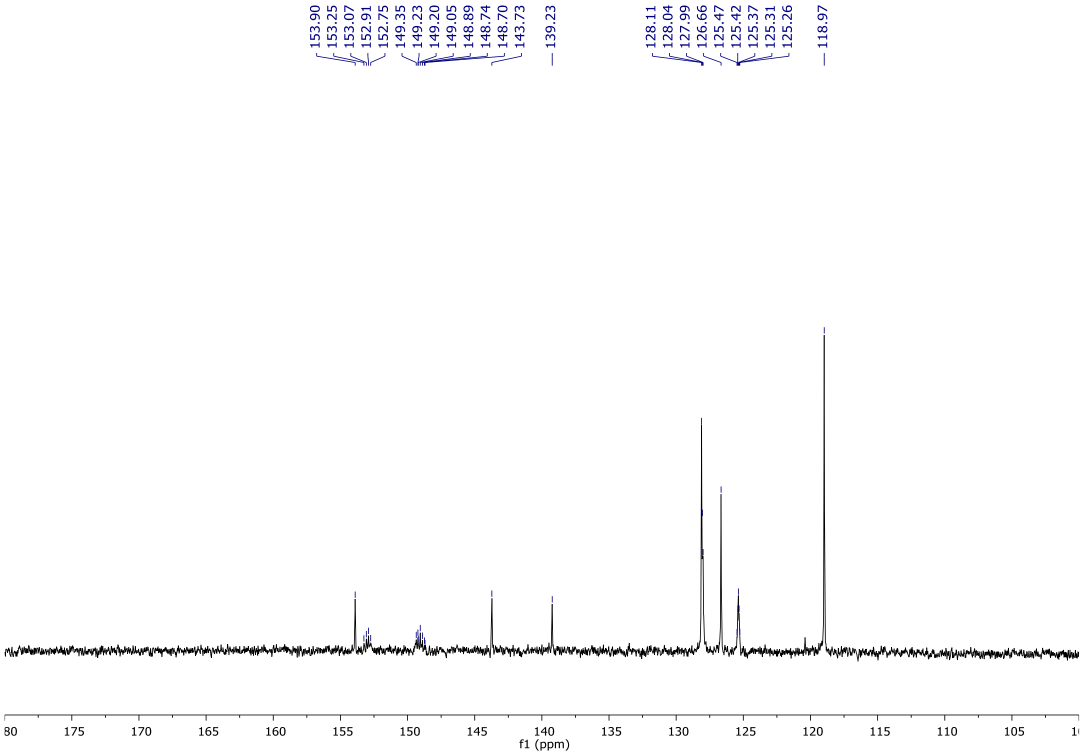
153.90
153.25
153.07
152.91
152.75
149.35
149.23
149.20
149.05
148.89
148.74
148.70
143.73
139.23
128.11
128.04
127.99
126.66
125.47
125.42
125.37
125.31
125.26
118.97
f1 (ppm)

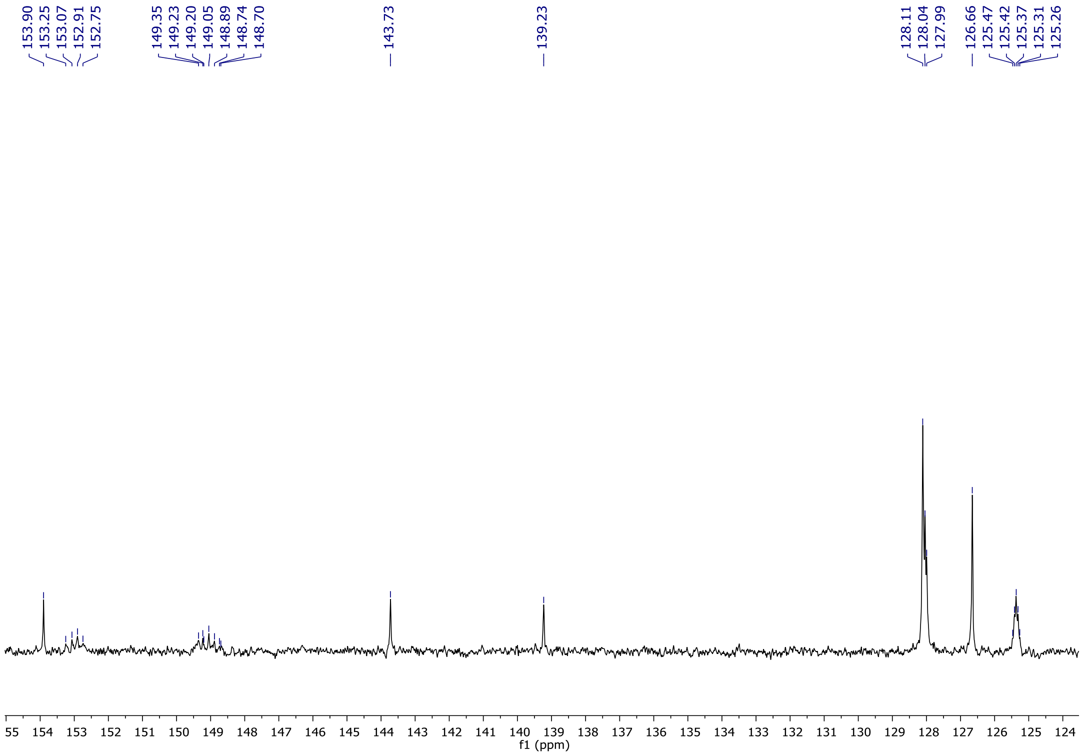
153.90
153.25
153.07
152.91
152.75
149.35
149.23
149.20
149.05
148.89
148.74
148.70
143.73
139.23
128.11
128.04
127.99
126.66
125.47
125.42
125.37
125.31
125.26
55 154 153 152 151 150 149 148 147 146 145 144 143 142 141 140 139 138 137 136 135 134 133 132 131 130 129 128 127 126 125 124
f1 (ppm)

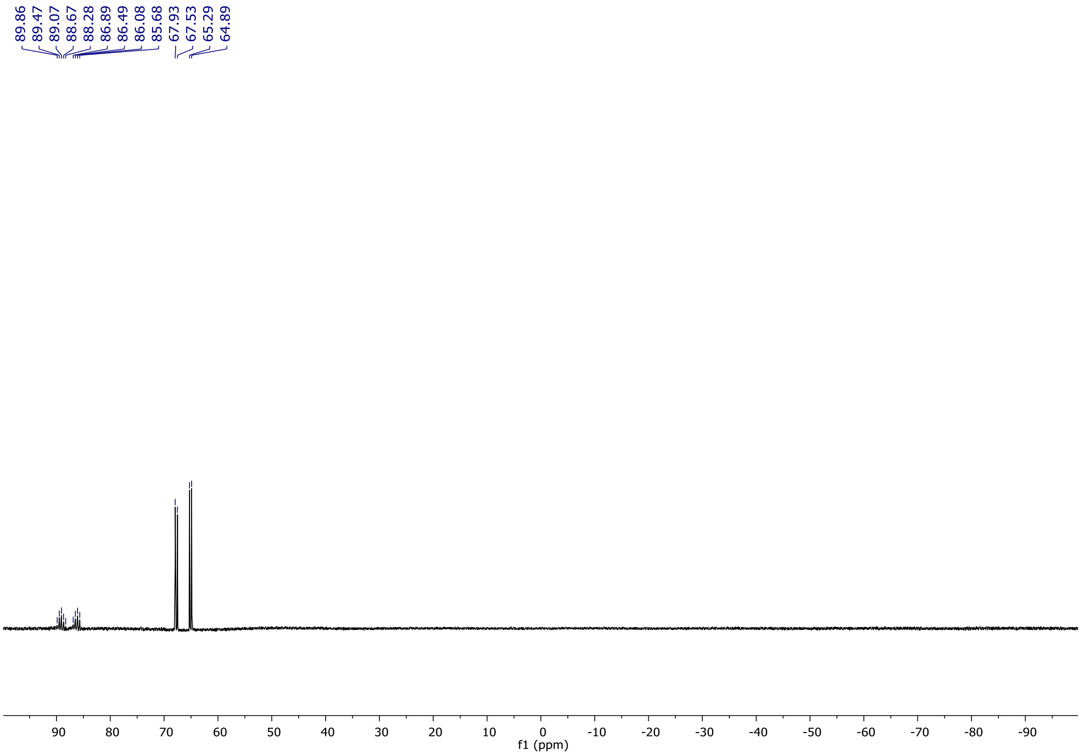
89.86
89.47
89.07
88.67
88.28
86.89
86.49
86.08
85.68
67.93
67.53
65.29
64.89
90 80 70 60 50 40 30 20 10 0 -10 -20 -30 -40 -50 -60 -70 -80 -90
f1 (ppm)

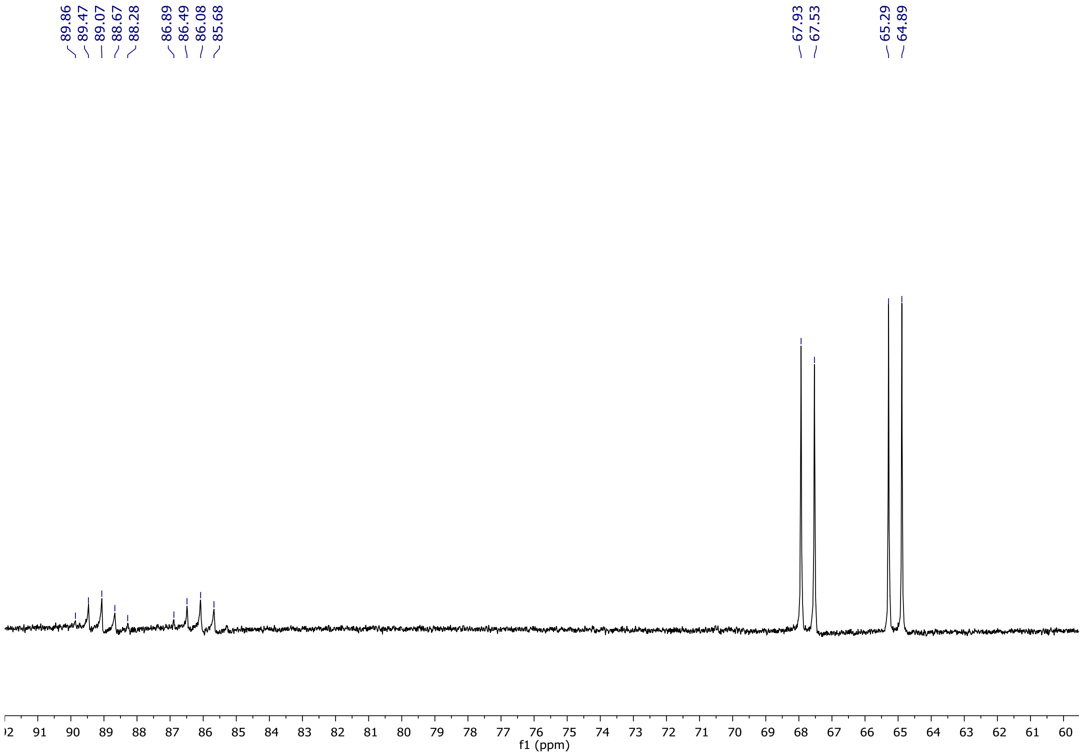
89.86
89.47
89.07
88.67
88.28
86.89
86.49
86.08
85.68
67.93
67.53
65.29
64.89
91 90 89 88 87 86 85 84 83 82 81 80 79 78 77 76 75 74 73 72 71 70 69 68 67 66 65 64 63 62 61 60
f1 (ppm)

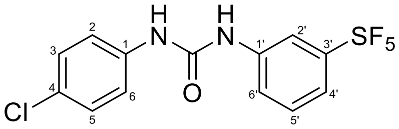
H
N
H
N
2
3
1
2'
1'
3'
SF5
Cl
4
5
6
O
6'
5'
4'

7

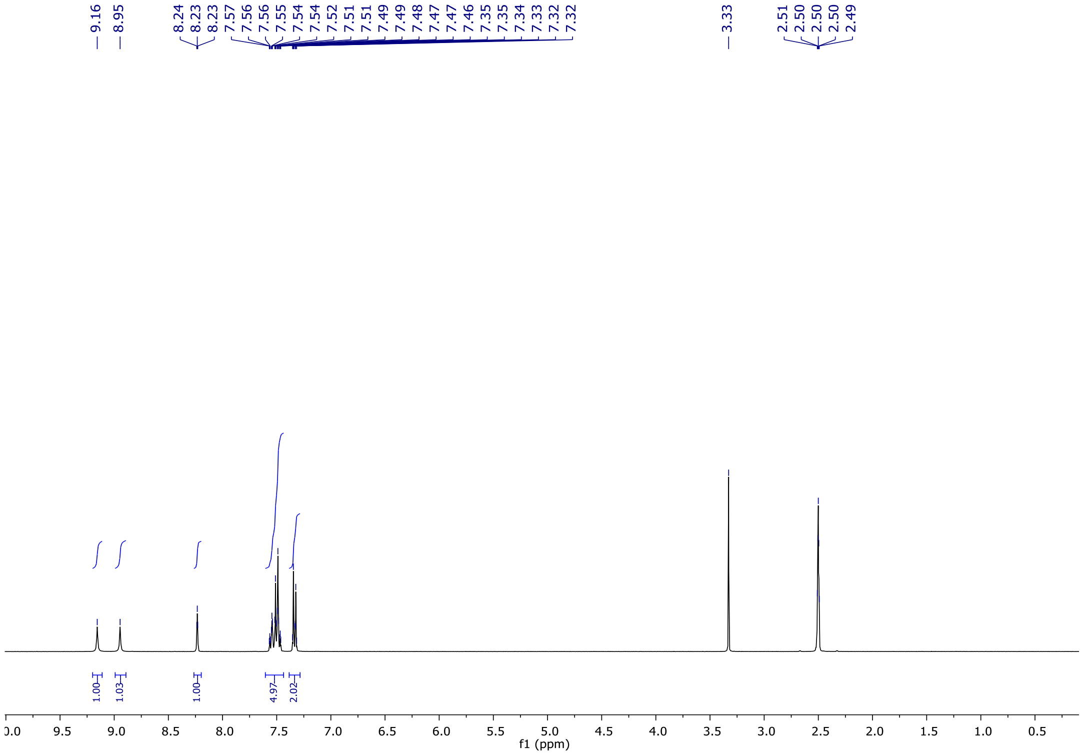
9.16
8.95
8.24
8.23
8.23
7.57
7.56
7.55
7.54
7.54
7.52
7.51
7.51
7.49
7.48
7.47
7.47
7.46
7.35
7.35
7.34
7.33
7.32
7.32
3.33
2.51
2.50
2.50
2.50
2.49
1.00
1.03
1.00
4.97
2.02
f1 (ppm)

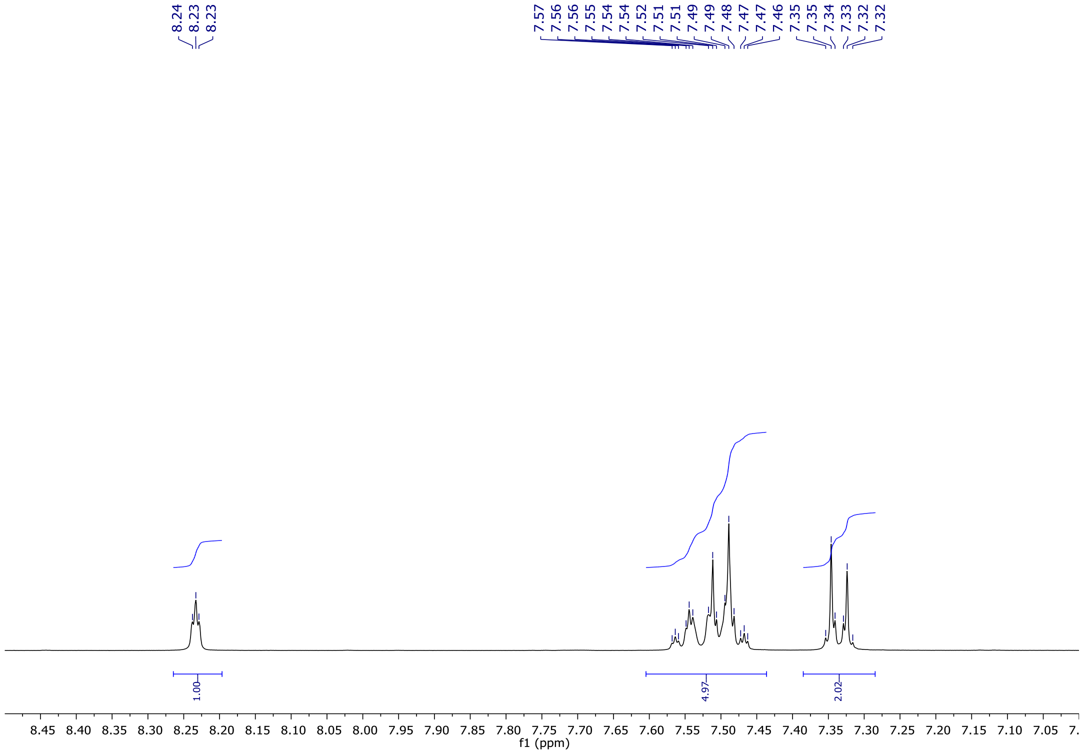
8.24
8.23
8.23
7.57
7.56
7.56
7.55
7.54
7.54
7.52
7.51
7.51
7.49
7.49
7.48
7.47
7.47
7.46
7.35
7.35
7.34
7.33
7.32
7.32
1.00
4.97
2.02
f1 (ppm)

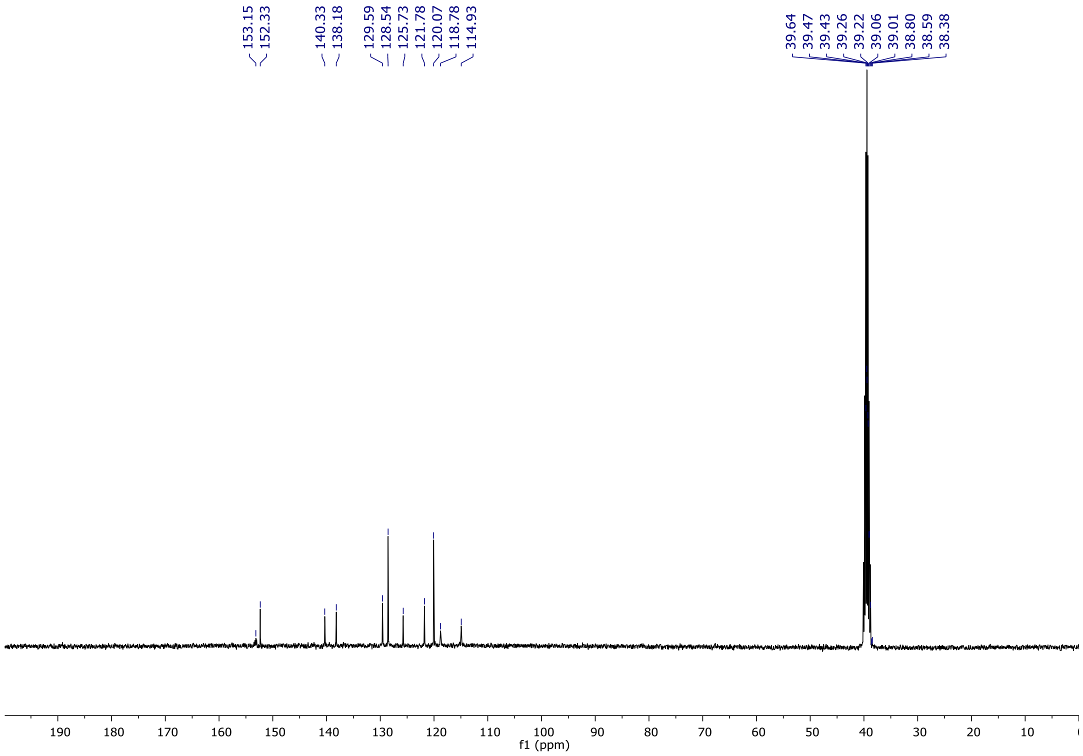
153.15
152.33
140.33
138.18
129.59
128.54
125.73
121.78
120.07
118.78
114.93
39.64
39.47
39.43
39.26
39.22
39.06
39.01
38.80
38.59
38.38
f1 (ppm)

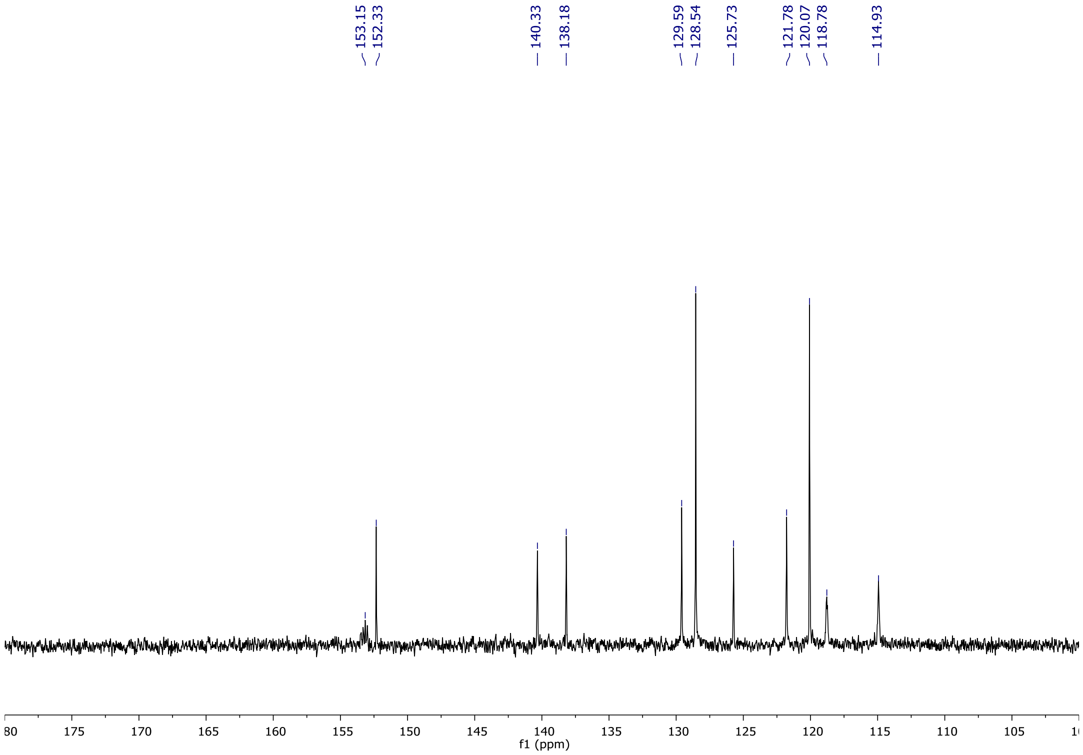
153.15
152.33
140.33
138.18
129.59
128.54
125.73
121.78
120.07
118.78
114.93
f1 (ppm)

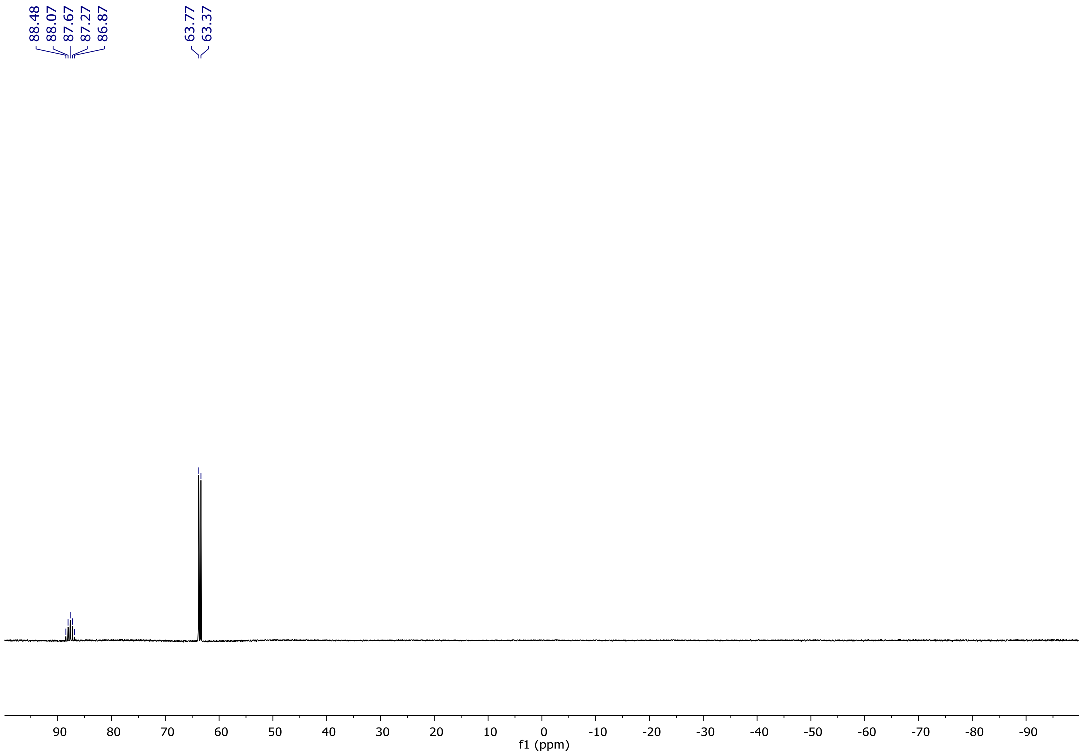
88.48
88.07
87.67
87.27
86.87
63.77
63.37
90 80 70 60 50 40 30 20 10 0 -10 -20 -30 -40 -50 -60 -70 -80 -90
f1 (ppm)

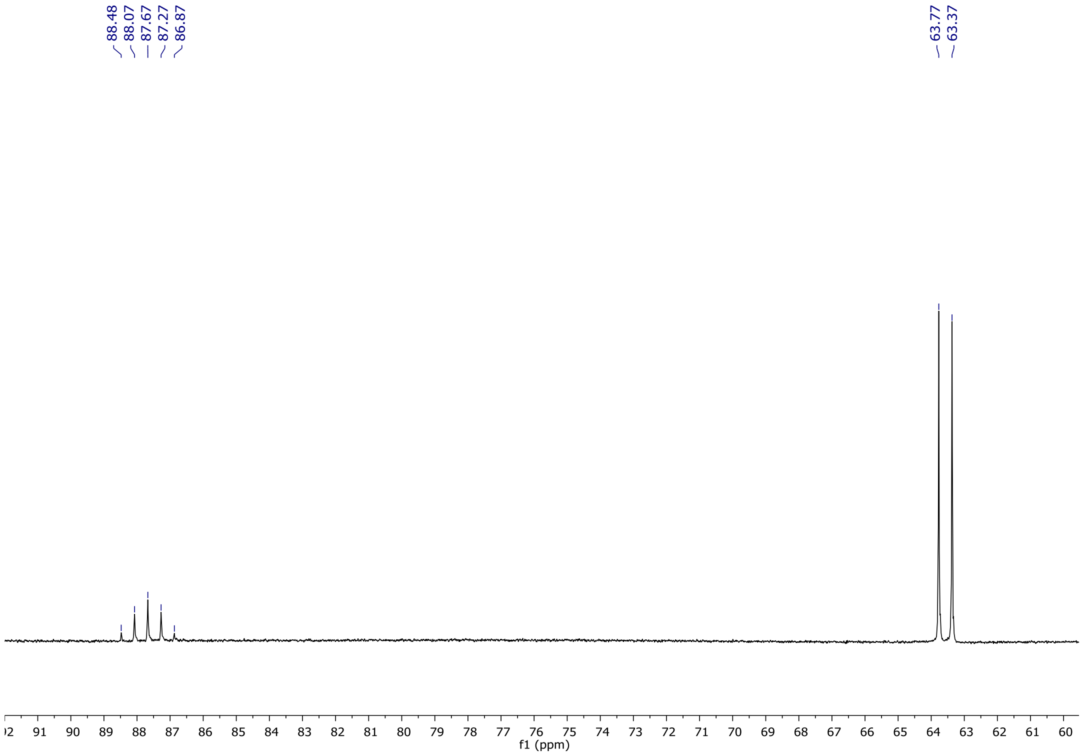
88.48
88.07
87.67
87.27
86.87
63.77
63.37
91 90 89 88 87 86 85 84 83 82 81 80 79 78 77 76 75 74 73 72 71 70 69 68 67 66 65 64 63 62 61 60
f1 (ppm)

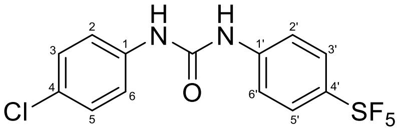

8

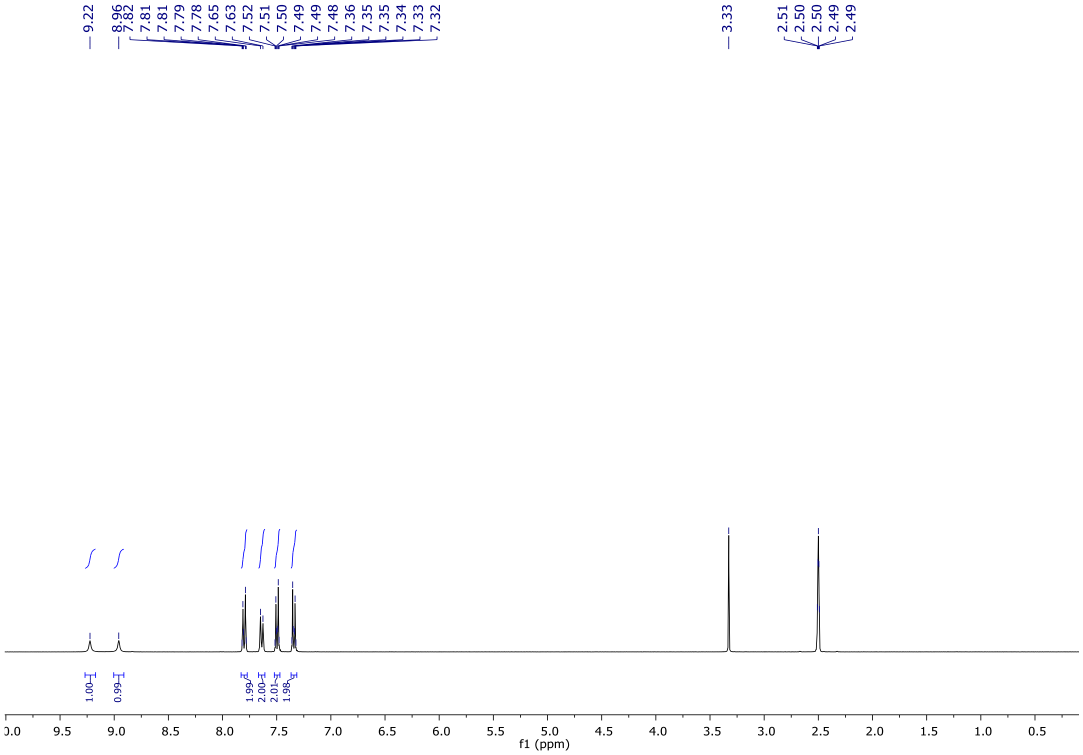

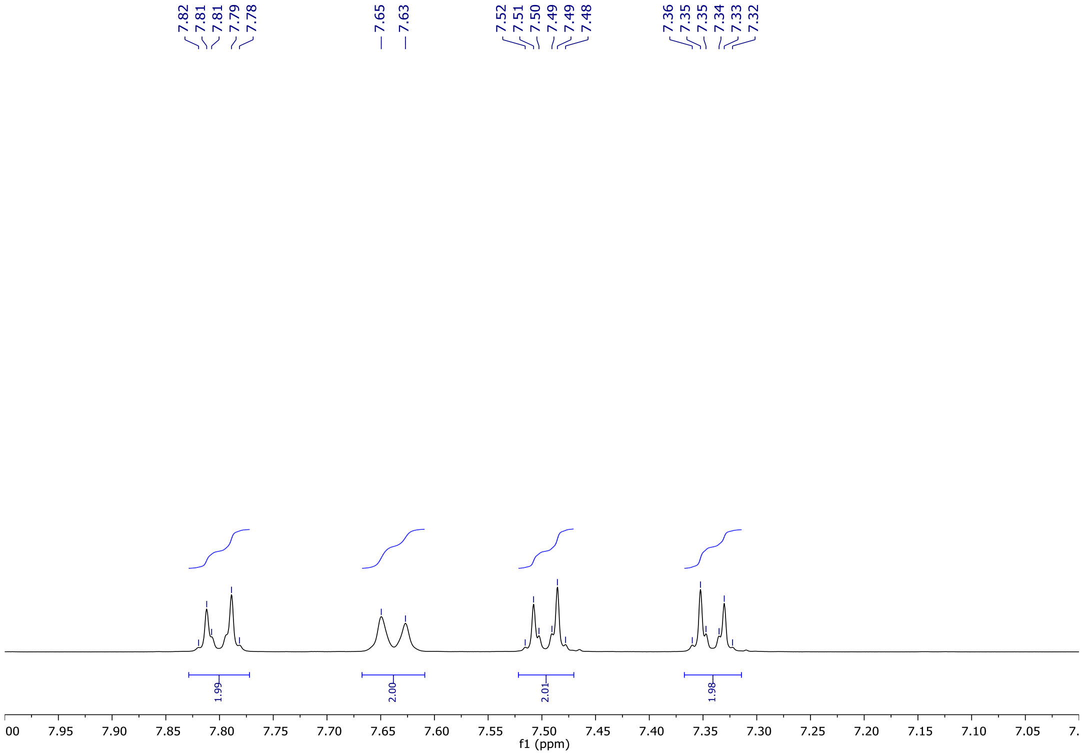

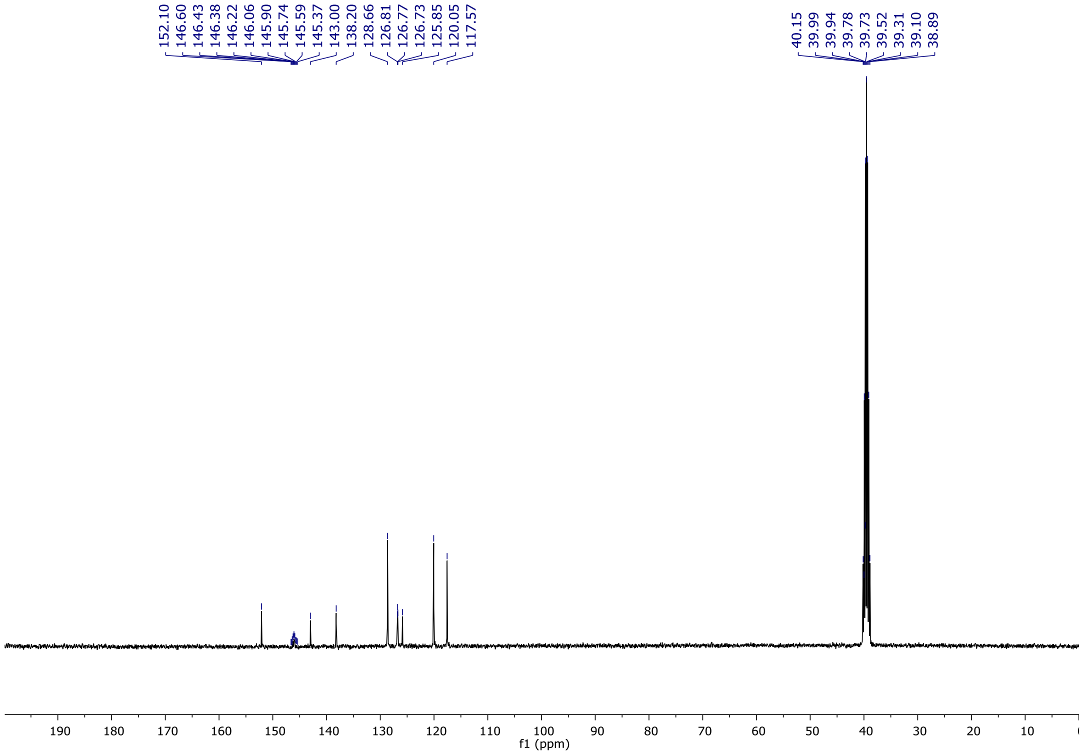
152.10
146.60
146.43
146.38
146.22
146.06
145.90
145.74
145.59
145.37
143.00
138.20
128.66
126.81
126.77
126.73
125.85
120.05
117.57
40.15
39.99
39.94
39.78
39.73
39.52
39.31
39.10
38.89
f1 (ppm)

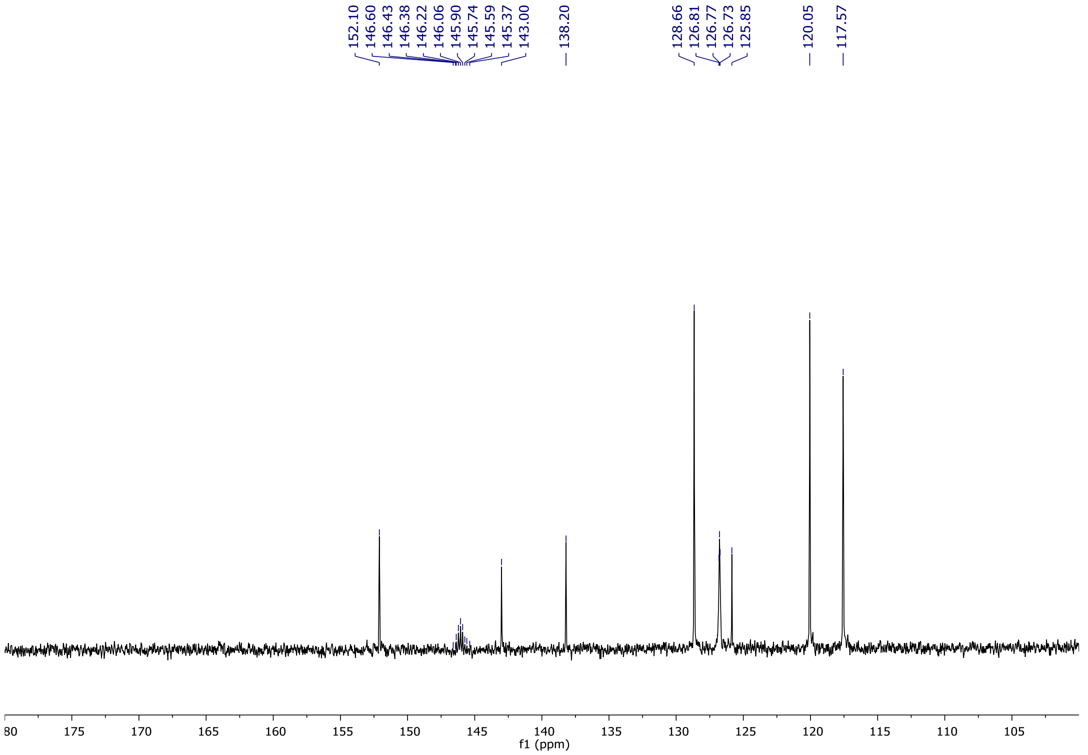
152.10
146.60
146.43
146.38
146.22
146.06
145.90
145.74
145.59
145.37
143.00
138.20
128.66
126.81
126.77
126.73
125.85
120.05
117.57
f1 (ppm)

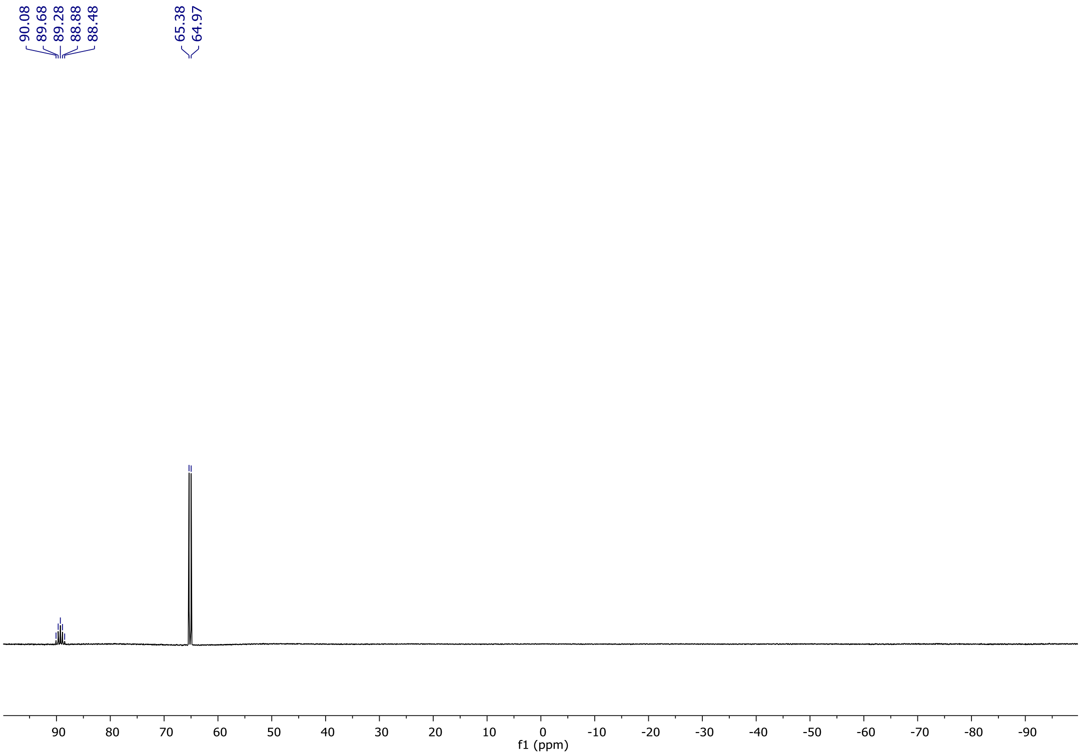
90.08
89.68
89.28
88.88
88.48
65.38
64.97
f1 (ppm)

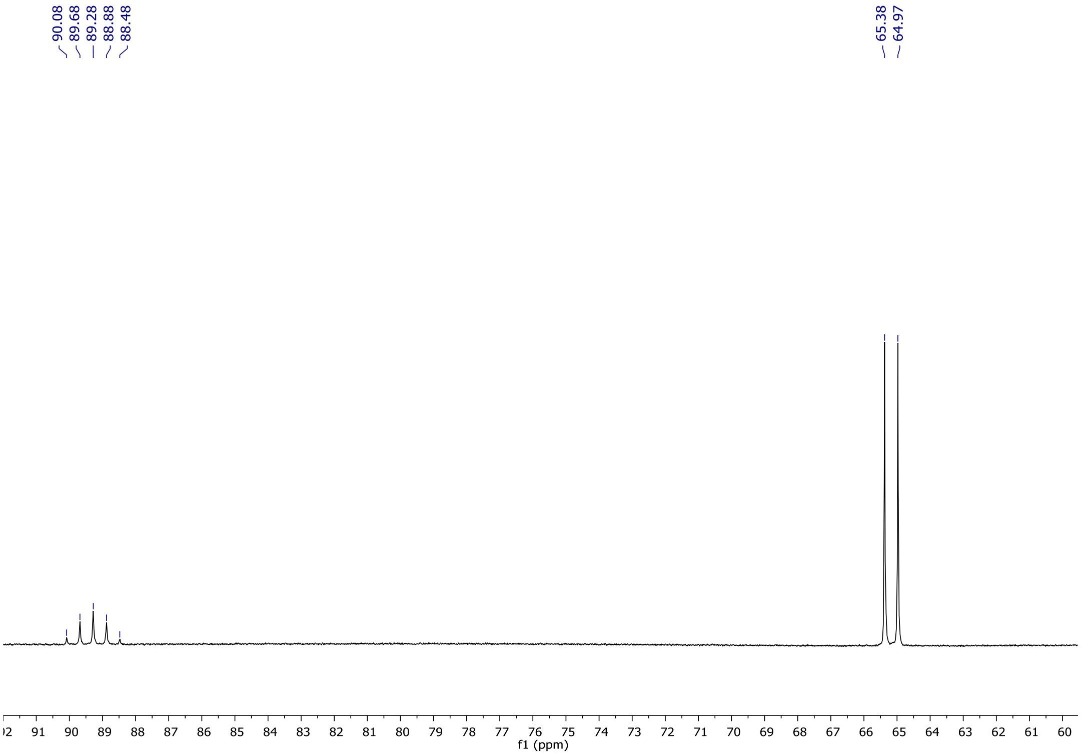
90.08
89.68
89.28
88.88
88.48
65.38
64.97
f1 (ppm)

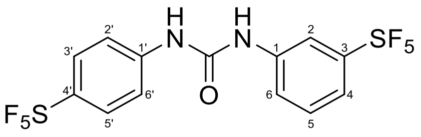
SF5
F5S
H
N
O
1
2
3
4
5
6
1'
2'
3'
4'
5'
6'

9

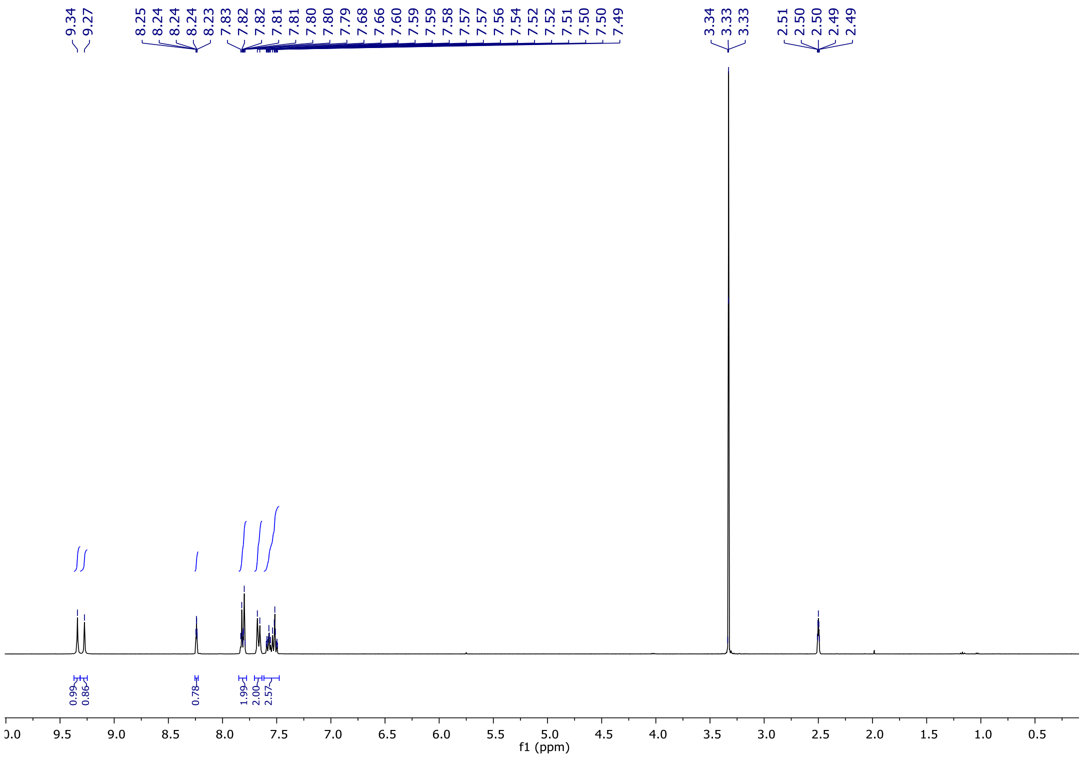
9.34
9.27
8.25
8.24
8.24
8.24
8.23
7.83
7.82
7.82
7.81
7.81
7.80
7.80
7.79
7.68
7.66
7.60
7.59
7.59
7.58
7.57
7.57
7.56
7.54
7.52
7.52
7.51
7.50
7.50
7.49
3.34
3.33
3.33
2.51
2.50
2.50
2.49
2.49
0.99
0.86
0.78
1.99
2.00
2.57
f1 (ppm)

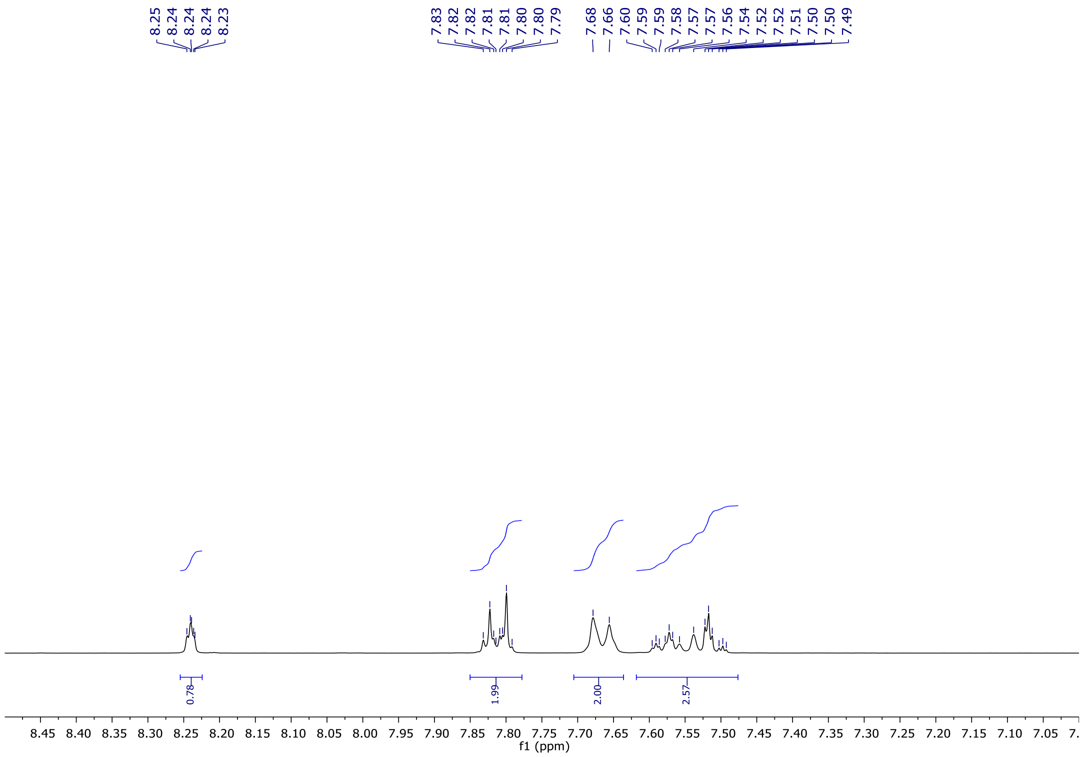
8.25
8.24
8.24
8.24
8.23
7.83
7.82
7.82
7.81
7.81
7.80
7.80
7.79
7.68
7.66
7.60
7.59
7.59
7.58
7.57
7.57
7.56
7.54
7.52
7.52
7.51
7.50
7.50
7.49
0.78
1.99
2.00
2.57
f1 (ppm)

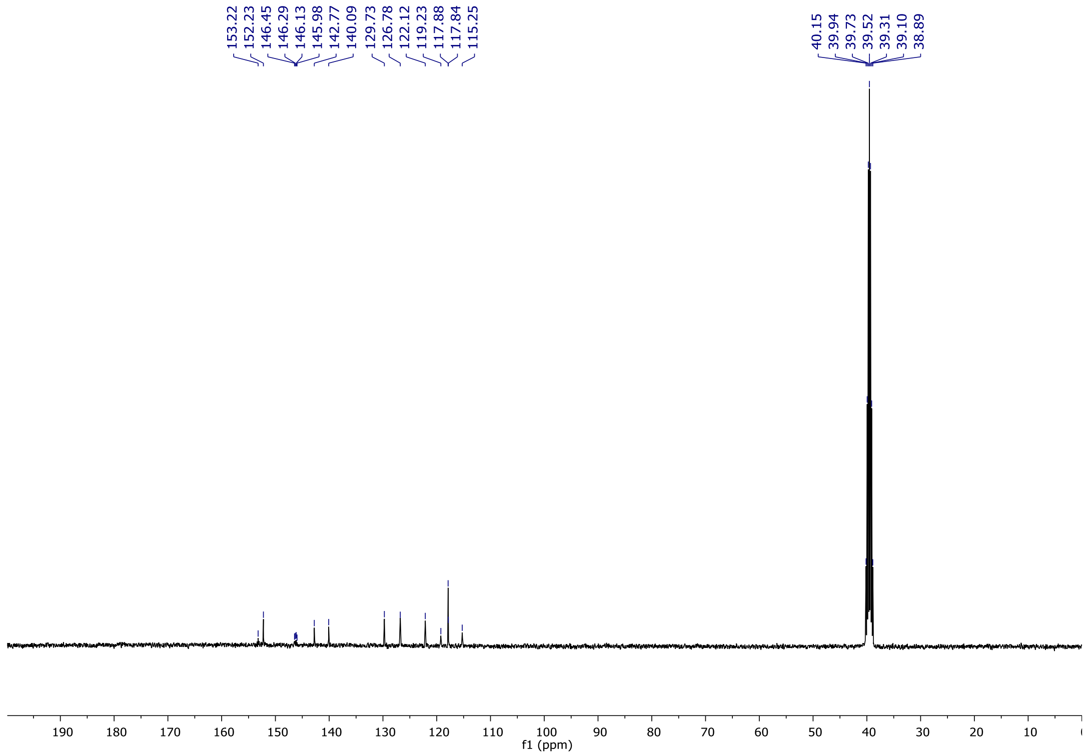
153.22
152.23
146.45
146.29
146.13
145.98
142.77
140.09
129.73
126.78
122.12
119.23
117.88
117.84
115.25
40.15
39.94
39.73
39.52
39.31
39.10
38.89
f1 (ppm)

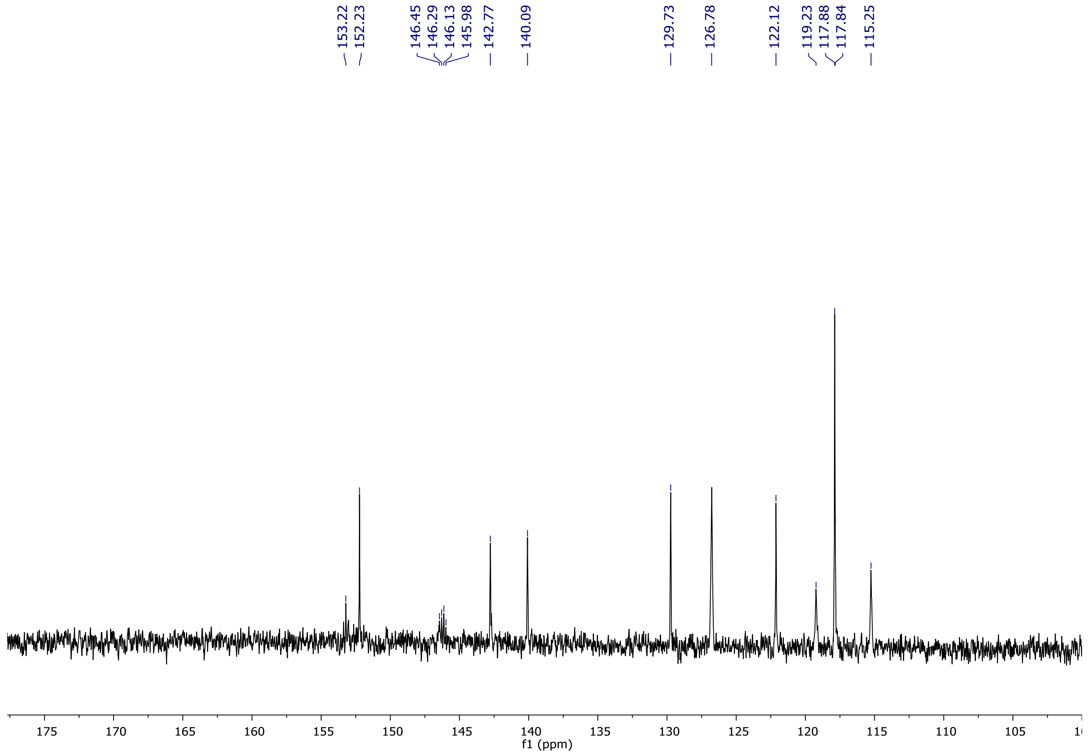
153.22
152.23
146.45
146.29
146.13
145.98
142.77
140.09
129.73
126.78
122.12
119.23
117.88
117.84
115.25
f1 (ppm)

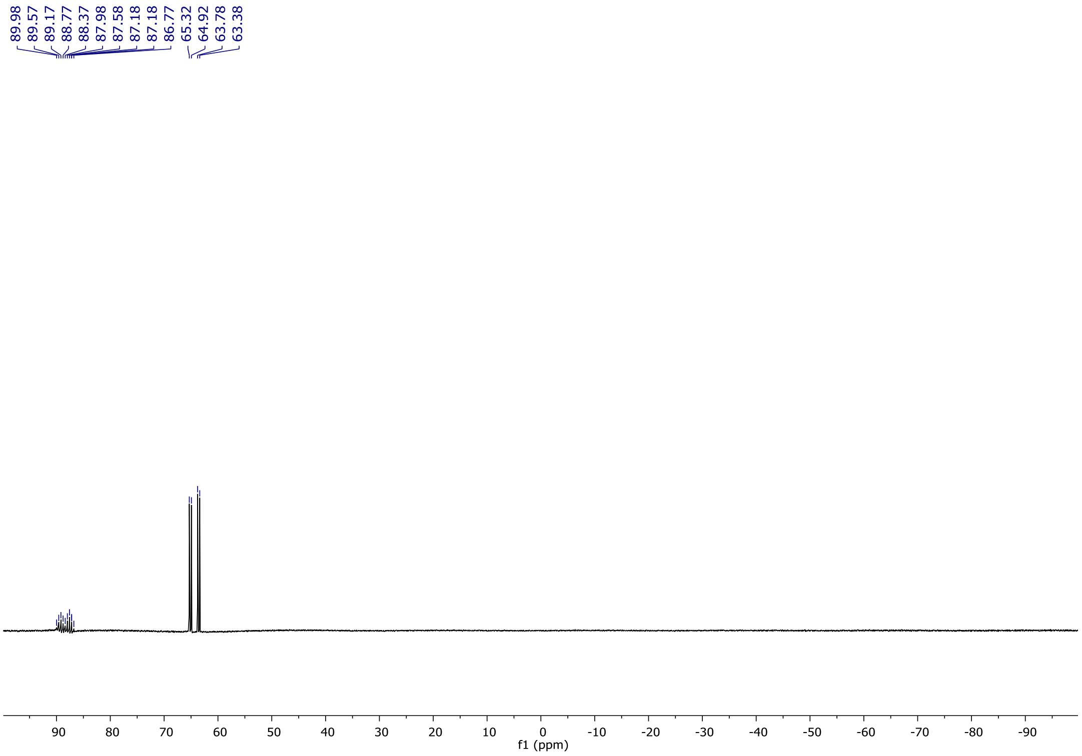
89.98
89.57
89.17
88.77
88.37
87.98
87.58
87.18
87.18
86.77
65.32
64.92
63.78
63.38
90 80 70 60 50 40 30 20 10 0 -10 -20 -30 -40 -50 -60 -70 -80 -90
f1 (ppm)

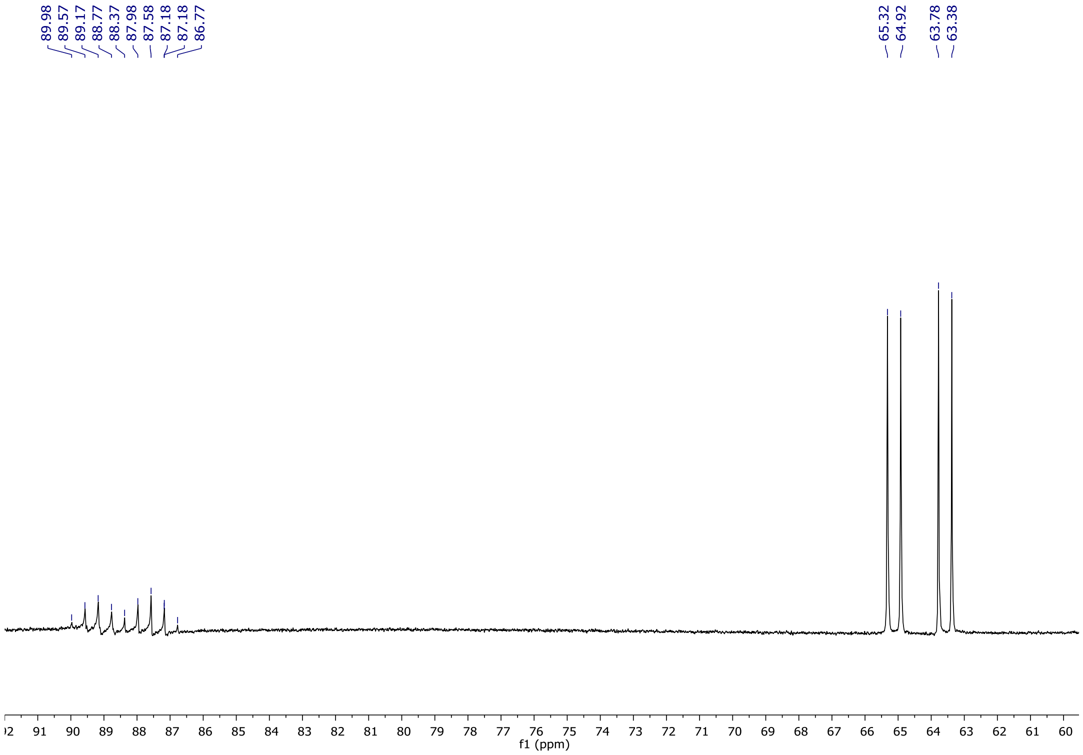
89.98
89.57
89.17
88.77
88.37
87.98
87.58
87.18
87.18
86.77
65.32
64.92
63.78
63.38
91 90 89 88 87 86 85 84 83 82 81 80 79 78 77 76 75 74 73 72 71 70 69 68 67 66 65 64 63 62 61 60
f1 (ppm)

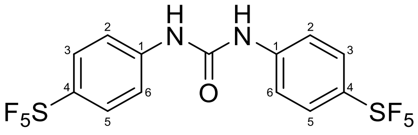

H
N
H
N
1
2
3
4
5
6
O
F5S
SF5

10

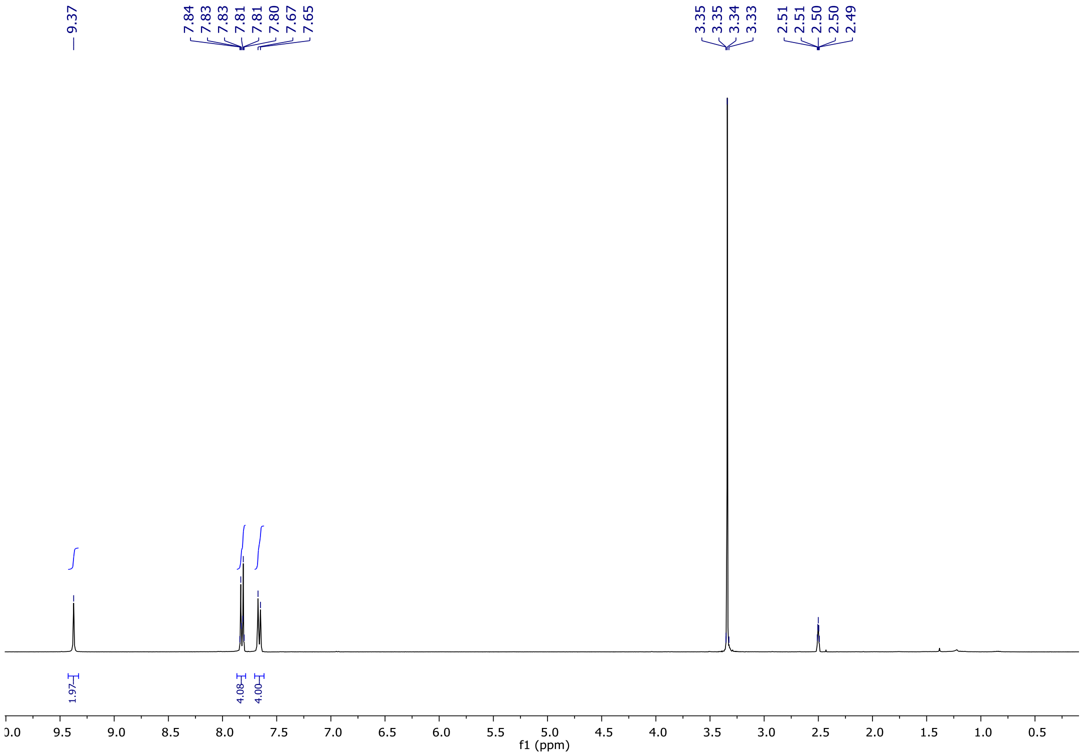

9.37
7.84
7.83
7.83
7.81
7.81
7.80
7.67
7.65
3.35
3.35
3.34
3.33
2.51
2.51
2.50
2.50
2.49
1.97
4.08
4.00
f1 (ppm)

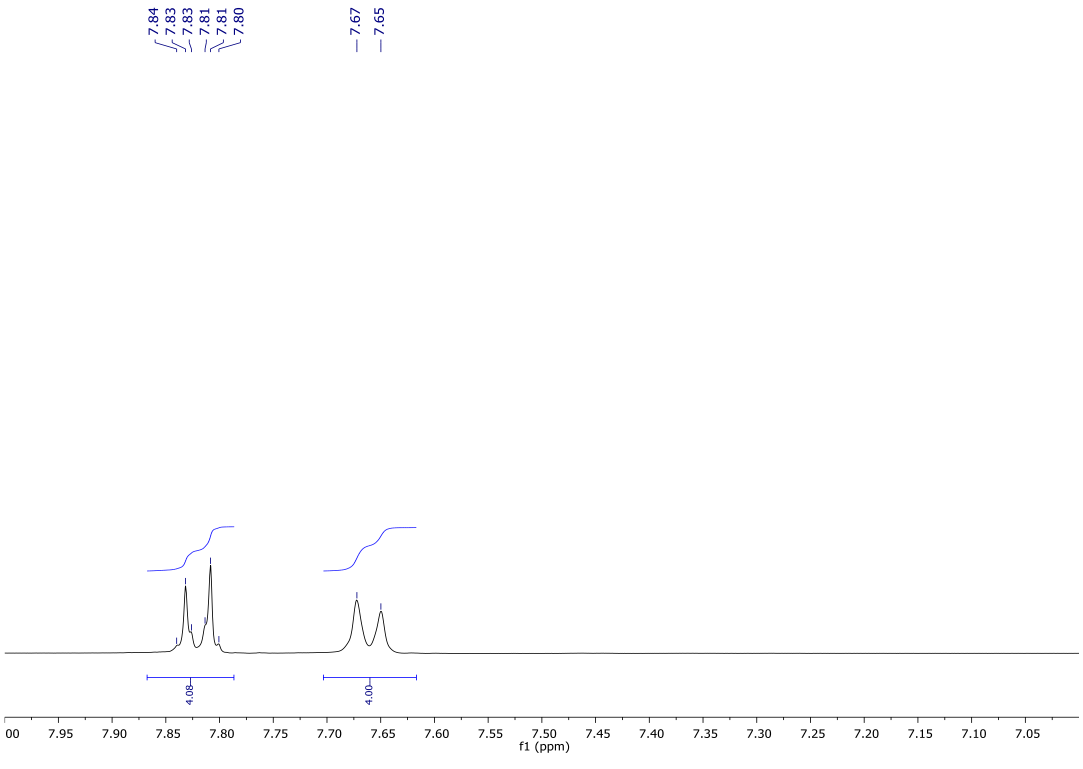

7.84
7.83
7.83
7.81
7.81
7.80
7.67
7.65
4.08
4.00
f1 (ppm)

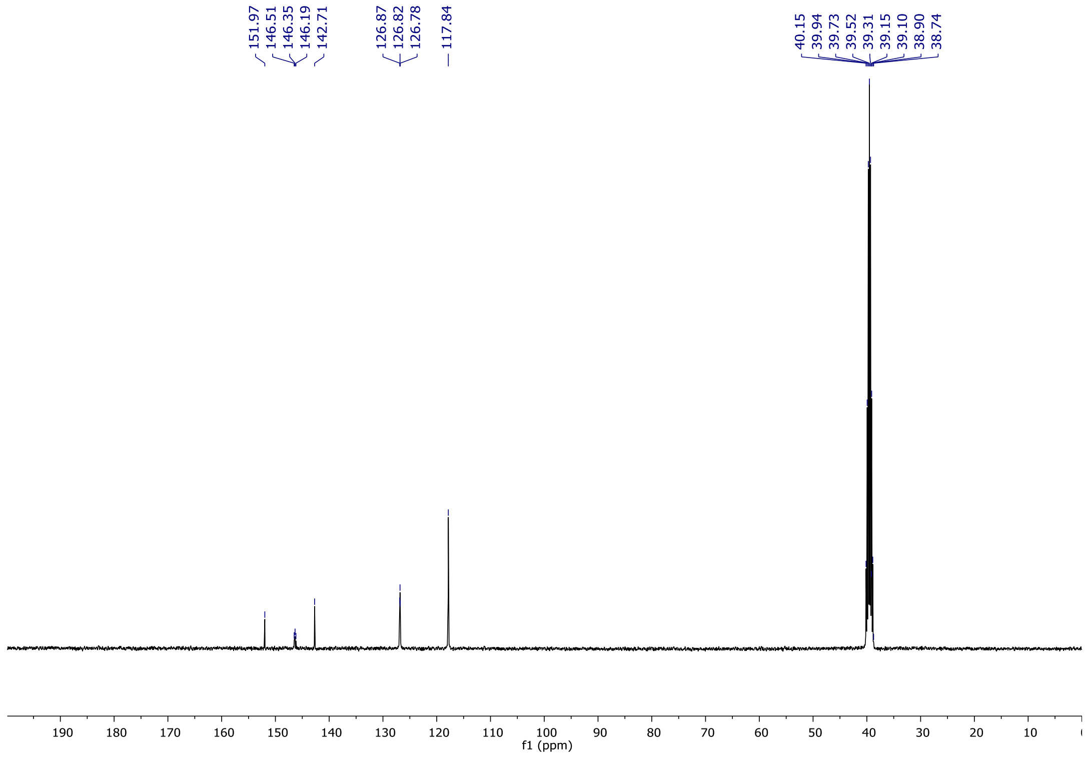
151.97
146.51
146.35
146.19
142.71
126.87
126.82
126.78
117.84
40.15
39.94
39.73
39.52
39.31
39.15
39.10
38.90
38.74
190 180 170 160 150 140 130 120 110 100 90 80 70 60 50 40 30 20 10
f1 (ppm)

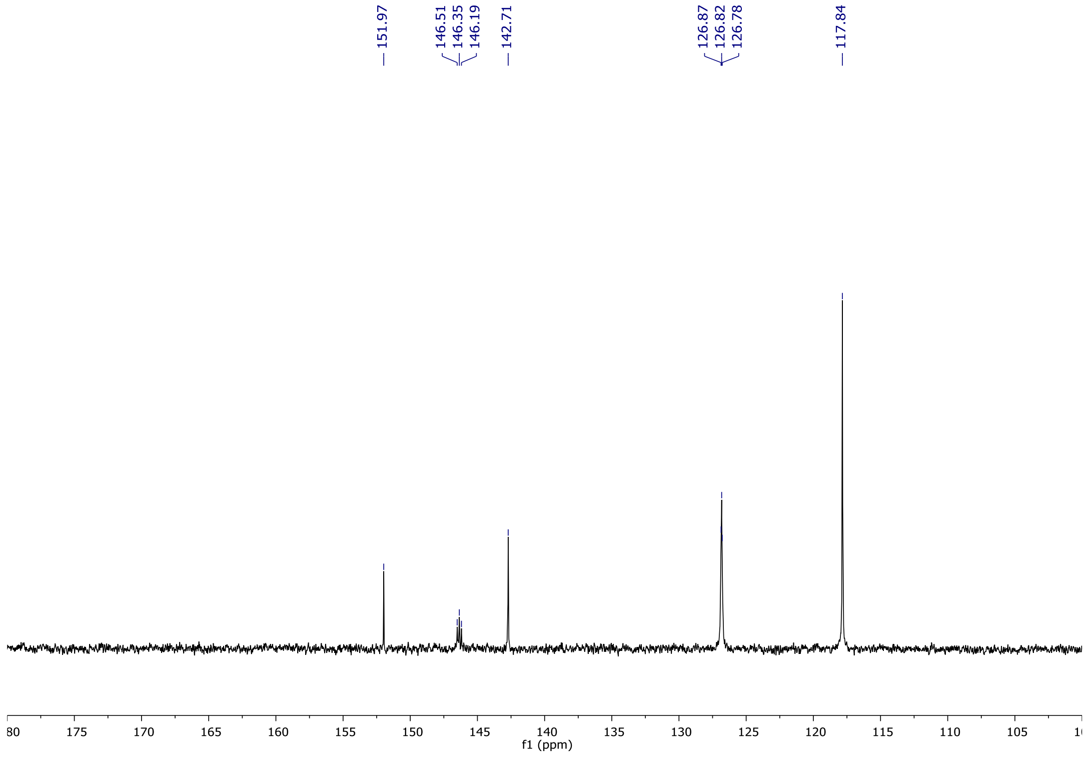
151.97
146.51
146.35
146.19
142.71
126.87
126.82
126.78
117.84
175 170 165 160 155 150 145 140 135 130 125 120 115 110 105
f1 (ppm)

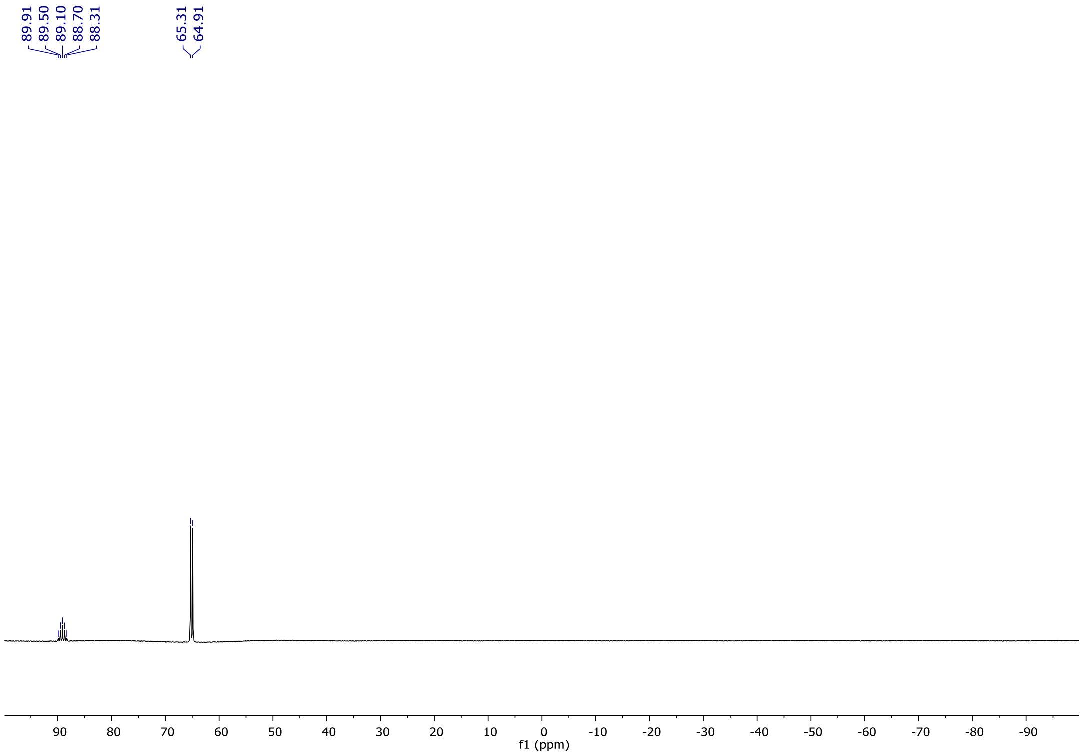
89.91
89.50
89.10
88.70
88.31
65.31
64.91
90 80 70 60 50 40 30 20 10 0 -10 -20 -30 -40 -50 -60 -70 -80 -90
f1 (ppm)

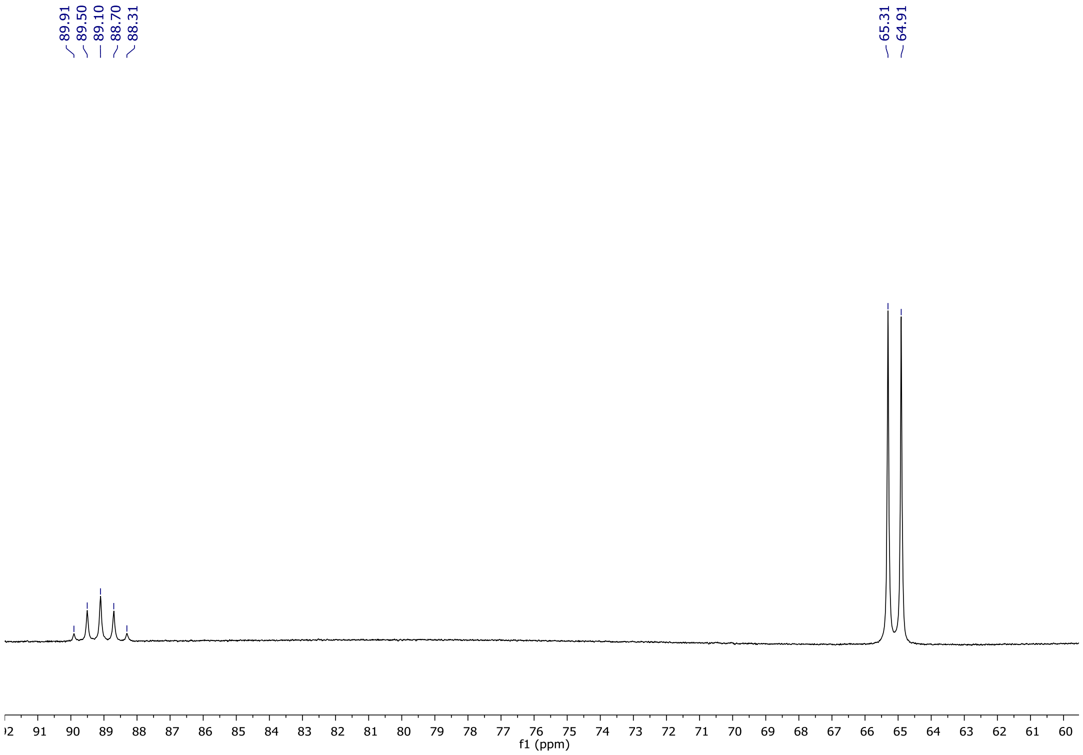
89.91
89.50
89.10
88.70
88.31
65.31
64.91
91 90 89 88 87 86 85 84 83 82 81 80 79 78 77 76 75 74 73 72 71 70 69 68 67 66 65 64 63 62 61 60
f1 (ppm)

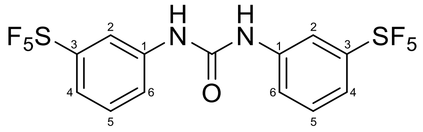

11

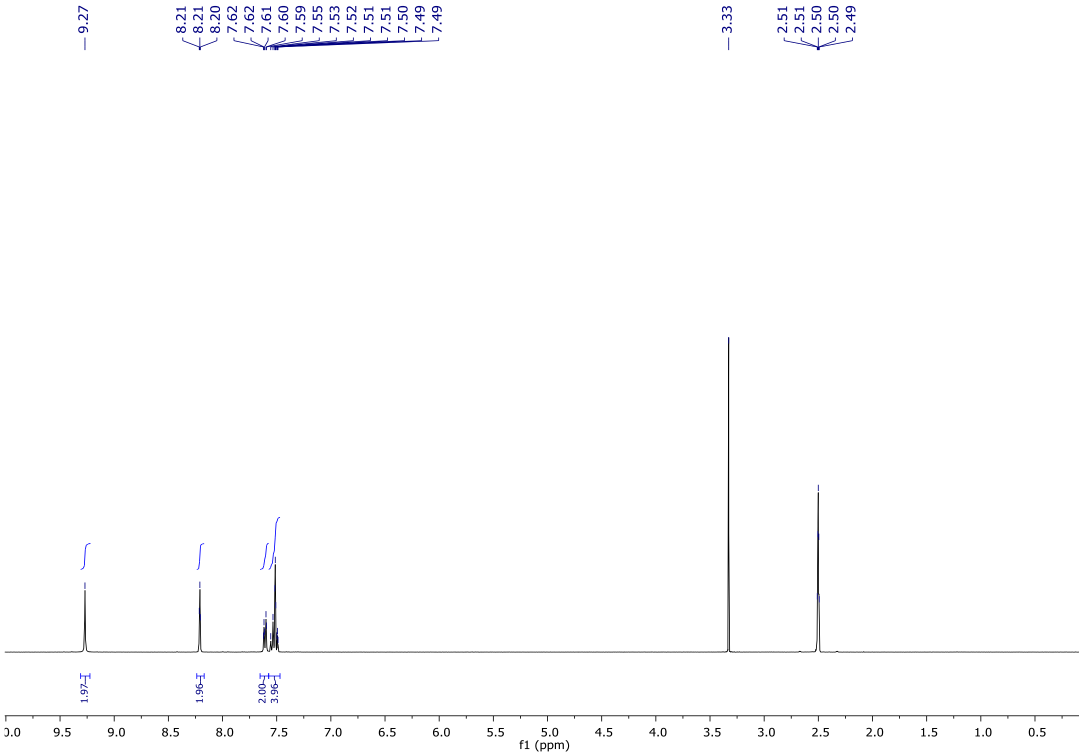
9.27
8.21
8.21
8.20
7.62
7.62
7.61
7.60
7.59
7.55
7.53
7.52
7.51
7.51
7.50
7.49
7.49
3.33
2.51
2.51
2.50
2.50
2.49
1.97
1.96
2.00
3.96
f1 (ppm)

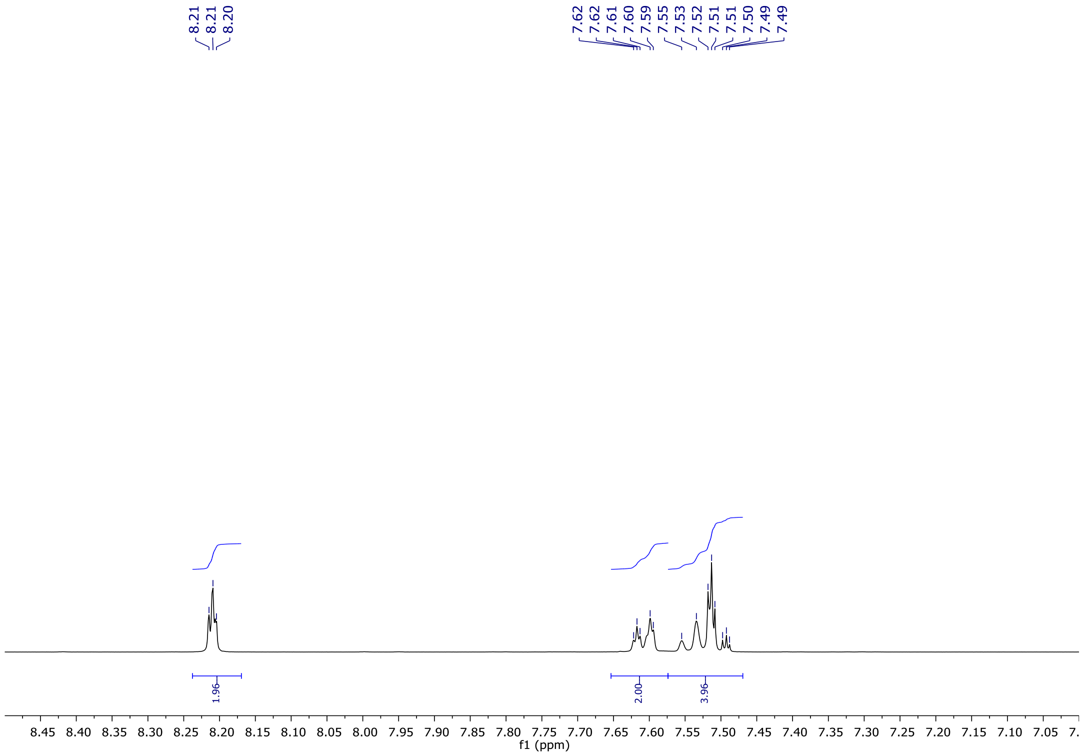
8.21
8.21
8.20
7.62
7.62
7.61
7.60
7.59
7.55
7.53
7.52
7.51
7.51
7.50
7.49
7.49
1.96
2.00
3.96
f1 (ppm)

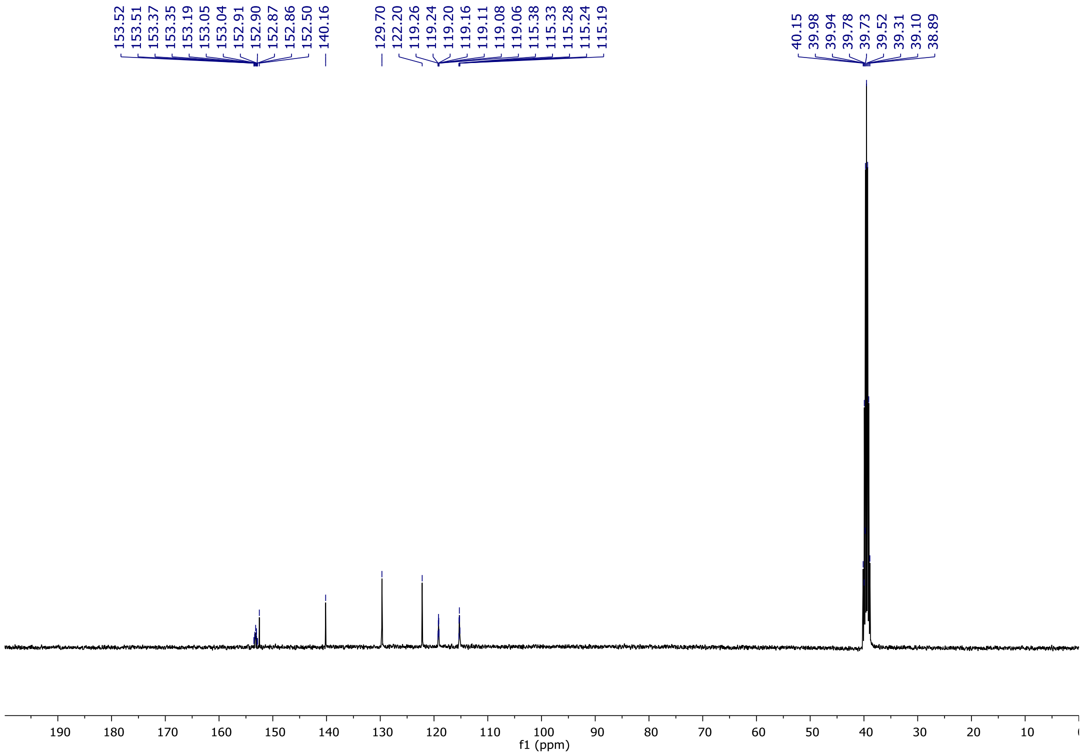
153.52
153.51
153.37
153.35
153.19
153.05
153.04
152.91
152.90
152.87
152.86
152.50
140.16
129.70
122.20
119.26
119.24
119.20
119.16
119.11
119.08
119.06
115.38
115.33
115.28
115.24
115.19
40.15
39.98
39.94
39.78
39.73
39.52
39.31
39.10
38.89
190 180 170 160 150 140 130 120 110 100 90 80 70 60 50 40 30 20 10
f1 (ppm)

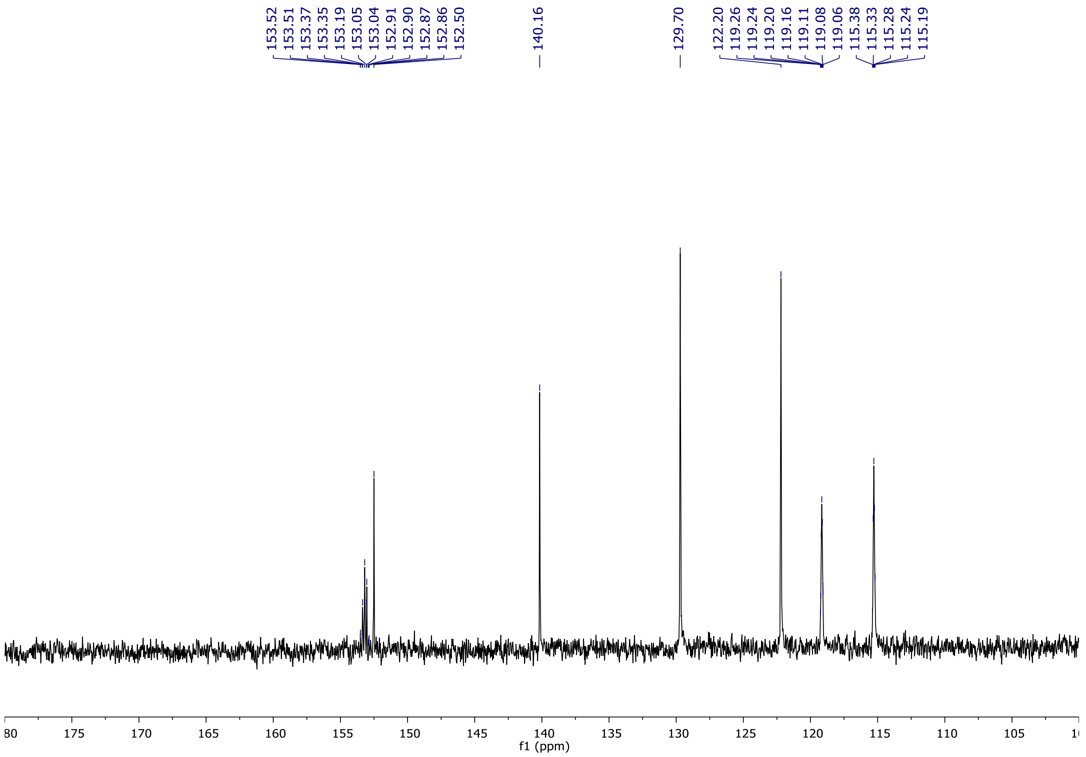
153.52
153.51
153.37
153.35
153.19
153.05
153.04
152.91
152.90
152.87
152.86
152.50
140.16
129.70
122.20
119.26
119.24
119.20
119.16
119.11
119.08
119.06
115.38
115.33
115.28
115.24
115.19
175 170 165 160 155 150 145 140 135 130 125 120 115 110 105
f1 (ppm)

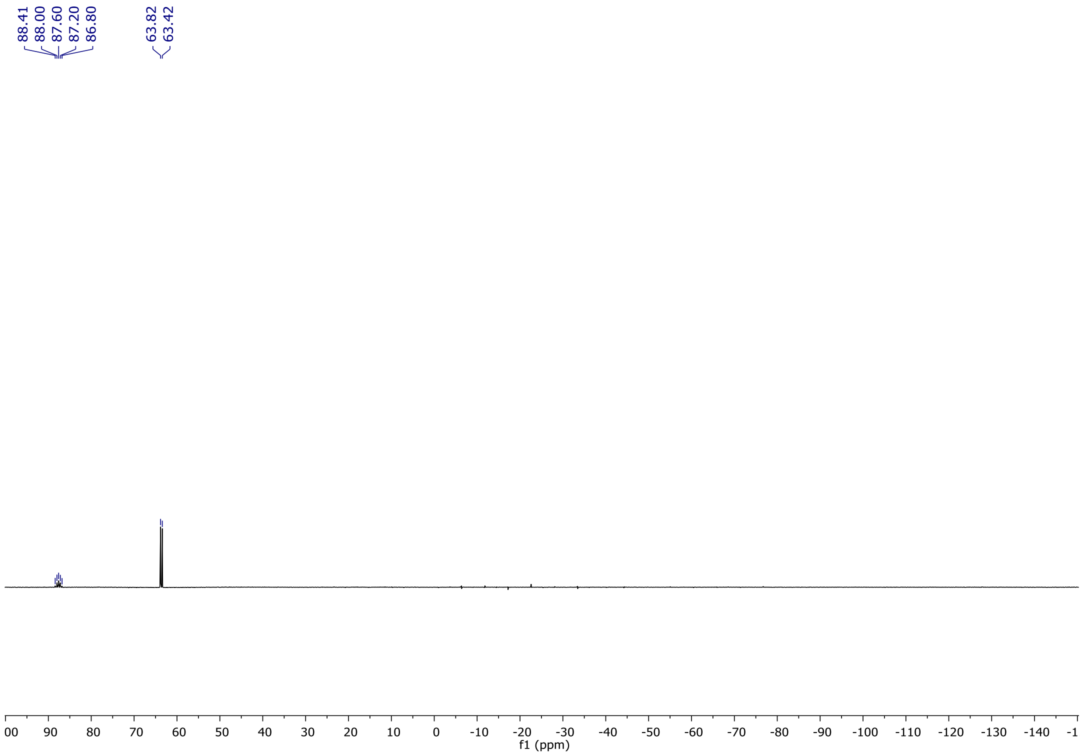
88.41
88.00
87.60
87.20
86.80
63.82
63.42
00 90 80 70 60 50 40 30 20 10 0 -10 -20 -30 -40 -50 -60 -70 -80 -90 -100 -110 -120 -130 -140 -1
f1 (ppm)

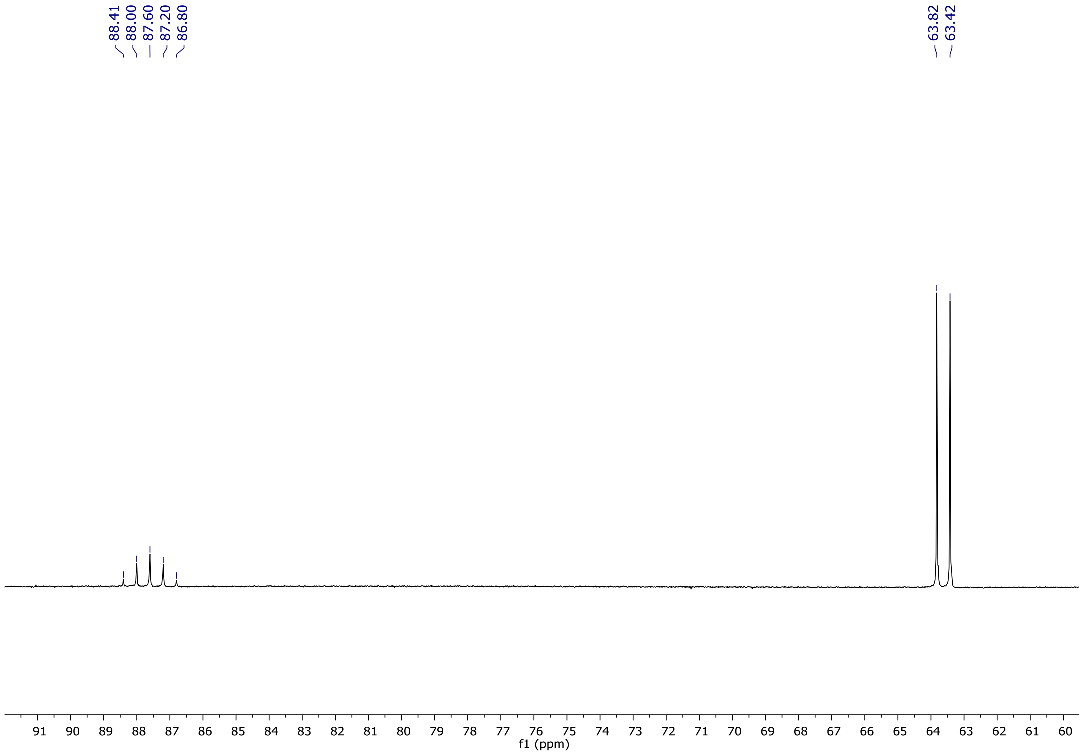
88.41
88.00
87.60
87.20
86.80
63.82
63.42
91 90 89 88 87 86 85 84 83 82 81 80 79 78 77 76 75 74 73 72 71 70 69 68 67 66 65 64 63 62 61 60
f1 (ppm)

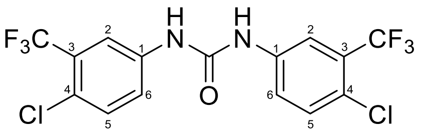
F3C
CF3
Cl
Cl
H
N
H
N
O
1
2
3
4
5
6

12

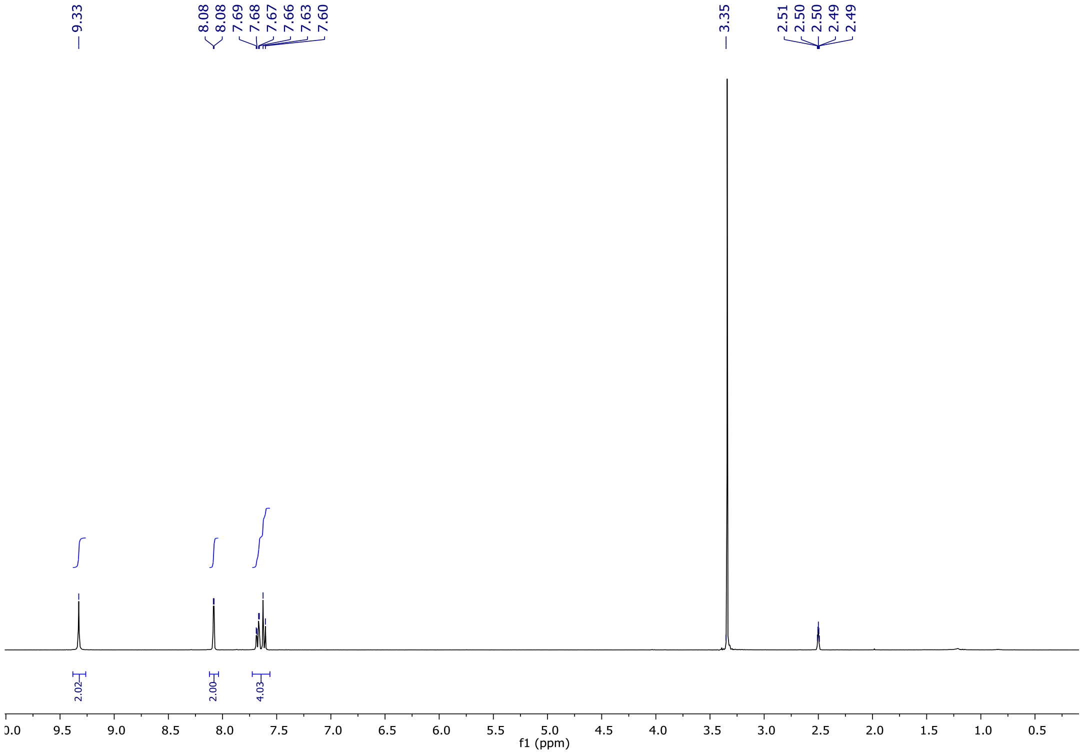
9.33
8.08
8.08
7.69
7.68
7.67
7.66
7.63
7.60
3.35
2.51
2.50
2.50
2.49
2.49
2.02
2.00
4.03
f1 (ppm)

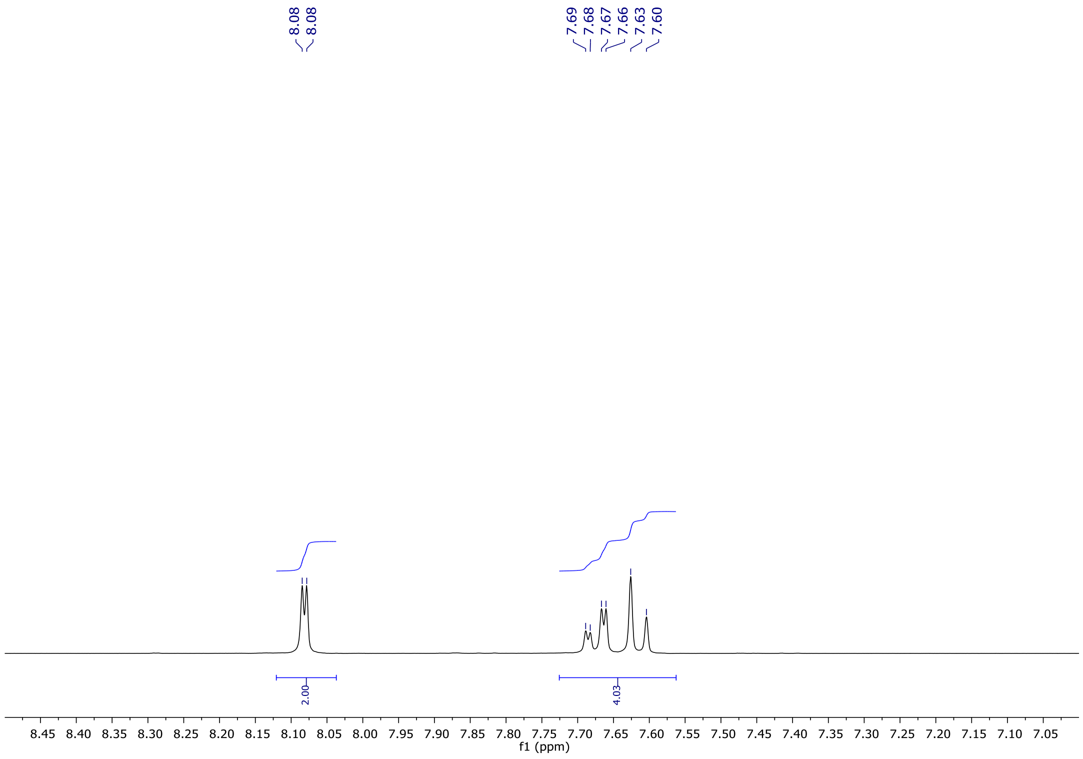
8.08
8.08
7.69
7.68
7.67
7.66
7.63
7.60
2.00
4.03
f1 (ppm)

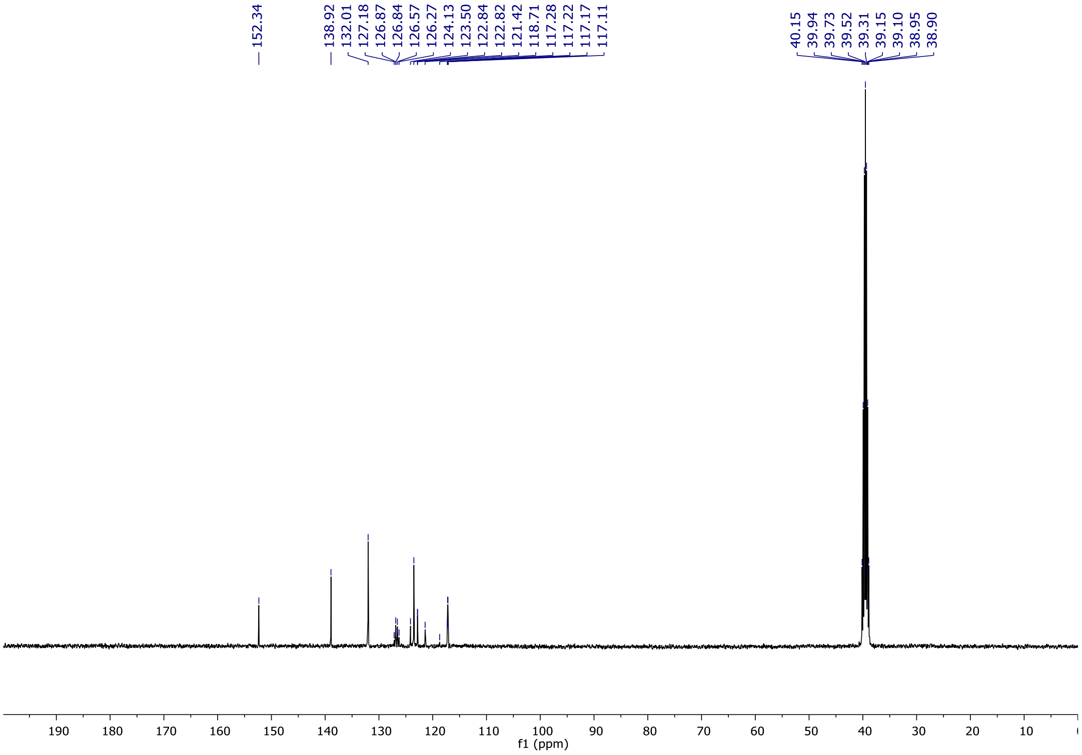
152.34
138.92
132.01
127.18
126.87
126.84
126.57
126.27
124.13
123.50
122.84
122.82
121.42
118.71
117.28
117.22
117.17
117.11
40.15
39.94
39.73
39.52
39.31
39.15
39.10
38.95
38.90
190 180 170 160 150 140 130 120 110 100 90 80 70 60 50 40 30 20 10
f1 (ppm)

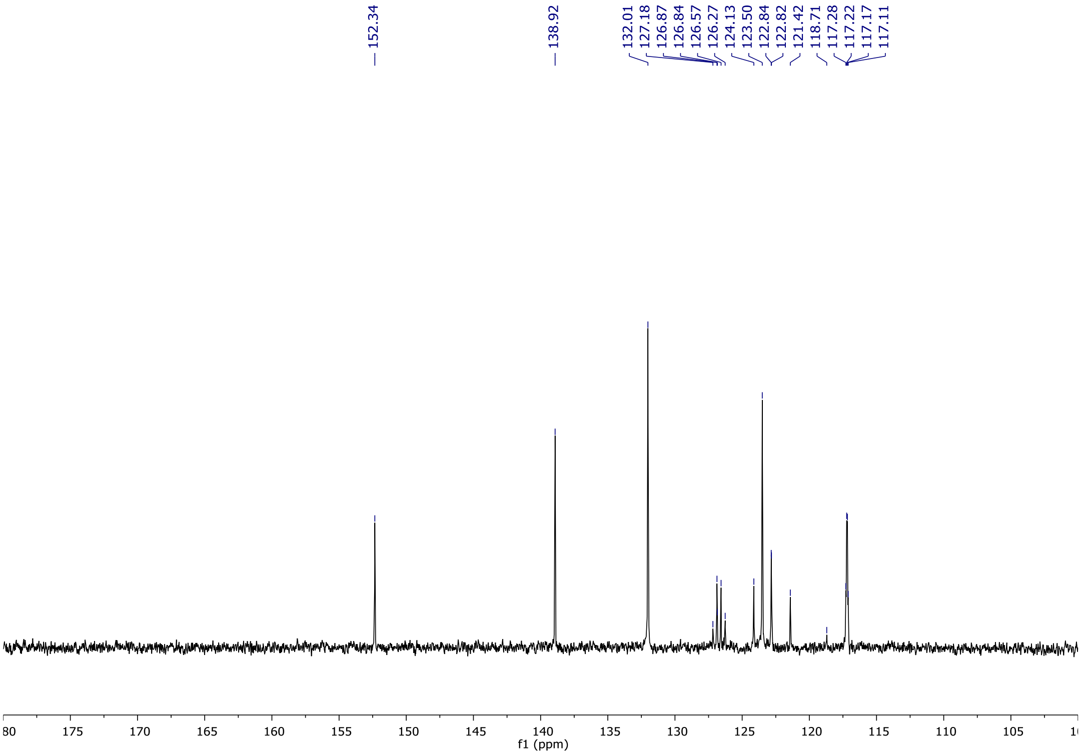
152.34
138.92
132.01
127.18
126.87
126.84
126.57
126.27
124.13
123.50
122.84
122.82
121.42
118.71
117.28
117.22
117.17
117.11
175 170 165 160 155 150 145 140 135 130 125 120 115 110 105
f1 (ppm)

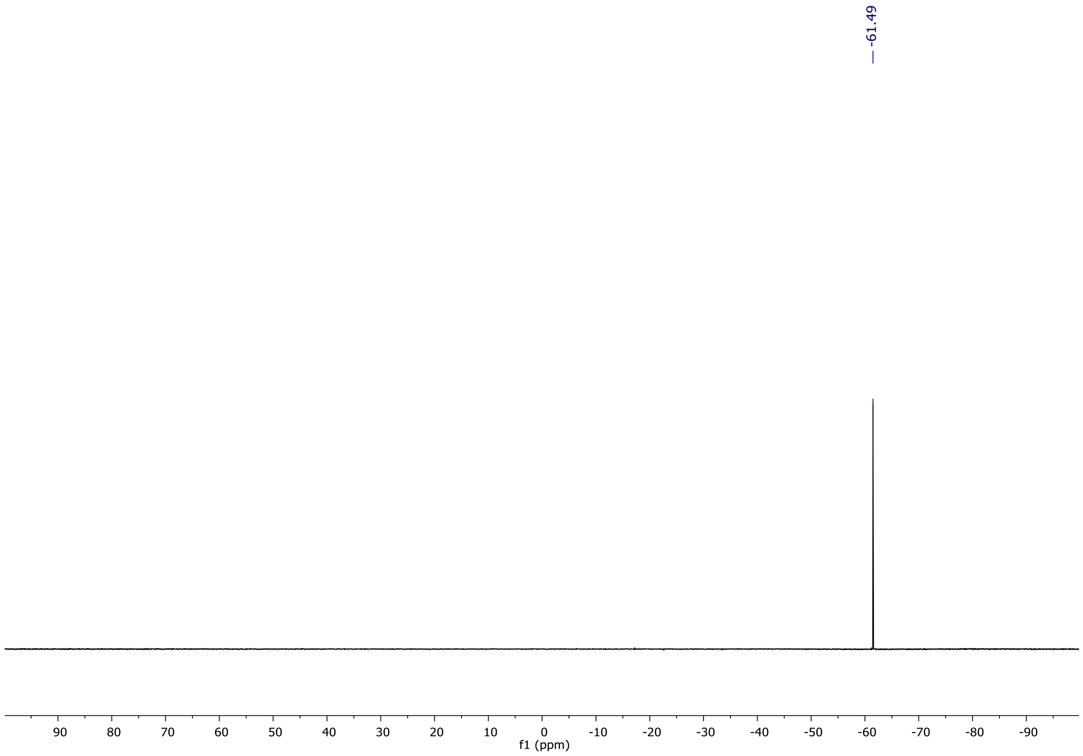
-61.49
90 80 70 60 50 40 30 20 10 0 -10 -20 -30 -40 -50 -60 -70 -80 -90
f1 (ppm)

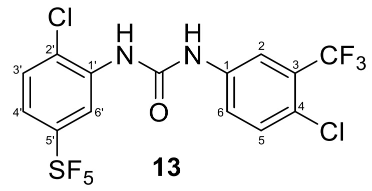
Cl
H
N
H
N
2
3
CF3
2'
1'
1
3'
4'
6'
O
6
4
Cl
5
5'
SF5

13

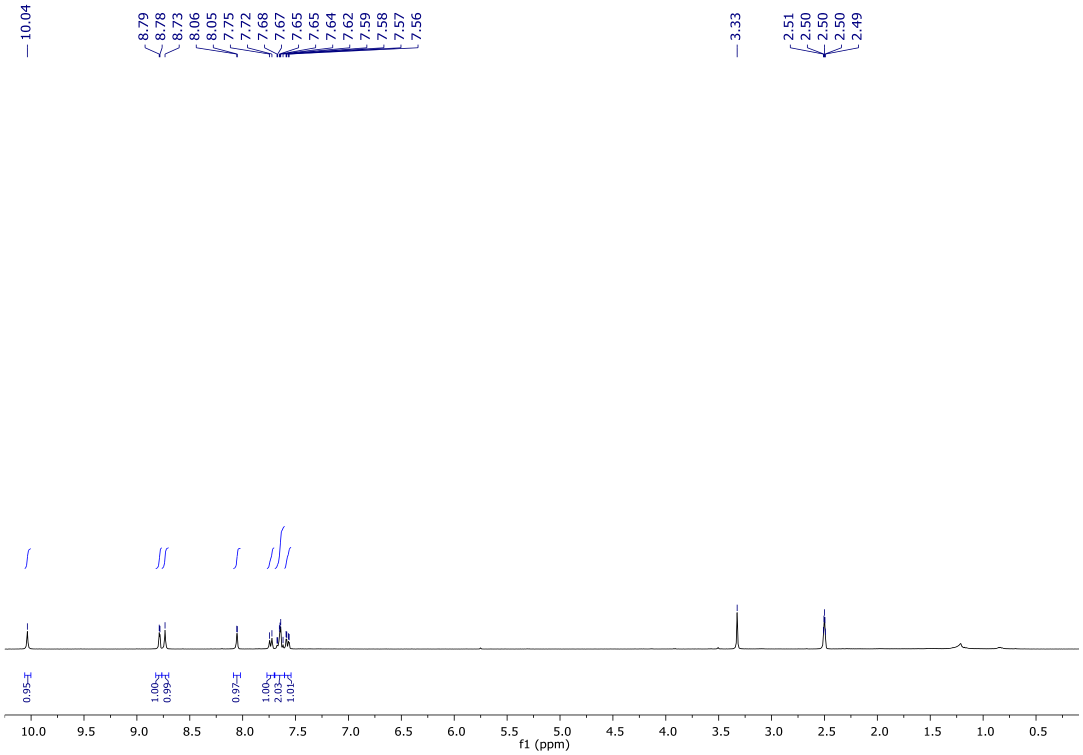
10.04
8.79
8.78
8.73
8.06
8.05
7.75
7.72
7.68
7.67
7.65
7.64
7.62
7.59
7.58
7.57
7.56
3.33
2.51
2.50
2.50
2.50
2.49
0.95
1.00
0.99
0.97
1.00
2.03
1.01
f1 (ppm)

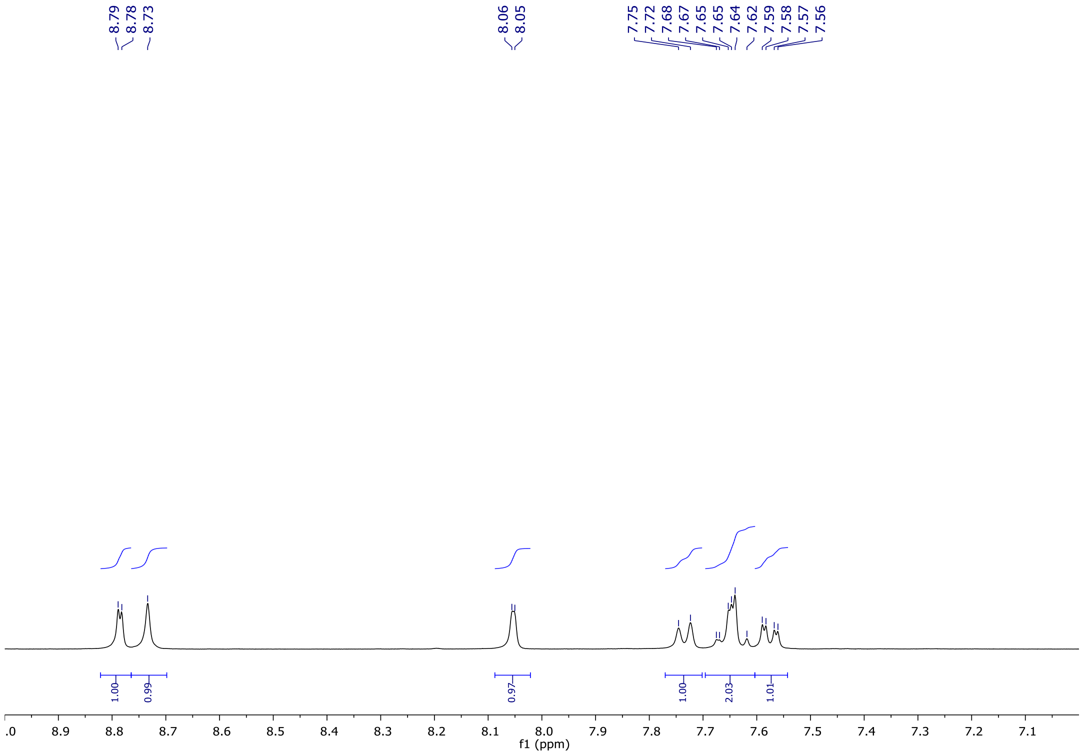
8.79
8.78
8.73
8.06
8.05
7.75
7.72
7.68
7.67
7.65
7.65
7.64
7.62
7.59
7.58
7.57
7.56
1.00
0.99
0.97
1.00
2.03
1.01
f1 (ppm)

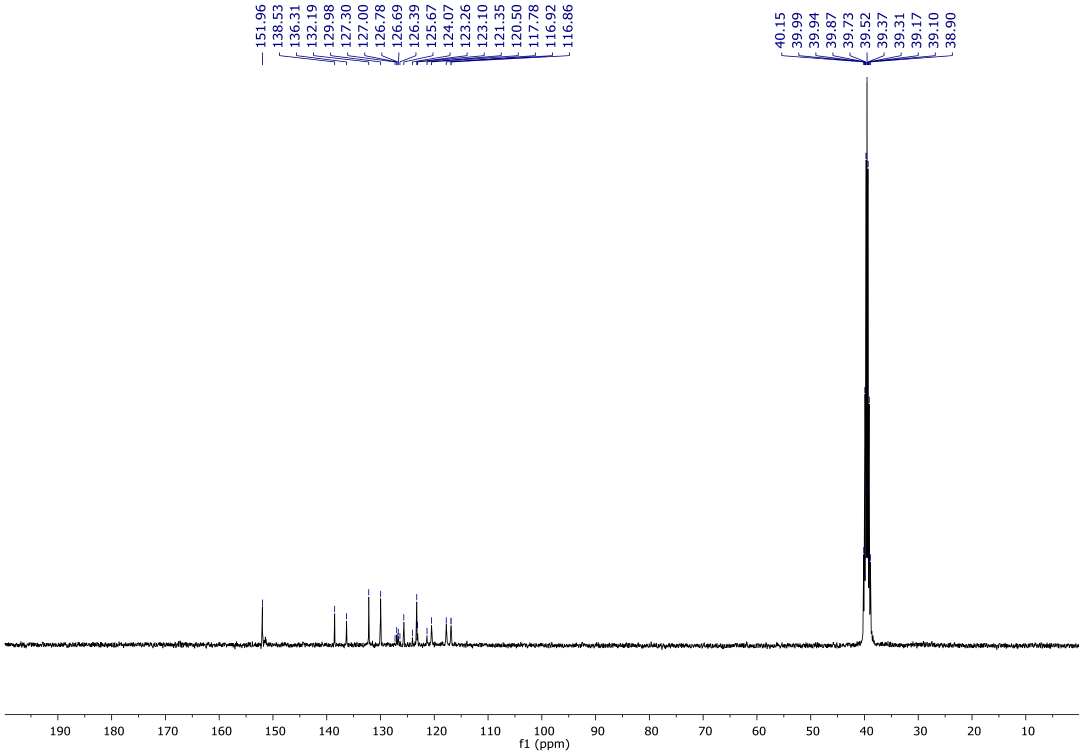
151.96
138.53
136.31
132.19
129.98
127.30
127.00
126.78
126.69
126.39
125.67
124.07
123.26
123.10
121.35
120.50
117.78
116.92
116.86
40.15
39.99
39.94
39.87
39.73
39.52
39.37
39.31
39.17
39.10
38.90
f1 (ppm)

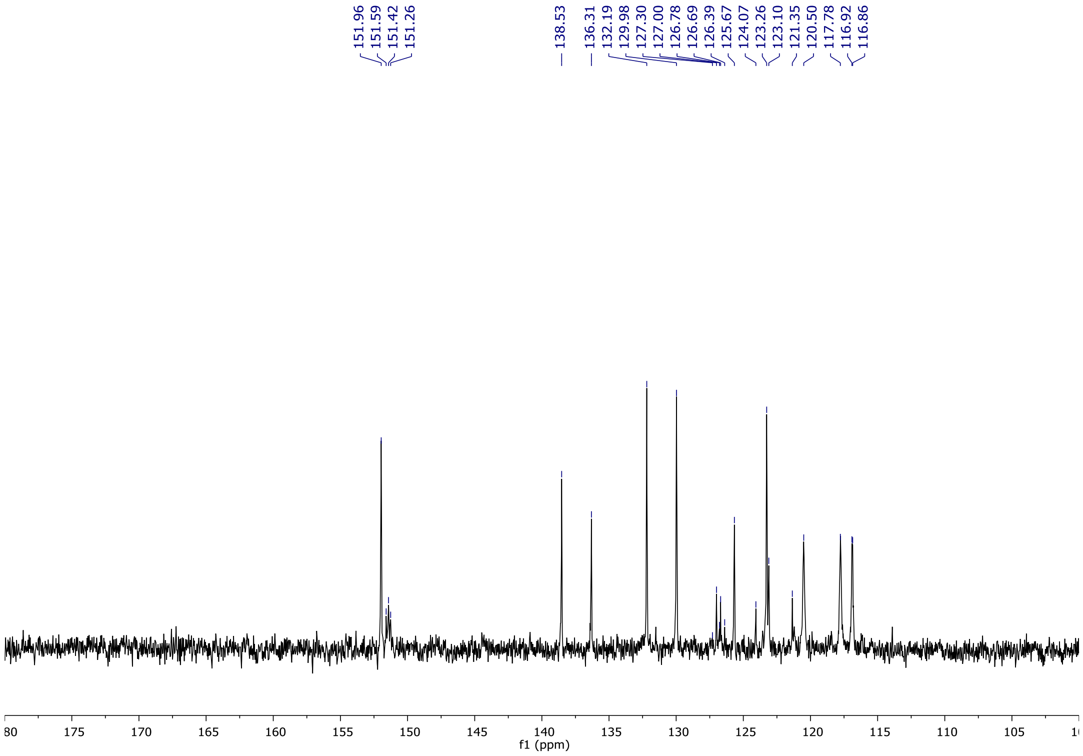
151.96
151.59
151.42
151.26
138.53
136.31
132.19
129.98
127.30
127.00
126.78
126.69
126.39
125.67
124.07
123.26
123.10
121.35
120.50
117.78
116.92
116.86
f1 (ppm)

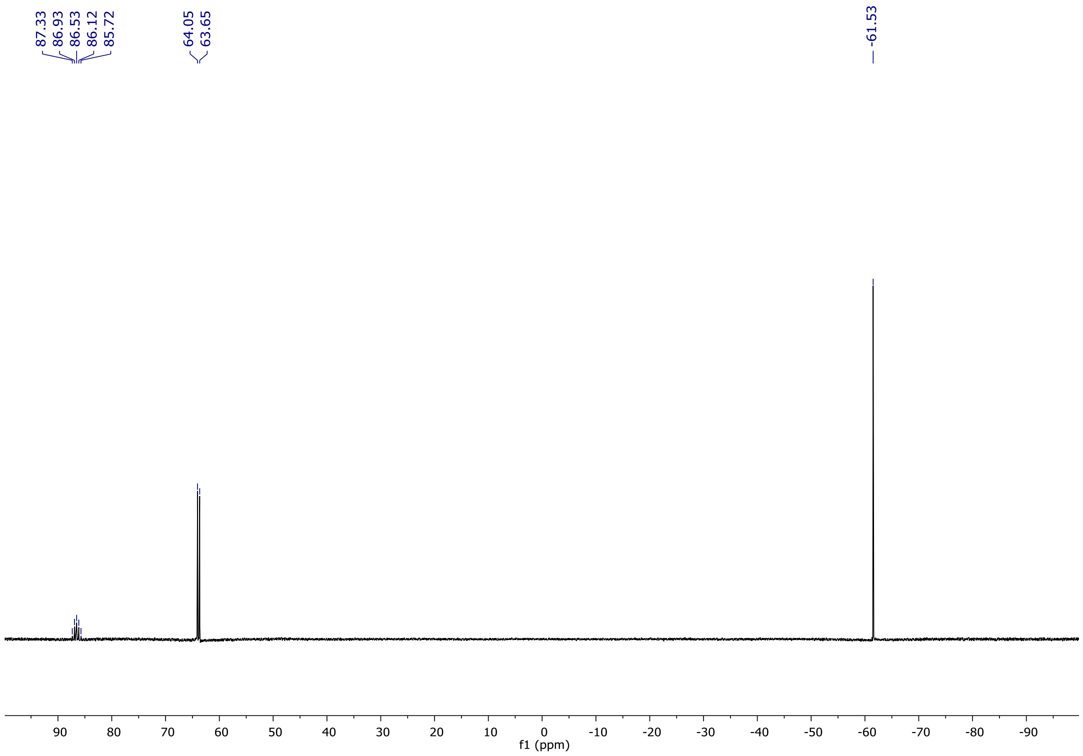

87.33
86.93
86.53
86.12
85.72
64.05
63.65
-61.53
90 80 70 60 50 40 30 20 10 0 -10 -20 -30 -40 -50 -60 -70 -80 -90
f1 (ppm)

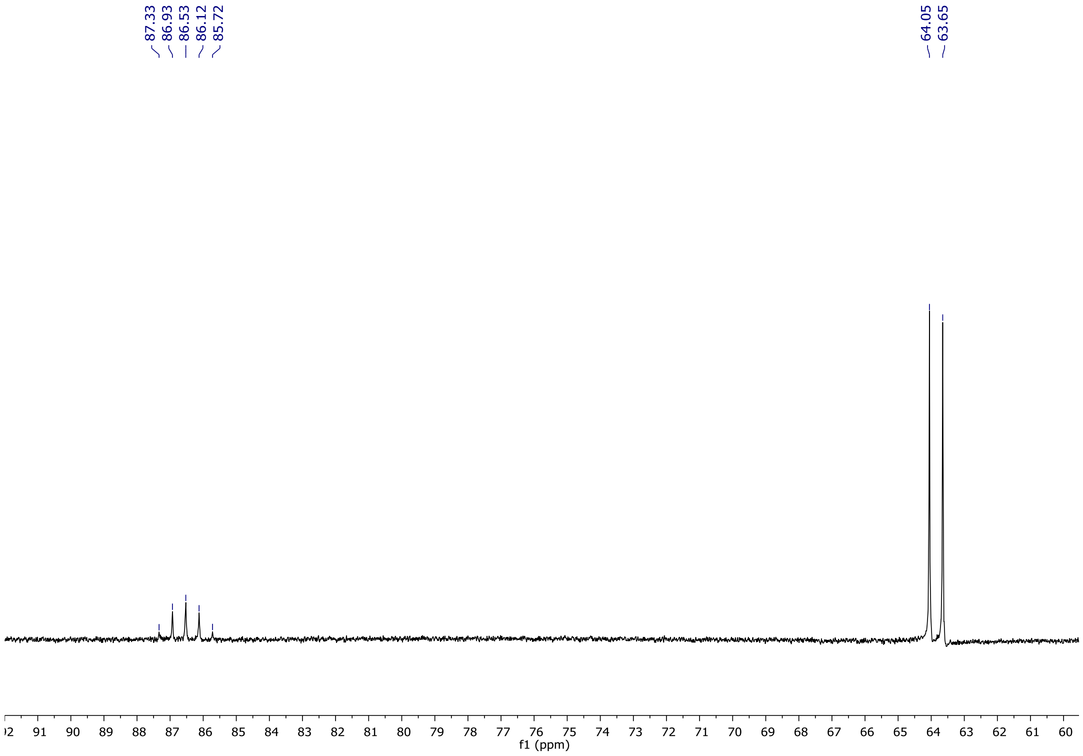

87.33
86.93
86.53
86.12
85.72
64.05
63.65
91 90 89 88 87 86 85 84 83 82 81 80 79 78 77 76 75 74 73 72 71 70 69 68 67 66 65 64 63 62 61 60
f1 (ppm)

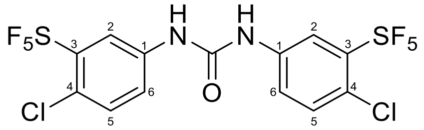
F5S
SF5
Cl
Cl
H
N
O

14

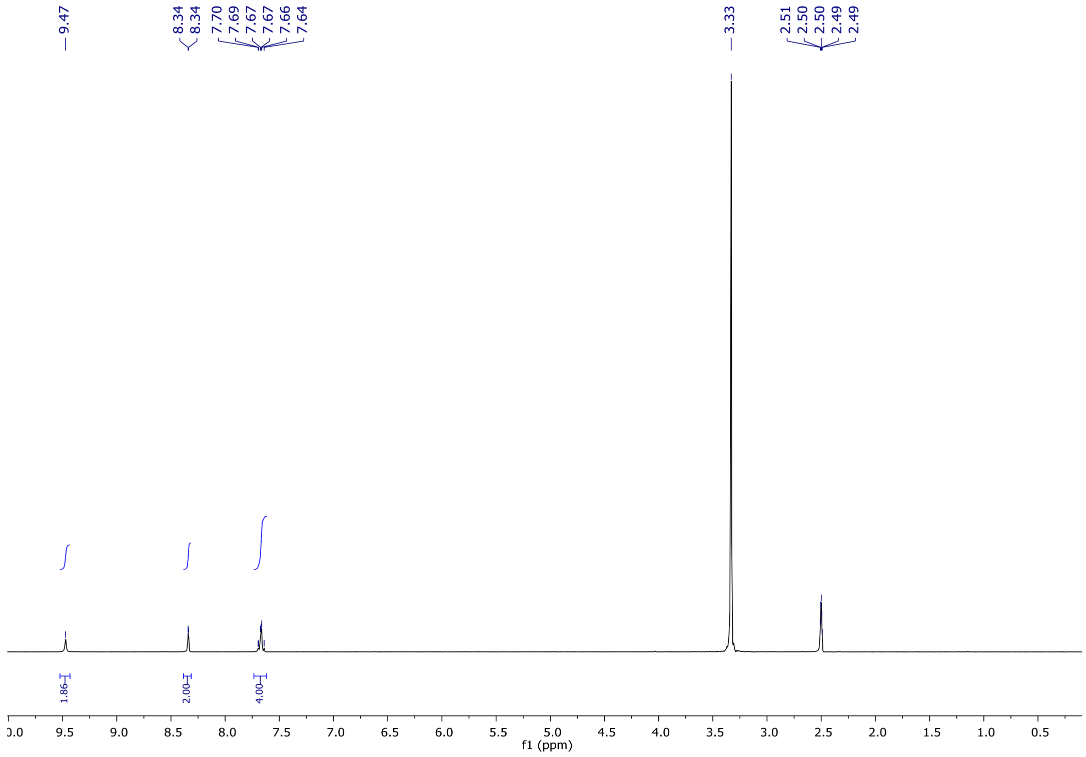
9.47
8.34 8.34
7.70 7.69 7.67 7.67 7.66 7.64
3.33
2.51 2.50 2.50 2.49 2.49
1.86
2.00
4.00
f1 (ppm)

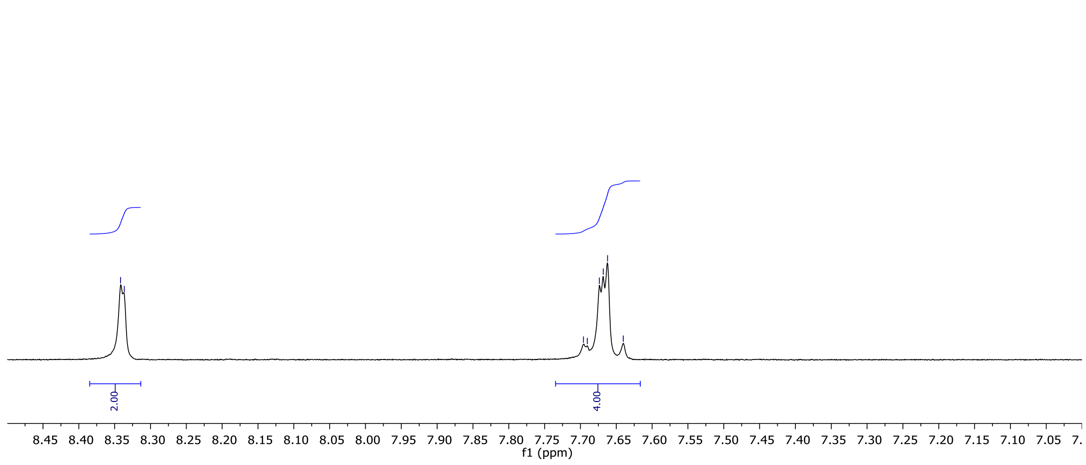
8.34 8.34
7.70 7.69 7.67 7.67 7.66 7.64
2.00
4.00
f1 (ppm)

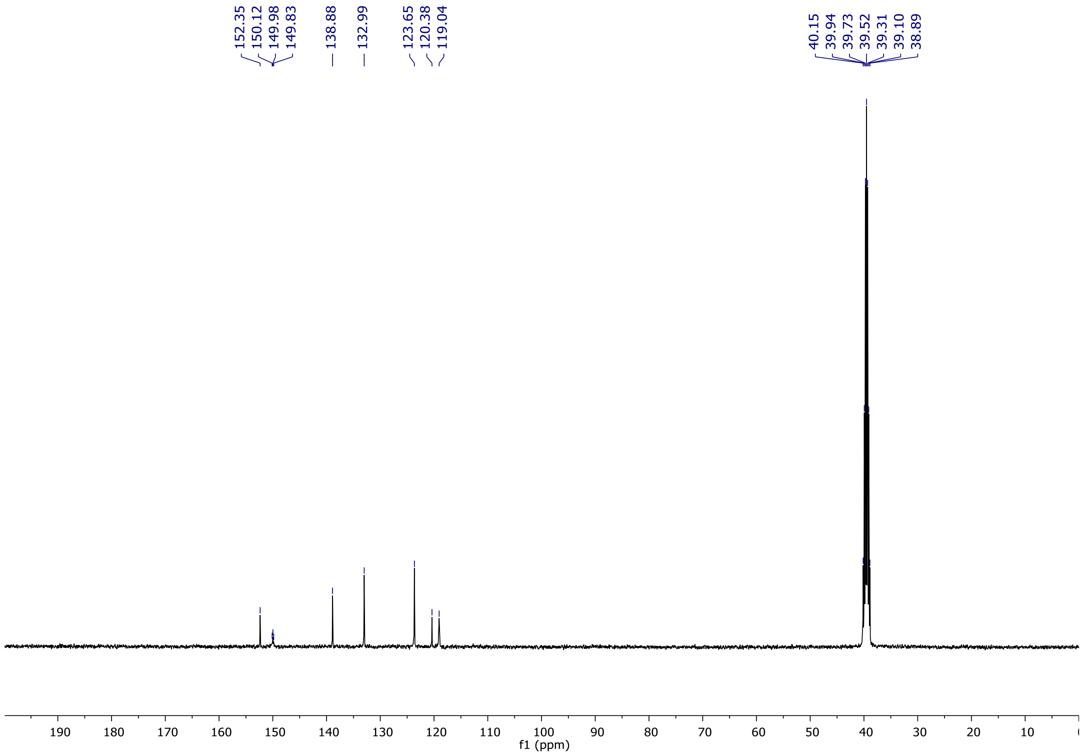
152.35
150.12
149.98
149.83
138.88
132.99
123.65
120.38
119.04
40.15
39.94
39.73
39.52
39.31
39.10
38.89
f1 (ppm)

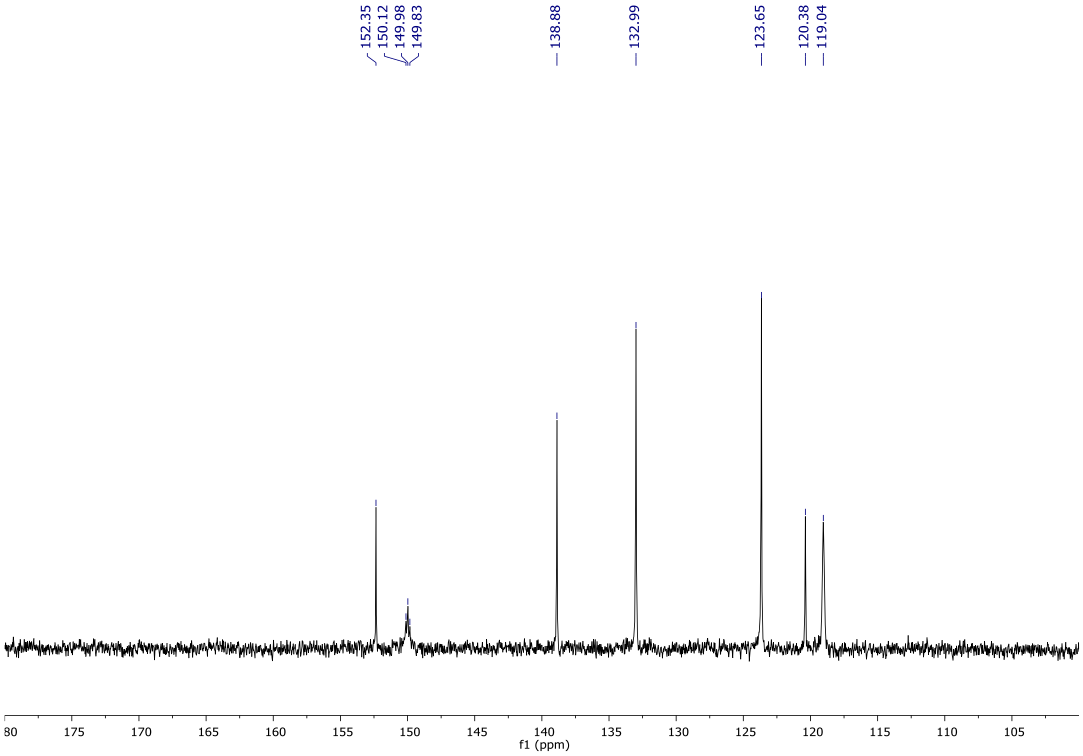
152.35
150.12
149.98
149.83
138.88
132.99
123.65
120.38
119.04
f1 (ppm)

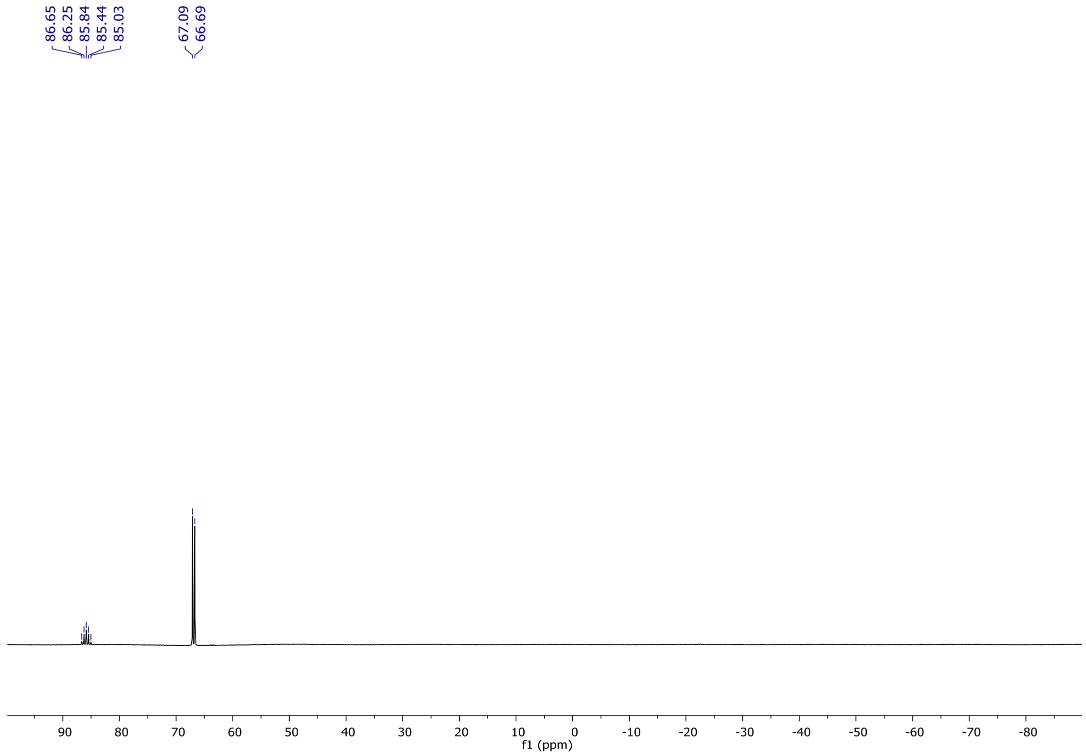
86.65
86.25
85.84
85.44
85.03
67.09
66.69
90 80 70 60 50 40 30 20 10 0 -10 -20 -30 -40 -50 -60 -70 -80
f1 (ppm)

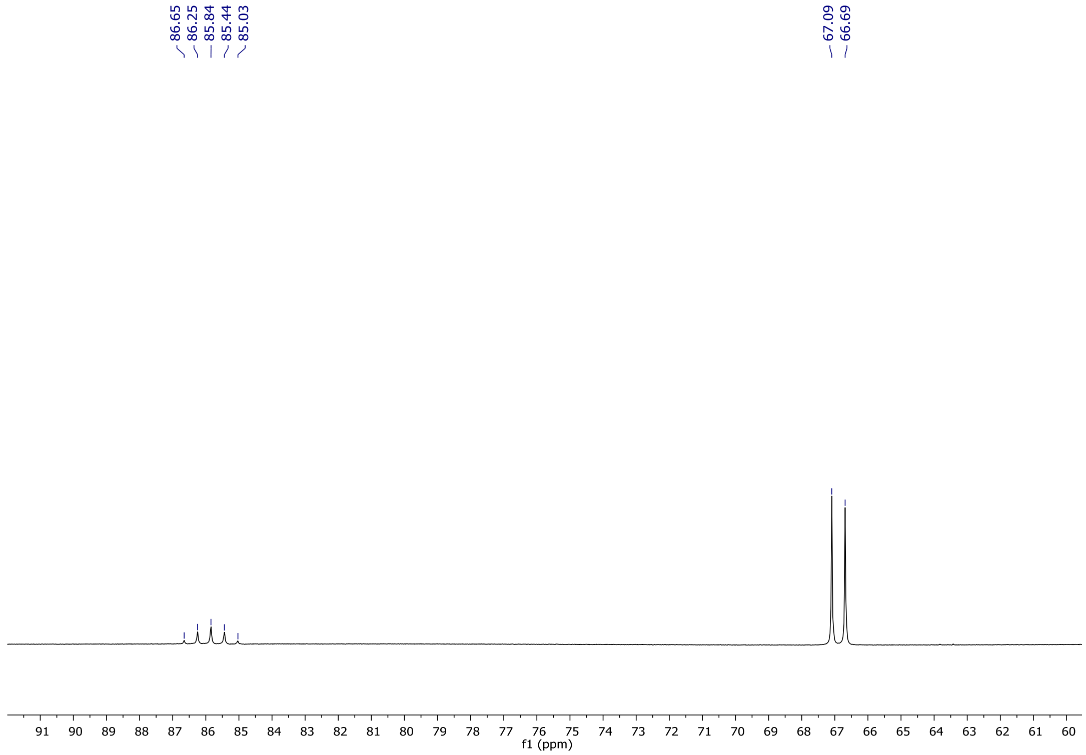
86.65
86.25
85.84
85.44
85.03
67.09
66.69
91 90 89 88 87 86 85 84 83 82 81 80 79 78 77 76 75 74 73 72 71 70 69 68 67 66 65 64 63 62 61 60
f1 (ppm)
